# Supplementary material for: Impact of preterm birth on brain development and long-term outcome: protocol for a cohort study in Scotland
Source: BMJ Open. 2020 Mar 4;10(3):e035854. doi: 10.1136/bmjopen-2019-035854 (PMC7059503; doi:10.1136/bmjopen-2019-035854)
Supplement: Supplementary data [file bmjopen-2019-035854supp001.pdf]

SIEMENS MAGNETOM Prisma

Table of contents

\\Study Protocols
BRAIN
Neonates
Theirworld - E161723
localizer_neonate
t2_haste_localiser
t2_blade_v3
t2_space_sag_p4_iso_v2x
DTI_Neonate_v6b_dummy
DTI_Neonate_v6b_rev
DTI_Neonate_v6b_pt1
DTI_Neonate_v6b_pt2
MPRAGE-v4
SWI_v2
t2_blade_dark-fluid_tra_v3

Boardman JP, *et al. BMJ Open* 2020; 10:e035854. doi: 10.1136/bmjopen-2019-035854

SIEMENS MAGNETOM Prisma

\\Study Protocols\BRAIN\Neonates\Theirworld - E161723\localizer_neonate

TA: 0:12 PM: REF Voxel size: 0.5×0.5×7.0 mmPAT: Off Rel. SNR: 1.00 : fl

Properties

Prio recon	On
Load images to viewer	On
Inline movie	Off
Auto store images	On
Load images to stamp segments	On
Load images to graphic segments	On
Auto open inline display	Off
Auto close inline display	Off
Start measurement without further preparation	Off
Wait for user to start	Off
Start measurements	Single measurement

Routine

Slice group	1
Slices	1
Dist. factor	20 %
Position	L0.0 P47.8 F62.3 mm
Orientation	Sagittal
Phase enc. dir.	A >> P
Slice group	2
Slices	1
Dist. factor	20 %
Position	L0.0 P47.8 F62.3 mm
Orientation	Transversal
Phase enc. dir.	A >> P
Slice group	3
Slices	1
Dist. factor	20 %
Position	L0.0 P47.8 F62.3 mm
Orientation	Coronal
Phase enc. dir.	R >> L
AutoAlign	---
Phase oversampling	0 %
FoV read	250 mm
FoV phase	100.0 %
Slice thickness	7.0 mm
TR	7.5 ms
TE	3.69 ms
Averages	2
Concatenations	3
Filter	Prescan Normalize, Elliptical filter
Coil elements	PeH;PeN

Contrast - Common

TR	7.5 ms
TE	3.69 ms
TD	0 ms
MTC	Off
Magn. preparation	None
Flip angle	20 deg
Fat suppr.	None
Water suppr.	None
SWI	Off

Contrast - Dynamic

Averages	2
Averaging mode	Short term
Reconstruction	Magnitude
Measurements	1

Contrast - Dynamic

Multiple series	Each measurement

Resolution - Common

FoV read	250 mm
FoV phase	100.0 %
Slice thickness	7.0 mm
Base resolution	256
Phase resolution	91 %
Phase partial Fourier	Off
Interpolation	On

Resolution - iPAT

PAT mode	None

Resolution - Filter Image

Image Filter	Off
Distortion Corr.	Off
Prescan Normalize	On
Unfiltered images	Off
Normalize	Off
B1 filter	Off

Resolution - Filter Rawdata

Raw filter	Off
Elliptical filter	On

Geometry - Common

Slice group	1
Slices	1
Dist. factor	20 %
Position	L0.0 P47.8 F62.3 mm
Orientation	Sagittal
Phase enc. dir.	A >> P
Slice group	2
Slices	1
Dist. factor	20 %
Position	L0.0 P47.8 F62.3 mm
Orientation	Transversal
Phase enc. dir.	A >> P
Slice group	3
Slices	1
Dist. factor	20 %
Position	L0.0 P47.8 F62.3 mm
Orientation	Coronal
Phase enc. dir.	R >> L
FoV read	250 mm
FoV phase	100.0 %
Slice thickness	7.0 mm
TR	7.5 ms
Multi-slice mode	Sequential
Series	Interleaved
Concatenations	3

Geometry - AutoAlign

Slice group	1
Position	L0.0 P47.8 F62.3 mm
Orientation	Sagittal
Phase enc. dir.	A >> P
Slice group	2
Position	L0.0 P47.8 F62.3 mm

Boardman JP, *et al. BMJ Open* 2020; 10:e035854. doi: 10.1136/bmjopen-2019-035854

SIEMENS MAGNETOM Prisma

Geometry - AutoAlign

Orientation	Transversal
Phase enc. dir.	A >> P
Slice group	3
Position	L0.0 P47.8 F62.3 mm
Orientation	Coronal
Phase enc. dir.	R >> L
AutoAlign	---
Initial Position	L0.0 P47.8 F62.3
L	0.0 mm
P	47.8 mm
F	62.3 mm
Initial Rotation	0.00 deg
Initial Orientation	Sagittal

Geometry - Saturation

Saturation mode	Standard
Fat suppr.	None
Water suppr.	None
Special sat.	None

Geometry - Tim Planning Suite

Set-n-Go Protocol	Off
Table position	H
Table position	0 mm
Inline Composing	Off

System - Miscellaneous

Positioning mode	REF
Table position	H
Table position	0 mm
MSMA	S - C - T
Sagittal	R >> L
Coronal	A >> P
Transversal	F >> H
Coil Combine Mode	Adaptive Combine
Save uncombined	Off
Matrix Optimization	Off
AutoAlign	---
Coil Select Mode	Default

System - Adjustments

B0 Shim mode	Tune up
B1 Shim mode	TrueForm
Adjust with body coil	Off
Confirm freq. adjustment	Off
Assume Dominant Fat	Off
Assume Silicone	Off
Adjustment Tolerance	Auto

System - Adjust Volume

Position	Isocenter
Orientation	Transversal
Rotation	0.00 deg
A >> P	263 mm
R >> L	350 mm
F >> H	350 mm
Reset	Off

System - pTx Volumes

B1 Shim mode	TrueForm
Excitation	Slice-sel.

System - Tx/Rx

Frequency 1H	123.244318 MHz
Correction factor	1
Gain	High
Img. Scale Cor.	1.000
Reset	Off
? Ref. amplitude 1H	0.000 V

Physio - Signal1

1st Signal/Mode	None
TR	7.5 ms
Concatenations	3
Segments	1

Physio - Cardiac

Tagging	None
Magn. preparation	None
Fat suppr.	None
Dark blood	Off
FoV read	250 mm
FoV phase	100.0 %
Phase resolution	91 %

Physio - PACE

Resp. control	Off
Concatenations	3

Inline - Common

Subtract	Off
Measurements	1
StdDev	Off
Liver registration	Off
Save original images	On

Inline - MIP

MIP-Sag	Off
MIP-Cor	Off
MIP-Tra	Off
MIP-Time	Off
Save original images	On

Inline - Soft Tissue

Wash - In	Off
Wash - Out	Off
TTP	Off
PEI	Off
MIP - time	Off
Measurements	1

Inline - Composing

Inline Composing	Off
Distortion Corr.	Off

Inline - MapIt

Save original images	On
MapIt	None
Flip angle	20 deg
Measurements	1
Contrasts	1
TR	7.5 ms
TE	3.69 ms

Sequence - Part 1

Introduction	On

SIEMENS MAGNETOM Prisma

Sequence - Part 1

Dimension	2D
Phase stabilisation	Off
Asymmetric echo	Allowed
Contrasts	1
Flow comp.	No
Multi-slice mode	Sequential
Bandwidth	320 Hz/Px

Sequence - Part 2

Segments	1
Acoustic noise reduction	None
RF pulse type	Fast
Gradient mode	Fast
Excitation	Slice-sel.
RF spoiling	On

Sequence - Assistant

Mode	Off
Allowed delay	0 s

SIEMENS MAGNETOM Prisma

\\Study Protocols\BRAIN\Neonates\Theirworld - E161723\t2_haste_localiser

TA: 6.0 s PM: REF Voxel size: 0.7×0.7×4.0 mmPAT: 2 Rel. SNR: 1.00 : h

Properties

Prio recon	Off
Load images to viewer	On
Inline movie	Off
Auto store images	On
Load images to stamp segments	On
Load images to graphic segments	On
Auto open inline display	Off
Auto close inline display	Off
Start measurement without further preparation	Off
Wait for user to start	Off
Start measurements	Single measurement

Routine

Slice group	1
Slices	1
Dist. factor	30 %
Position	Isocenter
Orientation	Sagittal
Phase enc. dir.	A >> P
Slice group	2
Slices	1
Dist. factor	30 %
Position	L0.0 P0.0 H5.2 mm
Orientation	Transversal
Phase enc. dir.	R >> L
Slice group	3
Slices	1
Dist. factor	30 %
Position	L0.0 P0.0 H10.4 mm
Orientation	Coronal
Phase enc. dir.	R >> L
AutoAlign	---
Phase oversampling	0 %
FoV read	220 mm
FoV phase	100.0 %
Slice thickness	4.0 mm
TR	1500.0 ms
TE	94 ms
Averages	1
Concatenations	1
Filter	Prescan Normalize, Elliptical filter
Coil elements	HE1-4

Contrast - Common

TR	1500.0 ms
TE	94 ms
MTC	Off
Magn. preparation	None
Flip angle	150 deg
Fat suppr.	None
Water suppr.	None
Restore magn.	Off

Contrast - Dynamic

Averages	1
Averaging mode	Long term
Reconstruction	Magnitude
Measurements	1
Multiple series	Each measurement

Resolution - Common

FoV read	220 mm
FoV phase	100.0 %
Slice thickness	4.0 mm
Base resolution	320
Phase resolution	80 %
Phase partial Fourier	4/8
Interpolation	Off

Resolution - iPAT

PAT mode	GRAPPA
Accel. factor PE	2
Ref. lines PE	24
Reference scan mode	Integrated

Resolution - Filter Image

Image Filter	Off
Distortion Corr.	Off
Prescan Normalize	On
Unfiltered images	Off
Normalize	Off
B1 filter	Off

Resolution - Filter Rawdata

Raw filter	Off
Elliptical filter	On

Geometry - Common

Slice group	1
Slices	1
Dist. factor	30 %
Position	Isocenter
Orientation	Sagittal
Phase enc. dir.	A >> P
Slice group	2
Slices	1
Dist. factor	30 %
Position	L0.0 P0.0 H5.2 mm
Orientation	Transversal
Phase enc. dir.	R >> L
Slice group	3
Slices	1
Dist. factor	30 %
Position	L0.0 P0.0 H10.4 mm
Orientation	Coronal
Phase enc. dir.	R >> L
FoV read	220 mm
FoV phase	100.0 %
Slice thickness	4.0 mm
TR	1500.0 ms
Multi-slice mode	Single shot
Series	Interleaved
Concatenations	1

Geometry - AutoAlign

Slice group	1
Position	Isocenter
Orientation	Sagittal
Phase enc. dir.	A >> P
Slice group	2
Position	L0.0 P0.0 H5.2 mm

SIEMENS MAGNETOM Prisma

Geometry - AutoAlign

Orientation	Transversal
Phase enc. dir.	R >> L
Slice group	3
Position	L0.0 P0.0 H10.4 mm
Orientation	Coronal
Phase enc. dir.	R >> L
AutoAlign	---
Initial Position	Isocenter
L	0.0 mm
P	0.0 mm
H	0.0 mm
Initial Rotation	0.00 deg
Initial Orientation	Sagittal

Geometry - Saturation

Fat suppr.	None
Water suppr.	None
Restore magn.	Off
Special sat.	None

Geometry - Navigator

Geometry - Tim Planning Suite

Set-n-Go Protocol	Off
Table position	H
Table position	0 mm
Inline Composing	Off

System - Miscellaneous

Positioning mode	REF
Table position	H
Table position	0 mm
MSMA	S - C - T
Sagittal	R >> L
Coronal	A >> P
Transversal	F >> H
Coil Combine Mode	Adaptive Combine
Save uncombined	Off
Matrix Optimization	Off
AutoAlign	---
Coil Select Mode	On - AutoCoilSelect

System - Adjustments

B0 Shim mode	Tune up
B1 Shim mode	TrueForm
Adjust with body coil	Off
Confirm freq. adjustment	Off
Assume Dominant Fat	Off
Assume Silicone	Off
Adjustment Tolerance	Auto

System - Adjust Volume

Position	Isocenter
Orientation	Transversal
Rotation	0.00 deg
A >> P	263 mm
R >> L	350 mm
F >> H	350 mm
Reset	Off

System - pTx Volumes

B1 Shim mode	TrueForm

System - Tx/Rx

Frequency 1H	123.244318 MHz
Correction factor	1
Gain	High
Img. Scale Cor.	1.000
Reset	Off
? Ref. amplitude 1H	0.000 V

Physio - Signal1

1st Signal/Mode	None
TR	1500.0 ms
Concatenations	1

Physio - Cardiac

Magn. preparation	None
Fat suppr.	None
Dark blood	Off
FoV read	220 mm
FoV phase	100.0 %
Phase resolution	80 %

Physio - PACE

Resp. control	Off
Concatenations	1

Inline - Common

Subtract	Off
Measurements	1
StdDev	Off
Save original images	On

Inline - MIP

MIP-Sag	Off
MIP-Cor	Off
MIP-Tra	Off
MIP-Time	Off
Save original images	On

Inline - Composing

Inline Composing	Off
Distortion Corr.	Off

Sequence - Part 1

Introduction	On
Dimension	2D
Contrasts	1
Flow comp.	No
Multi-slice mode	Single shot
Echo spacing	7.22 ms
Bandwidth	601 Hz/Px

Sequence - Part 2

RF pulse type	Normal
Gradient mode	Whisper
Hyperecho	Off
Turbo factor	256

Sequence - Assistant

Mode	Min flip angle
Min flip angle	130 deg
Allowed delay	60 s

Boardman JP, *et al. BMJ Open* 2020; 10:e035854. doi: 10.1136/bmjopen-2019-035854

SIEMENS MAGNETOM Prisma

\\Study Protocols\BRAIN\Neonates\Theirworld - E161723\t2_blade_v3
TA: 2:29 PM: REF Voxel size: 0.7×0.7×3.0 mmPAT: 2 Rel. SNR: 1.00 : qtseBR_rr

Properties

Prio recon	Off
Load images to viewer	On
Inline movie	Off
Auto store images	On
Load images to stamp segments	On
Load images to graphic segments	Off
Auto open inline display	Off
Auto close inline display	Off
Start measurement without further preparation	Off
Wait for user to start	Off
Start measurements	Single measurement

Routine

Slice group	1
Slices	40
Dist. factor	0 %
Position	R1.2 P40.0 H50.2 mm
Orientation	Transversal
Phase enc. dir.	A >> P
AutoAlign	---
Phase oversampling	0.0 %
FoV read	220 mm
FoV phase	100.0 %
Slice thickness	3.0 mm
TR	4100.0 ms
TE	207 ms
Averages	1
Concatenations	4
Filter	Prescan Normalize
Coil elements	PeH;PeN

Contrast - Common

TR	4100.0 ms
TE	207 ms
TD	0.0 ms
MTC	Off
Magn. preparation	None
Flip angle	90 deg
Fat suppr.	None
Water suppr.	None
Restore magn.	On

Contrast - Dynamic

Averages	1
Averaging mode	Short term
Reconstruction	Magnitude
Measurements	1
Multiple series	Each measurement

Resolution - Common

FoV read	220 mm
FoV phase	100.0 %
Slice thickness	3.0 mm
Base resolution	320
BLADE coverage	100.0 %
Trajectory	BLADE
Interpolation	Off

Resolution - iPAT

PAT mode	GRAPPA
Accel. factor PE	2
Ref. lines PE	8
Reference scan mode	Integrated

Resolution - Filter Image

Image Filter	Off
Distortion Corr.	Off
Prescan Normalize	On
Unfiltered images	Off
Normalize	Off
B1 filter	Off

Resolution - Filter Rawdata

Raw filter	Off
Elliptical filter	Off

Geometry - Common

Slice group	1
Slices	40
Dist. factor	0 %
Position	R1.2 P40.0 H50.2 mm
Orientation	Transversal
Phase enc. dir.	A >> P
FoV read	220 mm
FoV phase	100.0 %
Slice thickness	3.0 mm
TR	4100.0 ms
Multi-slice mode	Interleaved
Series	Interleaved
Concatenations	4

Geometry - AutoAlign

Slice group	1
Position	R1.2 P40.0 H50.2 mm
Orientation	Transversal
Phase enc. dir.	A >> P
AutoAlign	---
Initial Position	R1.2 P40.0 H50.2
R	1.2 mm
P	40.0 mm
H	50.2 mm
Initial Rotation	0.00 deg
Initial Orientation	Transversal

Geometry - Saturation

Fat suppr.	None
Water suppr.	None
Restore magn.	On
Special sat.	None

Geometry - Navigator

Geometry - Tim Planning Suite

Set-n-Go Protocol	Off
Table position	H
Table position	0 mm
Inline Composing	Off

Boardman JP, *et al. BMJ Open* 2020; 10:e035854. doi: 10.1136/bmjopen-2019-035854

SIEMENS MAGNETOM Prisma

System - Miscellaneous

Positioning mode	REF
Table position	H
Table position	0 mm
MSMA	S - C - T
Sagittal	R >> L
Coronal	A >> P
Transversal	F >> H
Coil Combine Mode	Adaptive Combine
Save uncombined	Off
Matrix Optimization	Off
AutoAlign	---
Coil Select Mode	On - AutoCoilSelect

System - Adjustments

B0 Shim mode	Tune up
B1 Shim mode	TrueForm
Adjust with body coil	Off
Confirm freq. adjustment	Off
Assume Dominant Fat	Off
Assume Silicone	Off
Adjustment Tolerance	Auto

System - Adjust Volume

Position	Isocenter
Orientation	Transversal
Rotation	0.00 deg
A >> P	263 mm
R >> L	350 mm
F >> H	350 mm
Reset	Off

System - pTx Volumes

B1 Shim mode	TrueForm

System - Tx/Rx

Frequency 1H	123.244318 MHz
Correction factor	1
Gain	High
Img. Scale Cor.	1.000
Reset	Off
? Ref. amplitude 1H	0.000 V

Physio - Signal1

1st Signal/Mode	None
TR	4100.0 ms
Concatenations	4

Physio - Cardiac

Magn. preparation	None
Fat suppr.	None
Dark blood	Off
FoV read	220 mm
FoV phase	100.0 %
BLADE coverage	100.0 %
Trajectory	BLADE

Physio - PACE

Resp. control	Off
Concatenations	4

Inline - Common

Subtract	Off
Measurements	1

Inline - Common

StdDev	Off
Save original images	On

Inline - MIP

MIP-Sag	Off
MIP-Cor	Off
MIP-Tra	Off
MIP-Time	Off
Save original images	On

Inline - Composing

Inline Composing	Off
Distortion Corr.	Off

Sequence - Part 1

Introduction	On
Dimension	2D
Compensate T2 decay	Off
Contrasts	1
Flow comp.	Read
Multi-slice mode	Interleaved
Free echo spacing	Off
Echo spacing	10.9 ms
Bandwidth	363 Hz/Px

Sequence - Part 2

Define	Turbo factor
Echo trains per slice	8
Phase correction	Automatic
Acoustic noise reduction	Active
RF pulse type	Low SAR
Gradient mode	Fast
Hyperecho	On
WARP	Off
Motion correction	On
Red. EC sensitivity	Off
Turbo factor	36

Sequence - Assistant

Mode	Off
Allowed delay	30 s

Boardman JP, *et al. BMJ Open* 2020; 10:e035854. doi: 10.1136/bmjopen-2019-035854

SIEMENS MAGNETOM Prisma

\\Study Protocols\BRAIN\Neonates\Theirworld - E161723\t2_space_sag_p4_iso_v2x
TA: 2:13 PM: REF Voxel size: 1.0×1.0×1.0 mmPAT: 4 Rel. SNR: 1.00 : spcR

Properties

Prio recon	Off
Load images to viewer	On
Inline movie	Off
Auto store images	On
Load images to stamp segments	Off
Load images to graphic segments	Off
Auto open inline display	Off
Auto close inline display	Off
Start measurement without further preparation	Off
Wait for user to start	Off
Start measurements	Single measurement

Routine

Slab group	1
Slabs	1
Position	R1.2 P36.9 H0.0 mm
Orientation	Sagittal
Phase enc. dir.	A >> P
AutoAlign	---
Phase oversampling	0 %
Slice oversampling	0.0 %
Slices per slab	160
FoV read	128 mm
FoV phase	150.0 %
Slice thickness	1.00 mm
TR	3200 ms
TE	409 ms
Averages	1.4
Concatenations	1
Filter	Raw filter, Prescan Normalize
Coil elements	PeH;PeN

Contrast - Common

TR	3200 ms
TE	409 ms
MTC	Off
Magn. preparation	None
Fat suppr.	Fat sat.
Fat sat. mode	Strong
Blood suppr.	Off
Restore magn.	On

Contrast - Dynamic

Averages	1.4
Reconstruction	Magnitude
Measurements	1
Multiple series	Each measurement

Resolution - Common

FoV read	128 mm
FoV phase	150.0 %
Slice thickness	1.00 mm
Base resolution	128
Phase resolution	100 %
Slice resolution	100 %
Phase partial Fourier	Allowed
Slice partial Fourier	Off
Interpolation	Off

Resolution - iPAT

PAT mode	GRAPPA
Accel. factor PE	2
Ref. lines PE	24
Accel. factor 3D	2
Ref. lines 3D	24
Reference scan mode	Integrated

Resolution - Filter Image

Image Filter	Off
Distortion Corr.	Off
Prescan Normalize	On
Unfiltered images	Off
Normalize	Off
B1 filter	Off

Resolution - Filter Rawdata

Raw filter	On
Elliptical filter	Off

Geometry - Common

Slab group	1
Slabs	1
Position	R1.2 P36.9 H0.0 mm
Orientation	Sagittal
Phase enc. dir.	A >> P
Slice oversampling	0.0 %
Slices per slab	160
FoV read	128 mm
FoV phase	150.0 %
Slice thickness	1.00 mm
TR	3200 ms
Series	Interleaved
Concatenations	1

Geometry - AutoAlign

Slab group	1
Position	R1.2 P36.9 H0.0 mm
Orientation	Sagittal
Phase enc. dir.	A >> P
AutoAlign	---
Initial Position	R1.2 P36.9 H0.0
R	1.2 mm
P	36.9 mm
H	0.0 mm
Initial Rotation	0.00 deg
Initial Orientation	Sagittal

Geometry - Saturation

Fat suppr.	Fat sat.
Fat sat. mode	Strong
Restore magn.	On
Special sat.	None

Geometry - Navigator

Geometry - Tim Planning Suite

Set-n-Go Protocol	Off
Table position	H
Table position	0 mm
Inline Composing	Off

SIEMENS MAGNETOM Prisma

System - Miscellaneous

Positioning mode	REF
Table position	H
Table position	0 mm
MSMA	S - C - T
Sagittal	R >> L
Coronal	A >> P
Transversal	F >> H
Coil Combine Mode	Adaptive Combine
Save uncombined	Off
Matrix Optimization	Off
AutoAlign	---
Coil Select Mode	On - AutoCoilSelect

System - Adjustments

B0 Shim mode	Standard
B1 Shim mode	TrueForm
Adjust with body coil	Off
Confirm freq. adjustment	Off
Assume Dominant Fat	Off
Assume Silicone	Off
Adjustment Tolerance	Auto

System - Adjust Volume

Position	R1.2 P36.9 H0.0 mm
Orientation	Sagittal
Rotation	90.00 deg
F >> H	128 mm
A >> P	192 mm
R >> L	160 mm
Reset	Off

System - pTx Volumes

B1 Shim mode	TrueForm
Excitation	Non-sel.

System - Tx/Rx

Frequency 1H	123.244318 MHz
Correction factor	1
Gain	High
Img. Scale Cor.	3.000
Reset	Off
? Ref. amplitude 1H	0.000 V

Physio - Signal1

1st Signal/Mode	None
Trigger delay	0 ms
TR	3200 ms
Concatenations	1

Physio - Cardiac

Magn. preparation	None
Fat suppr.	Fat sat.
Dark blood	Off
FoV read	128 mm
FoV phase	150.0 %
Phase resolution	100 %

Physio - PACE

Resp. control	Off
Concatenations	1

Inline - Common

Subtract	Off

Inline - Common

Measurements	1
StdDev	Off
Save original images	On

Inline - MIP

MIP-Sag	Off
MIP-Cor	Off
MIP-Tra	Off
MIP-Time	Off
Save original images	On

Inline - Composing

Inline Composing	Off
Distortion Corr.	Off

Sequence - Part 1

Introduction	On
Dimension	3D
Elliptical scanning	Off
Reordering	Linear
Flow comp.	No
Echo spacing	4.4 ms
Adiabatic-mode	Off
Bandwidth	592 Hz/Px

Sequence - Part 2

Echo train duration	1034 ms
RF pulse type	Low SAR
Gradient mode	Whisper
Excitation	Non-sel.
Flip angle mode	T2 var
Turbo factor	282

Sequence - Assistant

Allowed delay	30 s

Boardman JP, *et al. BMJ Open* 2020; 10:e035854. doi: 10.1136/bmjopen-2019-035854

SIEMENS MAGNETOM Prisma

\\Study Protocols\BRAIN\Neonates\Theirworld - E161723\DTI_Neonate_v6b_dummy

TA: 0:28 PM: FIX Voxel size: 2.0×2.0×2.0 mmPAT: 4 Rel. SNR: 1.00 : epse

Properties

Prio recon	Off
Load images to viewer	On
Inline movie	Off
Auto store images	On
Load images to stamp segments	Off
Load images to graphic segments	Off
Auto open inline display	Off
Auto close inline display	Off
Start measurement without further preparation	Off
Wait for user to start	Off
Start measurements	Single measurement

Routine

Slice group	1
Slices	58
Dist. factor	0 %
Position	R1.2 P39.7 H47.8 mm
Orientation	Transversal
Phase enc. dir.	R >> L
AutoAlign	---
Phase oversampling	0 %
FoV read	256 mm
FoV phase	100.0 %
Slice thickness	2.0 mm
TR	3500 ms
TE	78.0 ms
Concatenations	1
Filter	Raw filter, Prescan Normalize
Coil elements	PeH;PeN

Contrast - Common

TR	3500 ms
TE	78.0 ms
MTC	Off
Magn. preparation	None
Fat suppr.	Fat sat.
Fat sat. mode	Strong

Contrast - Dynamic

Averaging mode	Long term
Reconstruction	Magnitude
Measurements	1
Delay in TR	0 ms
Multiple series	Off

Resolution - Common

FoV read	256 mm
FoV phase	100.0 %
Slice thickness	2.0 mm
Base resolution	128
Phase resolution	100 %
Phase partial Fourier	7/8
Interpolation	Off

Resolution - iPAT

Accel. mode	Slice accel.
Accel. factor PE	2
Ref. lines PE	40

Resolution - iPAT

Accel. factor slice	2
Reference scan mode	EPI/separate

Resolution - Filter Image

Distortion Corr.	Off
Prescan Normalize	On
Dynamic Field Corr.	Off

Resolution - Filter Rawdata

Raw filter	On
Elliptical filter	Off

Geometry - Common

Slice group	1
Slices	58
Dist. factor	0 %
Position	R1.2 P39.7 H47.8 mm
Orientation	Transversal
Phase enc. dir.	R >> L
FoV read	256 mm
FoV phase	100.0 %
Slice thickness	2.0 mm
TR	3500 ms
Multi-slice mode	Interleaved
Series	Interleaved
Concatenations	1

Geometry - AutoAlign

Slice group	1
Position	R1.2 P39.7 H47.8 mm
Orientation	Transversal
Phase enc. dir.	R >> L
AutoAlign	---
Initial Position	R1.2 P39.7 H47.8
R	1.2 mm
P	39.7 mm
H	47.8 mm
Initial Rotation	90.00 deg
Initial Orientation	Transversal

Geometry - Saturation

Fat suppr.	Fat sat.
Fat sat. mode	Strong
Special sat.	None

Geometry - Navigator

Geometry - Tim Planning Suite

Set-n-Go Protocol	Off
Table position	H
Table position	0 mm
Inline Composing	Off

System - Miscellaneous

Positioning mode	FIX
Table position	H
Table position	0 mm
MSMA	S - C - T
Sagittal	R >> L

Boardman JP, *et al. BMJ Open* 2020; 10:e035854. doi: 10.1136/bmjopen-2019-035854

SIEMENS MAGNETOM Prisma

System - Miscellaneous

Coronal	A >> P
Transversal	F >> H
Coil Combine Mode	Adaptive Combine
Matrix Optimization	Performance
AutoAlign	---
Coil Select Mode	On - AutoCoilSelect

System - Adjustments

B0 Shim mode	Standard
B1 Shim mode	TrueForm
Adjust with body coil	Off
Confirm freq. adjustment	Off
Assume Dominant Fat	Off
Assume Silicone	Off
Adjustment Tolerance	Auto

System - Adjust Volume

Position	R1.2 P39.7 H47.8 mm
Orientation	Transversal
Rotation	90.00 deg
R >> L	256 mm
A >> P	256 mm
F >> H	116 mm
Reset	Off

System - pTx Volumes

B1 Shim mode	TrueForm
Excitation	Standard

System - Tx/Rx

Frequency 1H	123.244318 MHz
Correction factor	1
Gain	High
Img. Scale Cor.	1.000
Reset	Off
? Ref. amplitude 1H	0.000 V

Physio - Signal1

1st Signal/Mode	None
TR	3500 ms
Concatenations	1

Physio - PACE

Resp. control	Off
Concatenations	1

Diff - Neuro

Diffusion mode	Free
Diff. directions	71
Diffusion Scheme	Monopolar
Diff. weightings	1
b-value	0 s/mm²
b-value	3
Diff. weighted images	On
Trace weighted images	Off
ADC maps	Off
FA maps	Off
Mosaic	Off
Tensor	Off
Noise level	40

Diff - Body

Diffusion mode	Free
Diff. directions	71
Diffusion Scheme	Monopolar
Diff. weightings	1
b-value	0 s/mm²
b-value	3
Diff. weighted images	On
Trace weighted images	Off
ADC maps	Off
Exponential ADC Maps	Off
FA maps	Off
Invert Gray Scale	Off
Calculated Image	Off
b-Value >=	0 s/mm²
Noise level	40

Diff - Composing

Inline Composing	Off
Distortion Corr.	Off

Sequence - Part 1

Introduction	Off
Optimization	None
Multi-slice mode	Interleaved
Free echo spacing	Off
Echo spacing	0.78 ms
Bandwidth	1446 Hz/Px

Sequence - Part 2

EPI factor	128
RF pulse type	Low SAR
Gradient mode	Normal
Excitation	Standard

Sequence - pTX Pulses

Boardman JP, *et al. BMJ Open* 2020; 10:e035854. doi: 10.1136/bmjopen-2019-035854

SIEMENS MAGNETOM Prisma

\\Study Protocols\BRAIN\Neonates\Theirworld - E161723\DTI_Neonate_v6b_rev
TA: 0:28 PM: FIX Voxel size: 2.0×2.0×2.0 mmPAT: 4 Rel. SNR: 1.00 : epse

Properties

Prio recon	Off
Load images to viewer	On
Inline movie	Off
Auto store images	On
Load images to stamp segments	Off
Load images to graphic segments	Off
Auto open inline display	Off
Auto close inline display	Off
Start measurement without further preparation	Off
Wait for user to start	Off
Start measurements	Single measurement

Routine

Slice group	1
Slices	58
Dist. factor	0 %
Position	R1.2 P39.7 H47.8 mm
Orientation	Transversal
Phase enc. dir.	R >> L
AutoAlign	---
Phase oversampling	0 %
FoV read	256 mm
FoV phase	100.0 %
Slice thickness	2.0 mm
TR	3500 ms
TE	78.0 ms
Concatenations	1
Filter	Raw filter, Prescan Normalize
Coil elements	PeH;PeN

Contrast - Common

TR	3500 ms
TE	78.0 ms
MTC	Off
Magn. preparation	None
Fat suppr.	Fat sat.
Fat sat. mode	Strong

Contrast - Dynamic

Averaging mode	Long term
Reconstruction	Magnitude
Measurements	1
Delay in TR	0 ms
Multiple series	Off

Resolution - Common

FoV read	256 mm
FoV phase	100.0 %
Slice thickness	2.0 mm
Base resolution	128
Phase resolution	100 %
Phase partial Fourier	7/8
Interpolation	Off

Resolution - iPAT

Accel. mode	Slice accel.
Accel. factor PE	2
Ref. lines PE	40

Resolution - iPAT

Accel. factor slice	2
Reference scan mode	EPI/separate

Resolution - Filter Image

Distortion Corr.	Off
Prescan Normalize	On
Dynamic Field Corr.	Off

Resolution - Filter Rawdata

Raw filter	On
Elliptical filter	Off

Geometry - Common

Slice group	1
Slices	58
Dist. factor	0 %
Position	R1.2 P39.7 H47.8 mm
Orientation	Transversal
Phase enc. dir.	R >> L
FoV read	256 mm
FoV phase	100.0 %
Slice thickness	2.0 mm
TR	3500 ms
Multi-slice mode	Interleaved
Series	Interleaved
Concatenations	1

Geometry - AutoAlign

Slice group	1
Position	R1.2 P39.7 H47.8 mm
Orientation	Transversal
Phase enc. dir.	R >> L
AutoAlign	---
Initial Position	R1.2 P39.7 H47.8
R	1.2 mm
P	39.7 mm
H	47.8 mm
Initial Rotation	90.00 deg
Initial Orientation	Transversal

Geometry - Saturation

Fat suppr.	Fat sat.
Fat sat. mode	Strong
Special sat.	None

Geometry - Navigator

Geometry - Tim Planning Suite

Set-n-Go Protocol	Off
Table position	H
Table position	0 mm
Inline Composing	Off

System - Miscellaneous

Positioning mode	FIX
Table position	H
Table position	0 mm
MSMA	S - C - T
Sagittal	R >> L

SIEMENS MAGNETOM Prisma

System - Miscellaneous

Coronal	A >> P
Transversal	F >> H
Coil Combine Mode	Adaptive Combine
Matrix Optimization	Performance
AutoAlign	---
Coil Select Mode	On - AutoCoilSelect

System - Adjustments

B0 Shim mode	Standard
B1 Shim mode	TrueForm
Adjust with body coil	Off
Confirm freq. adjustment	Off
Assume Dominant Fat	Off
Assume Silicone	Off
Adjustment Tolerance	Auto

System - Adjust Volume

Position	R1.2 P39.7 H47.8 mm
Orientation	Transversal
Rotation	90.00 deg
R >> L	256 mm
A >> P	256 mm
F >> H	116 mm
Reset	Off

System - pTx Volumes

B1 Shim mode	TrueForm
Excitation	Standard

System - Tx/Rx

Frequency 1H	123.244318 MHz
Correction factor	1
Gain	High
Img. Scale Cor.	1.000
Reset	Off
? Ref. amplitude 1H	0.000 V

Physio - Signal1

1st Signal/Mode	None
TR	3500 ms
Concatenations	1

Physio - PACE

Resp. control	Off
Concatenations	1

Diff - Neuro

Diffusion mode	MDDW
Diff. directions	6
Diffusion Scheme	Monopolar
Diff. weightings	1
b-value	0 s/mm²
b-value	3
Diff. weighted images	On
Trace weighted images	Off
ADC maps	Off
FA maps	Off
Mosaic	Off
Tensor	Off
Noise level	40

Diff - Body

Diffusion mode	MDDW
Diff. directions	6
Diffusion Scheme	Monopolar
Diff. weightings	1
b-value	0 s/mm²
b-value	3
Diff. weighted images	On
Trace weighted images	Off
ADC maps	Off
Exponential ADC Maps	Off
FA maps	Off
Invert Gray Scale	Off
Calculated Image	Off
b-Value >=	0 s/mm²
Noise level	40

Diff - Composing

Inline Composing	Off
Distortion Corr.	Off

Sequence - Part 1

Introduction	Off
Optimization	None
Multi-slice mode	Interleaved
Free echo spacing	Off
Echo spacing	0.78 ms
Bandwidth	1446 Hz/Px

Sequence - Part 2

EPI factor	128
RF pulse type	Low SAR
Gradient mode	Normal
Excitation	Standard

Sequence - pTX Pulses

Boardman JP, *et al. BMJ Open* 2020; 10:e035854. doi: 10.1136/bmjopen-2019-035854

SIEMENS MAGNETOM Prisma

\\Study Protocols\BRAIN\Neonates\Theirworld - E161723\DTI_Neonate_v6b_pt1
TA: 4:29 PM: FIX Voxel size: 2.0×2.0×2.0 mmPAT: 4 Rel. SNR: 1.00 : epse

Properties

Prio recon	Off
Load images to viewer	On
Inline movie	Off
Auto store images	On
Load images to stamp segments	Off
Load images to graphic segments	Off
Auto open inline display	Off
Auto close inline display	Off
Start measurement without further preparation	Off
Wait for user to start	Off
Start measurements	Single measurement

Routine

Slice group	1
Slices	58
Dist. factor	0 %
Position	R1.2 P39.7 H47.8 mm
Orientation	Transversal
Phase enc. dir.	L >> R
AutoAlign	---
Phase oversampling	0 %
FoV read	256 mm
FoV phase	100.0 %
Slice thickness	2.0 mm
TR	3500 ms
TE	78.0 ms
Averages	1
Concatenations	1
Filter	Raw filter, Prescan Normalize
Coil elements	PeH;PeN

Contrast - Common

TR	3500 ms
TE	78.0 ms
MTC	Off
Magn. preparation	None
Fat suppr.	Fat sat.
Fat sat. mode	Strong

Contrast - Dynamic

Averages	1
Averaging mode	Long term
Reconstruction	Magnitude
Measurements	1
Delay in TR	0 ms
Multiple series	Off

Resolution - Common

FoV read	256 mm
FoV phase	100.0 %
Slice thickness	2.0 mm
Base resolution	128
Phase resolution	100 %
Phase partial Fourier	7/8
Interpolation	Off

Resolution - iPAT

Accel. mode	Slice accel.

Resolution - iPAT

Accel. factor PE	2
Ref. lines PE	40
Accel. factor slice	2
Reference scan mode	EPI/separate

Resolution - Filter Image

Distortion Corr.	Off
Prescan Normalize	On
Dynamic Field Corr.	Off

Resolution - Filter Rawdata

Raw filter	On
Elliptical filter	Off

Geometry - Common

Slice group	1
Slices	58
Dist. factor	0 %
Position	R1.2 P39.7 H47.8 mm
Orientation	Transversal
Phase enc. dir.	L >> R
FoV read	256 mm
FoV phase	100.0 %
Slice thickness	2.0 mm
TR	3500 ms
Multi-slice mode	Interleaved
Series	Interleaved
Concatenations	1

Geometry - AutoAlign

Slice group	1
Position	R1.2 P39.7 H47.8 mm
Orientation	Transversal
Phase enc. dir.	L >> R
AutoAlign	---
Initial Position	R1.2 P39.7 H47.8
R	1.2 mm
P	39.7 mm
H	47.8 mm
Initial Rotation	-90.00 deg
Initial Orientation	Transversal

Geometry - Saturation

Fat suppr.	Fat sat.
Fat sat. mode	Strong
Special sat.	None

Geometry - Navigator

Geometry - Tim Planning Suite

Set-n-Go Protocol	Off
Table position	H
Table position	0 mm
Inline Composing	Off

System - Miscellaneous

Positioning mode	FIX
Table position	H
Table position	0 mm

Boardman JP, *et al. BMJ Open* 2020; 10:e035854. doi: 10.1136/bmjopen-2019-035854

SIEMENS MAGNETOM Prisma

System - Miscellaneous

MSMA	S - C - T
Sagittal	R >> L
Coronal	A >> P
Transversal	F >> H
Coil Combine Mode	Adaptive Combine
Matrix Optimization	Performance
AutoAlign	---
Coil Select Mode	On - AutoCoilSelect

System - Adjustments

B0 Shim mode	Standard
B1 Shim mode	TrueForm
Adjust with body coil	Off
Confirm freq. adjustment	Off
Assume Dominant Fat	Off
Assume Silicone	Off
Adjustment Tolerance	Auto

System - Adjust Volume

Position	R1.2 P39.7 H47.8 mm
Orientation	Transversal
Rotation	-90.00 deg
R >> L	256 mm
A >> P	256 mm
F >> H	116 mm
Reset	Off

System - pTx Volumes

B1 Shim mode	TrueForm
Excitation	Standard

System - Tx/Rx

Frequency 1H	123.244318 MHz
Correction factor	1
Gain	High
Img. Scale Cor.	1.000
Reset	Off
? Ref. amplitude 1H	0.000 V

Physio - Signal1

1st Signal/Mode	None
TR	3500 ms
Concatenations	1

Physio - PACE

Resp. control	Off
Concatenations	1

Diff - Neuro

Diffusion mode	Free
Diff. directions	71
Diffusion Scheme	Monopolar
Diff. weightings	2
b-value 1	0 s/mm²
b-value 2	750 s/mm²
b-value 1	1
b-value 2	1
Diff. weighted images	On
Trace weighted images	Off
ADC maps	Off
FA maps	Off
Mosaic	On
Tensor	Off

Diff - Neuro

Noise level	40

Diff - Body

Diffusion mode	Free
Diff. directions	71
Diffusion Scheme	Monopolar
Diff. weightings	2
b-value 1	0 s/mm²
b-value 2	750 s/mm²
b-value 1	1
b-value 2	1
Diff. weighted images	On
Trace weighted images	Off
ADC maps	Off
Exponential ADC Maps	Off
FA maps	Off
Invert Gray Scale	Off
Calculated Image	Off
b-Value >=	0 s/mm²
Noise level	40

Diff - Composing

Inline Composing	Off
Distortion Corr.	Off

Sequence - Part 1

Introduction	Off
Optimization	None
Multi-slice mode	Interleaved
Free echo spacing	Off
Echo spacing	0.78 ms
Bandwidth	1446 Hz/Px

Sequence - Part 2

EPI factor	128
RF pulse type	Low SAR
Gradient mode	Normal
Excitation	Standard

Sequence - pTX Pulses

Boardman JP, *et al. BMJ Open* 2020; 10:e035854. doi: 10.1136/bmjopen-2019-035854

SIEMENS MAGNETOM Prisma

**\\Study Protocols\BRAIN\Neonates\Theirworld - E161723\DTI_Neonate_v6b_pt2**
TA: 5:01 PM: FIX Voxel size: 2.0×2.0×2.0 mmPAT: 4 Rel. SNR: 1.00 : epse

Properties

Prio recon	Off
Load images to viewer	On
Inline movie	Off
Auto store images	On
Load images to stamp segments	Off
Load images to graphic segments	Off
Auto open inline display	Off
Auto close inline display	Off
Start measurement without further preparation	Off
Wait for user to start	Off
Start measurements	Single measurement

Routine

Slice group	1
Slices	58
Dist. factor	0 %
Position	R1.2 P39.7 H47.8 mm
Orientation	Transversal
Phase enc. dir.	L >> R
AutoAlign	---
Phase oversampling	0 %
FoV read	256 mm
FoV phase	100.0 %
Slice thickness	2.0 mm
TR	3500 ms
TE	78.0 ms
Averages	1
Concatenations	1
Filter	Raw filter, Prescan Normalize
Coil elements	PeH;PeN

Contrast - Common

TR	3500 ms
TE	78.0 ms
MTC	Off
Magn. preparation	None
Fat suppr.	Fat sat.
Fat sat. mode	Strong

Contrast - Dynamic

Averages	1
Averaging mode	Long term
Reconstruction	Magnitude
Measurements	1
Delay in TR	0 ms
Multiple series	Off

Resolution - Common

FoV read	256 mm
FoV phase	100.0 %
Slice thickness	2.0 mm
Base resolution	128
Phase resolution	100 %
Phase partial Fourier	7/8
Interpolation	Off

Resolution - iPAT

Accel. mode	Slice accel.

Resolution - iPAT

Accel. factor PE	2
Ref. lines PE	40
Accel. factor slice	2
Reference scan mode	EPI/separate

Resolution - Filter Image

Distortion Corr.	Off
Prescan Normalize	On
Dynamic Field Corr.	Off

Resolution - Filter Rawdata

Raw filter	On
Elliptical filter	Off

Geometry - Common

Slice group	1
Slices	58
Dist. factor	0 %
Position	R1.2 P39.7 H47.8 mm
Orientation	Transversal
Phase enc. dir.	L >> R
FoV read	256 mm
FoV phase	100.0 %
Slice thickness	2.0 mm
TR	3500 ms
Multi-slice mode	Interleaved
Series	Interleaved
Concatenations	1

Geometry - AutoAlign

Slice group	1
Position	R1.2 P39.7 H47.8 mm
Orientation	Transversal
Phase enc. dir.	L >> R
AutoAlign	---
Initial Position	R1.2 P39.7 H47.8
R	1.2 mm
P	39.7 mm
H	47.8 mm
Initial Rotation	-90.00 deg
Initial Orientation	Transversal

Geometry - Saturation

Fat suppr.	Fat sat.
Fat sat. mode	Strong
Special sat.	None

Geometry - Navigator

Geometry - Tim Planning Suite

Set-n-Go Protocol	Off
Table position	H
Table position	0 mm
Inline Composing	Off

System - Miscellaneous

Positioning mode	FIX
Table position	H
Table position	0 mm

Boardman JP, *et al. BMJ Open* 2020; 10:e035854. doi: 10.1136/bmjopen-2019-035854

SIEMENS MAGNETOM Prisma

System - Miscellaneous

MSMA	S - C - T
Sagittal	R >> L
Coronal	A >> P
Transversal	F >> H
Coil Combine Mode	Adaptive Combine
Matrix Optimization	Performance
AutoAlign	---
Coil Select Mode	On - AutoCoilSelect

System - Adjustments

B0 Shim mode	Standard
B1 Shim mode	TrueForm
Adjust with body coil	Off
Confirm freq. adjustment	Off
Assume Dominant Fat	Off
Assume Silicone	Off
Adjustment Tolerance	Auto

System - Adjust Volume

Position	R1.2 P39.7 H47.8 mm
Orientation	Transversal
Rotation	-90.00 deg
R >> L	256 mm
A >> P	256 mm
F >> H	116 mm
Reset	Off

System - pTx Volumes

B1 Shim mode	TrueForm
Excitation	Standard

System - Tx/Rx

Frequency 1H	123.244318 MHz
Correction factor	1
Gain	High
Img. Scale Cor.	1.000
Reset	Off
? Ref. amplitude 1H	0.000 V

Physio - Signal1

1st Signal/Mode	None
TR	3500 ms
Concatenations	1

Physio - PACE

Resp. control	Off
Concatenations	1

Diff - Neuro

Diffusion mode	Free
Diff. directions	80
Diffusion Scheme	Monopolar
Diff. weightings	2
b-value 1	0 s/mm²
b-value 2	2500 s/mm²
b-value 1	1
b-value 2	1
Diff. weighted images	On
Trace weighted images	Off
ADC maps	Off
FA maps	Off
Mosaic	On
Tensor	Off

Diff - Neuro

Noise level	40

Diff - Body

Diffusion mode	Free
Diff. directions	80
Diffusion Scheme	Monopolar
Diff. weightings	2
b-value 1	0 s/mm²
b-value 2	2500 s/mm²
b-value 1	1
b-value 2	1
Diff. weighted images	On
Trace weighted images	Off
ADC maps	Off
Exponential ADC Maps	Off
FA maps	Off
Invert Gray Scale	Off
Calculated Image	Off
b-Value >=	0 s/mm²
Noise level	40

Diff - Composing

Inline Composing	Off
Distortion Corr.	Off

Sequence - Part 1

Introduction	Off
Optimization	None
Multi-slice mode	Interleaved
Free echo spacing	Off
Echo spacing	0.78 ms
Bandwidth	1446 Hz/Px

Sequence - Part 2

EPI factor	128
RF pulse type	Low SAR
Gradient mode	Normal
Excitation	Standard

Sequence - pTX Pulses

Boardman JP, *et al. BMJ Open* 2020; 10:e035854. doi: 10.1136/bmjopen-2019-035854

SIEMENS MAGNETOM Prisma

\\Study Protocols\BRAIN\Neonates\Theirworld - E161723\MPRAGE-v4
TA: 3:09 PM: FIX Voxel size: 1.0×1.0×1.0 mmPAT: 2 Rel. SNR: 1.00 : tfl

Properties

Prio recon	Off
Load images to viewer	On
Inline movie	Off
Auto store images	On
Load images to stamp segments	On
Load images to graphic segments	Off
Auto open inline display	Off
Auto close inline display	Off
Start measurement without further preparation	Off
Wait for user to start	Off
Start measurements	Single measurement

Routine

Slab group	1
Slabs	1
Dist. factor	50 %
Position	R1.1 P38.9 F20.7 mm
Orientation	Sagittal
Phase enc. dir.	A >> P
AutoAlign	---
Phase oversampling	20 %
Slice oversampling	0.0 %
Slices per slab	160
FoV read	160 mm
FoV phase	100.0 %
Slice thickness	1.00 mm
TR	1970.0 ms
TE	4.69 ms
Averages	1
Concatenations	1
Filter	Prescan Normalize
Coil elements	PeH;PeN;SP1

Contrast - Common

TR	1970.0 ms
TE	4.69 ms
Magn. preparation	Non-sel. IR
TI	1100 ms
Flip angle	9 deg
Fat suppr.	None
Water suppr.	None

Contrast - Dynamic

Averages	1
Averaging mode	Long term
Reconstruction	Magnitude
Measurements	1
Multiple series	Each measurement

Resolution - Common

FoV read	160 mm
FoV phase	100.0 %
Slice thickness	1.00 mm
Base resolution	160
Phase resolution	100 %
Slice resolution	100 %
Phase partial Fourier	7/8
Slice partial Fourier	Off
Interpolation	Off

Resolution - iPAT

PAT mode	GRAPPA
Accel. factor PE	2
Ref. lines PE	24
Accel. factor 3D	1
Reference scan mode	Integrated

Resolution - Filter Image

Image Filter	Off
Distortion Corr.	Off
Prescan Normalize	On
Unfiltered images	Off
Normalize	Off
B1 filter	Off

Resolution - Filter Rawdata

Raw filter	Off
Elliptical filter	Off

Geometry - Common

Slab group	1
Slabs	1
Dist. factor	50 %
Position	R1.1 P38.9 F20.7 mm
Orientation	Sagittal
Phase enc. dir.	A >> P
Slice oversampling	0.0 %
Slices per slab	160
FoV read	160 mm
FoV phase	100.0 %
Slice thickness	1.00 mm
TR	1970.0 ms
Multi-slice mode	Single shot
Series	Interleaved
Concatenations	1

Geometry - AutoAlign

Slab group	1
Position	R1.1 P38.9 F20.7 mm
Orientation	Sagittal
Phase enc. dir.	A >> P
AutoAlign	---
Initial Position	R1.1 P38.9 F20.7
R	1.1 mm
P	38.9 mm
F	20.7 mm
Initial Rotation	0.00 deg
Initial Orientation	Sagittal

Geometry - Navigator

Geometry - Tim Planning Suite

Set-n-Go Protocol	Off
Table position	H
Table position	0 mm
Inline Composing	Off

System - Miscellaneous

Positioning mode	FIX
Table position	H
Table position	0 mm

Boardman JP, *et al. BMJ Open* 2020; 10:e035854. doi: 10.1136/bmjopen-2019-035854

SIEMENS MAGNETOM Prisma

System - Miscellaneous

MSMA	S - C - T
Sagittal	R >> L
Coronal	A >> P
Transversal	F >> H
Coil Combine Mode	Adaptive Combine
Save uncombined	Off
Matrix Optimization	Off
Coil Focus	Flat
AutoAlign	---
Coil Select Mode	On - AutoCoilSelect

System - Adjustments

B0 Shim mode	Standard
B1 Shim mode	TrueForm
Adjust with body coil	Off
Confirm freq. adjustment	Off
Assume Dominant Fat	Off
Assume Silicone	Off
Adjustment Tolerance	Auto

System - Adjust Volume

Position	R1.1 P38.9 F20.7 mm
Orientation	Sagittal
Rotation	0.00 deg
A >> P	160 mm
F >> H	160 mm
R >> L	160 mm
Reset	Off

System - pTx Volumes

B1 Shim mode	TrueForm
Excitation	Non-sel.

System - Tx/Rx

Frequency 1H	123.244318 MHz
Correction factor	1
Gain	Low
Img. Scale Cor.	4.000
Reset	Off
? Ref. amplitude 1H	0.000 V

Physio - Signal1

1st Signal/Mode	None
TR	1970.0 ms
Concatenations	1

Physio - Cardiac

Magn. preparation	Non-sel. IR
TI	1100 ms
Fat suppr.	None
Dark blood	Off
FoV read	160 mm
FoV phase	100.0 %
Phase resolution	100 %

Physio - PACE

Resp. control	Off
Concatenations	1

Inline - Common

Subtract	Off
Measurements	1
StdDev	Off

Inline - Common

Save original images	On

Inline - MIP

MIP-Sag	Off
MIP-Cor	Off
MIP-Tra	Off
MIP-Time	Off
Save original images	On

Inline - Composing

Inline Composing	Off
Distortion Corr.	Off

Inline - MapIt

Save original images	On
MapIt	None
Flip angle	9 deg
Measurements	1
TR	1970.0 ms
TE	4.69 ms

Sequence - Part 1

Introduction	On
Dimension	3D
Elliptical scanning	Off
Reordering	Linear
Asymmetric echo	Off
Flow comp.	No
Multi-slice mode	Single shot
Echo spacing	10.8 ms
Bandwidth	140 Hz/Px

Sequence - Part 2

RF pulse type	Normal
Gradient mode	Whisper
Excitation	Non-sel.
RF spoiling	On
Incr. Gradient spoiling	Off
Turbo factor	160

Sequence - Assistant

Mode	Off

Boardman JP, *et al. BMJ Open* 2020; 10:e035854. doi: 10.1136/bmjopen-2019-035854

SIEMENS MAGNETOM Prisma

\\Study Protocols\BRAIN\Neonates\Theirworld - E161723\SWI_v2
TA: 2:23 PM: FIX Voxel size: 0.8×0.8×3.0 mmPAT: 3 Rel. SNR: 1.00 : qswi_r

Properties

Prio recon	Off
Load images to viewer	On
Inline movie	Off
Auto store images	On
Load images to stamp segments	Off
Load images to graphic segments	Off
Auto open inline display	Off
Auto close inline display	Off
Start measurement without further preparation	Off
Wait for user to start	Off
Start measurements	Single measurement

Routine

Slab group	1
Slabs	1
Dist. factor	20 %
Position	L0.0 A2.3 H2.2 mm
Orientation	Transversal
Phase enc. dir.	R >> L
AutoAlign	---
Phase oversampling	0 %
Slice oversampling	20.0 %
Slices per slab	40
FoV read	240 mm
FoV phase	84.4 %
Slice thickness	3.00 mm
TR	28.0 ms
TE	20.00 ms
Averages	1
Concatenations	1
Filter	Prescan Normalize
Coil elements	HEA;HEP

Contrast - Common

TR	28.0 ms
TE	20.00 ms
MTC	Off
Magn. preparation	None
Flip angle	9 deg
Fat suppr.	None
Water suppr.	None
SWI	On

Contrast - Dynamic

Averages	1
Averaging mode	Short term
Reconstruction	Magn./Phase
Measurements	1
Multiple series	Each measurement

Resolution - Common

FoV read	240 mm
FoV phase	84.4 %
Slice thickness	3.00 mm
Base resolution	320
Phase resolution	100 %
Slice resolution	100 %
Phase partial Fourier	Off
Slice partial Fourier	Off

Resolution - Common

Interpolation	Off

Resolution - iPAT

PAT mode	GRAPPA
Accel. factor PE	3
Ref. lines PE	24
Accel. factor 3D	1
Reference scan mode	Integrated

Resolution - Filter Image

Image Filter	Off
Distortion Corr.	Off
Prescan Normalize	On
Unfiltered images	Off
Normalize	Off
B1 filter	Off

Resolution - Filter Rawdata

Raw filter	Off
Elliptical filter	Off

Geometry - Common

Slab group	1
Slabs	1
Dist. factor	20 %
Position	L0.0 A2.3 H2.2 mm
Orientation	Transversal
Phase enc. dir.	R >> L
Slice oversampling	20.0 %
Slices per slab	40
FoV read	240 mm
FoV phase	84.4 %
Slice thickness	3.00 mm
TR	28.0 ms
Multi-slice mode	Interleaved
Series	Interleaved
Concatenations	1

Geometry - AutoAlign

Slab group	1
Position	L0.0 A2.3 H2.2 mm
Orientation	Transversal
Phase enc. dir.	R >> L
AutoAlign	---
Initial Position	L0.0 A2.3 H2.2
L	0.0 mm
A	2.3 mm
H	2.2 mm
Initial Rotation	89.61 deg
Initial Orientation	Transversal

Geometry - Saturation

Saturation mode	Standard
Fat suppr.	None
Water suppr.	None
Special sat.	None

Geometry - Tim Planning Suite

Set-n-Go Protocol	Off
Table position	H

SIEMENS MAGNETOM Prisma

Geometry - Tim Planning Suite

Table position	0 mm
Inline Composing	Off

System - Miscellaneous

Positioning mode	FIX
Table position	H
Table position	0 mm
MSMA	S - C - T
Sagittal	R >> L
Coronal	A >> P
Transversal	F >> H
Coil Combine Mode	Adaptive Combine
Save uncombined	Off
Matrix Optimization	Off
AutoAlign	---
Coil Select Mode	On - AutoCoilSelect

System - Adjustments

B0 Shim mode	Standard
B1 Shim mode	TrueForm
Adjust with body coil	Off
Confirm freq. adjustment	Off
Assume Dominant Fat	Off
Assume Silicone	Off
Adjustment Tolerance	Auto

System - Adjust Volume

Position	L0.0 A2.3 H2.2 mm
Orientation	Transversal
Rotation	89.61 deg
R >> L	203 mm
A >> P	240 mm
F >> H	120 mm
Reset	Off

System - pTx Volumes

B1 Shim mode	TrueForm
Excitation	Slab-sel.

System - Tx/Rx

Frequency 1H	123.244318 MHz
Correction factor	1
Gain	Low
Img. Scale Cor.	1.000
Reset	Off
? Ref. amplitude 1H	0.000 V

Physio - Signal1

1st Signal/Mode	None
TR	28.0 ms
Concatenations	1
Segments	1

Physio - Cardiac

Tagging	None
Magn. preparation	None
Fat suppr.	None
Dark blood	Off
FoV read	240 mm
FoV phase	84.4 %
Phase resolution	100 %

Physio - PACE

Resp. control	Off
Concatenations	1

Inline - Common

Subtract	Off
Measurements	1
StdDev	Off
Liver registration	Off
Save original images	On

Inline - MIP

MIP-Sag	Off
MIP-Cor	Off
MIP-Tra	Off
MIP-Time	Off
Save original images	On

Inline - Soft Tissue

Wash - In	Off
Wash - Out	Off
TTP	Off
PEI	Off
MIP - time	Off
Measurements	1

Inline - Composing

Inline Composing	Off
Distortion Corr.	Off

Inline - MapIt

Save original images	On
MapIt	None
Flip angle	9 deg
Measurements	1
Contrasts	1
TR	28.0 ms
TE	20.00 ms

Sequence - Part 1

Introduction	On
Dimension	3D
Elliptical scanning	Off
Phase stabilisation	Off
Asymmetric echo	Off
Contrasts	1
Flow comp.	Yes
Multi-slice mode	Interleaved
Bandwidth	120 Hz/Px

Sequence - Part 2

Segments	1
Acoustic noise reduction	Active
RF pulse type	Fast
Gradient mode	Whisper
Excitation	Slab-sel.
RF spoiling	On

Sequence - Assistant

Mode	Off
Allowed delay	30 s

Boardman JP, *et al. BMJ Open* 2020; 10:e035854. doi: 10.1136/bmjopen-2019-035854

SIEMENS MAGNETOM Prisma

\\Study Protocols\BRAIN\Neonates\Theirworld - E161723\t2_blade_dark-fluid_tra_v3
TA: 3:22 PM: REF Voxel size: 0.9×0.9×3.0 mmPAT: 2 Rel. SNR: 1.00 : qtirB_rr

Properties

Prio recon	Off
Load images to viewer	On
Inline movie	Off
Auto store images	On
Load images to stamp segments	On
Load images to graphic segments	Off
Auto open inline display	Off
Auto close inline display	Off
Start measurement without further preparation	Off
Wait for user to start	Off
Start measurements	Single measurement

Routine

Slice group	1
Slices	40
Dist. factor	0 %
Position	Isocenter
Orientation	Transversal
Phase enc. dir.	R >> L
AutoAlign	---
Phase oversampling	0.0 %
FoV read	240 mm
FoV phase	100.0 %
Slice thickness	3.0 mm
TR	10000.0 ms
TE	130 ms
Averages	1
Concatenations	2
Filter	Prescan Normalize
Coil elements	HEA;HEP

Contrast - Common

TR	10000.0 ms
TE	130 ms
TD	0.0 ms
MTC	Off
Magn. preparation	Slice-sel. IR
TI	2606 ms
Flip angle	130 deg
Fat suppr.	Fat sat.
Fat sat. mode	Strong
Water suppr.	None
Restore magn.	Off
Freeze suppressed tissue	On

Contrast - Dynamic

Averages	1
Averaging mode	Short term
Reconstruction	Magnitude
Measurements	1
Multiple series	Each measurement

Resolution - Common

FoV read	240 mm
FoV phase	100.0 %
Slice thickness	3.0 mm
Base resolution	256
BLADE coverage	100.0 %
Trajectory	BLADE

Resolution - Common

Interpolation	Off

Resolution - iPAT

PAT mode	GRAPPA
Accel. factor PE	2
Ref. lines PE	8
Reference scan mode	Integrated

Resolution - Filter Image

Image Filter	Off
Distortion Corr.	Off
Prescan Normalize	On
Unfiltered images	Off
Normalize	Off
B1 filter	Off

Resolution - Filter Rawdata

Raw filter	Off
Elliptical filter	Off

Geometry - Common

Slice group	1
Slices	40
Dist. factor	0 %
Position	Isocenter
Orientation	Transversal
Phase enc. dir.	R >> L
FoV read	240 mm
FoV phase	100.0 %
Slice thickness	3.0 mm
TR	10000.0 ms
Multi-slice mode	Interleaved
Series	Interleaved
Concatenations	2

Geometry - AutoAlign

Slice group	1
Position	Isocenter
Orientation	Transversal
Phase enc. dir.	R >> L
AutoAlign	---
Initial Position	Isocenter
L	0.0 mm
P	0.0 mm
H	0.0 mm
Initial Rotation	90.00 deg
Initial Orientation	Transversal

Geometry - Saturation

Fat suppr.	Fat sat.
Fat sat. mode	Strong
Water suppr.	None
Restore magn.	Off
Special sat.	Parallel F
Gap	10 mm
Thickness	70 mm

Geometry - Navigator

SIEMENS MAGNETOM Prisma

Geometry - Tim Planning Suite

Set-n-Go Protocol	Off
Table position	H
Table position	0 mm
Inline Composing	Off

System - Miscellaneous

Positioning mode	REF
Table position	H
Table position	0 mm
MSMA	S - C - T
Sagittal	R >> L
Coronal	A >> P
Transversal	F >> H
Coil Combine Mode	Adaptive Combine
Save uncombined	Off
Matrix Optimization	Off
AutoAlign	---
Coil Select Mode	On - AutoCoilSelect

System - Adjustments

B0 Shim mode	Standard
B1 Shim mode	TrueForm
Adjust with body coil	Off
Confirm freq. adjustment	Off
Assume Dominant Fat	Off
Assume Silicone	Off
Adjustment Tolerance	Auto

System - Adjust Volume

Position	Isocenter
Orientation	Transversal
Rotation	90.00 deg
R >> L	240 mm
A >> P	240 mm
F >> H	120 mm
Reset	Off

System - pTx Volumes

B1 Shim mode	TrueForm

System - Tx/Rx

Frequency 1H	123.244318 MHz
Correction factor	1
Gain	High
Img. Scale Cor.	1.000
Reset	Off
? Ref. amplitude 1H	0.000 V

Physio - Signal1

1st Signal/Mode	None
TR	10000.0 ms
Concatenations	2

Physio - Cardiac

Magn. preparation	Slice-sel. IR
TI	2606 ms
Fat suppr.	Fat sat.
Dark blood	Off
FoV read	240 mm
FoV phase	100.0 %
BLADE coverage	100.0 %
Trajectory	BLADE

Physio - PACE

Resp. control	Off
Concatenations	2

Inline - Common

Subtract	Off
Measurements	1
StdDev	Off
Save original images	On

Inline - MIP

MIP-Sag	Off
MIP-Cor	Off
MIP-Tra	Off
MIP-Time	Off
Save original images	On

Inline - Composing

Inline Composing	Off
Distortion Corr.	Off

Sequence - Part 1

Introduction	On
Dimension	2D
Compensate T2 decay	Off
Contrasts	1
Flow comp.	Read
Multi-slice mode	Interleaved
Free echo spacing	Off
Echo spacing	8.64 ms
Bandwidth	362 Hz/Px

Sequence - Part 2

Define	Turbo factor
Echo trains per slice	9
Phase correction	Automatic
Acoustic noise reduction	Active
RF pulse type	Low SAR
Gradient mode	Normal
Hyperecho	Off
WARP	Off
Motion correction	On
Red. EC sensitivity	Off
Turbo factor	28

Sequence - Assistant

Mode	Min flip angle
Min flip angle	130 deg
Allowed delay	30 s

Boardman JP, *et al. BMJ Open* 2020; 10:e035854. doi: 10.1136/bmjopen-2019-035854
