# Supplementary material for: Impact of preterm birth on brain development and long-term outcome: protocol for a cohort study in Scotland
Source: BMJ Open. 2020 Mar 4;10(3):e035854. doi: 10.1136/bmjopen-2019-035854 (PMC7059503; doi:10.1136/bmjopen-2019-035854)
Supplement: Supplementary data [file bmjopen-2019-035854supp002.pdf]

```
# Author: qspace2siemens.m (Michael Thrippleton), manually edited
into 2 parts
# Source file: ./vector_tables/neonate/04-shells-3-6-64-64.txt
# b-value at UI: 750
# non-zero b-values: 750
# number of non-zero shells: 1
# number of directions per non-zero shell: 64
# number of b=0 volumes: 7
# total number of directions including b0: 71
[directions=71]
normalization = none
coordinatesystem = xyz
comment=bUI: 750, b: 750, Nb0: 7
vector[0] = ( 0.000000, 0.000000, 0.000000 )
vector[1] = ( -0.538981, 0.033731, -0.091439 )
vector[2] = ( -0.000440, 0.429608, 0.339760 )
vector[3] = ( -0.147395, -0.494556, -0.183546 )
vector[4] = ( 0.239035, -0.347062, 0.349872 )
vector[5] = ( -0.016278, -0.195328, 0.511451 )
vector[6] = ( -0.061295, -0.451376, 0.304143 )
vector[7] = ( 0.025626, -0.008709, -0.547053 )
vector[8] = ( -0.231133, -0.471788, 0.154896 )
vector[9] = ( -0.397538, -0.105537, -0.361699 )
vector[10] = ( 0.447399, -0.280126, -0.146162 )
vector[11] = ( 0.000000, 0.000000, 0.000000 )
vector[12] = ( -0.347344, -0.305418, 0.293379 )
vector[13] = ( 0.195148, -0.224679, 0.459823 )
vector[14] = ( 0.219722, 0.401006, -0.301523 )
vector[15] = ( 0.496386, 0.051099, 0.225809 )
vector[16] = ( -0.490022, 0.181524, -0.164098 )
vector[17] = ( 0.415886, 0.250359, 0.253691 )
vector[18] = ( 0.293795, 0.319409, 0.334159 )
vector[19] = ( 0.446457, -0.091032, 0.303955 )
vector[20] = ( 0.218923, -0.268898, -0.423989 )
vector[21] = ( -0.245685, -0.236576, 0.428568 )
vector[22] = ( 0.000000, 0.000000, 0.000000 )
vector[23] = ( 0.023434, -0.514342, -0.186823 )
vector[24] = ( 0.210090, -0.495890, -0.099773 )
vector[25] = ( 0.127918, 0.282591, 0.451419 )
vector[26] = ( -0.497742, -0.190842, -0.125826 )
vector[27] = ( -0.352216, -0.116300, 0.403012 )
vector[28] = ( -0.439047, 0.004691, 0.327438 )
vector[29] = ( 0.143700, -0.138995, -0.509932 )
vector[30] = ( -0.483604, 0.256940, -0.010438 )
vector[31] = ( 0.536886, 0.108072, -0.008594 )
vector[32] = ( -0.113008, -0.337640, 0.416207 )
vector[33] = ( 0.000000, 0.000000, 0.000000 )
vector[34] = ( 0.346021, -0.402459, -0.135263 )
vector[35] = ( -0.172278, 0.446108, 0.267035 )
vector[36] = ( -0.309270, 0.076830, -0.445476 )
vector[37] = ( 0.274066, -0.423055, 0.214272 )
vector[38] = ( 0.052227, -0.321802, 0.440132 )
vector[39] = ( 0.075465, 0.519169, -0.157382 )
vector[40] = ( 0.152874, 0.405328, 0.335170 )
```

```
vector[41] = ( 0.109576, 0.536320, 0.018825 )
vector[42] = ( -0.045652, 0.300780, 0.455464 )
vector[43] = ( 0.000000, 0.000000, 0.000000 )
vector[44] = ( -0.533887, 0.114345, 0.043471 )
vector[45] = ( -0.097529, 0.434255, -0.319235 )
vector[46] = ( 0.391774, -0.236122, -0.301263 )
vector[47] = ( 0.399513, -0.317429, 0.199068 )
vector[48] = ( 0.200167, 0.067226, 0.505385 )
vector[49] = ( 0.385668, -0.387145, 0.037137 )
vector[50] = ( 0.059543, 0.145424, 0.524697 )
vector[51] = ( -0.445546, -0.189946, 0.255752 )
vector[52] = ( 0.263180, -0.007998, -0.480284 )
vector[53] = ( -0.375132, -0.375662, 0.134735 )
vector[54] = ( 0.000000, 0.000000, 0.000000 )
vector[55] = ( -0.100958, 0.513042, -0.163080 )
vector[56] = ( 0.266095, 0.478340, 0.019604 )
vector[57] = ( 0.480516, -0.133538, -0.226434 )
vector[58] = ( 0.253431, -0.482875, 0.051025 )
vector[59] = ( 0.361384, -0.227994, 0.342667 )
vector[60] = ( -0.479164, -0.248769, 0.092279 )
vector[61] = ( -0.422438, -0.343026, -0.062282 )
vector[62] = ( 0.525823, 0.037772, -0.148605 )
vector[63] = ( 0.112166, -0.092301, 0.528109 )
vector[64] = ( 0.050487, -0.545354, 0.006363 )
vector[65] = ( 0.000000, 0.000000, 0.000000 )
vector[66] = ( -0.290577, 0.355116, 0.299095 )
vector[67] = ( -0.303506, -0.415037, -0.188755 )
vector[68] = ( -0.340501, 0.129187, 0.409109 )
vector[69] = ( -0.275521, -0.188617, -0.434179 )
vector[70] = ( 0.148849, 0.097956, -0.517928 )
```
