# Supplementary material for: Impact of preterm birth on brain development and long-term outcome: protocol for a cohort study in Scotland
Source: BMJ Open. 2020 Mar 4;10(3):e035854. doi: 10.1136/bmjopen-2019-035854 (PMC7059503; doi:10.1136/bmjopen-2019-035854)
Supplement: Supplementary data [file bmjopen-2019-035854supp003.pdf]

```
# Author: qspace2siemens.m (Michael Thrippleton), manually edited
into 2 parts
# Source file: ./vector_tables/neonate/04-shells-3-6-64-64.txt
# b-value at UI: 2500
# non-zero b-values: 200   500  2500
# number of non-zero shells: 2
# number of directions per non-zero shell: 3   6  64
# number of b=0 volumes: 7
# total number of directions including b0: 151
[directions=80]
normalization = none
coordinatesystem = xyz
comment=bUI: 2500, b: 200   500   2500, Nb0: 7
vector[0] = ( 0.000000, 0.000000, 0.000000 )
vector[1] = ( 0.252007, 0.053675, -0.116668 )
vector[2] = ( 0.118341, -0.013011, 0.256566 )
vector[3] = ( 0.047528, -0.276133, -0.038625 )
vector[4] = ( -0.303298, -0.002700, -0.328638 )
vector[5] = ( -0.128927, -0.159163, 0.397549 )
vector[6] = ( 0.288240, 0.341931, 0.000938 )
vector[7] = ( -0.166829, 0.397185, -0.120052 )
vector[8] = ( -0.069301, 0.303423, 0.321142 )
vector[9] = ( 0.425645, -0.074339, -0.115324 )
vector[10] = ( 0.391424, -0.221918, 0.893051 )
vector[11] = ( 0.458593, -0.241695, -0.855147 )
vector[12] = ( 0.354539, 0.919288, 0.170913 )
vector[13] = ( 0.495263, -0.780339, -0.381819 )
vector[14] = ( -0.574230, 0.458191, 0.678470 )
vector[15] = ( 0.000000, 0.000000, 0.000000 )
vector[16] = ( -0.188453, -0.033220, -0.981520 )
vector[17] = ( 0.594951, -0.772279, 0.222754 )
vector[18] = ( 0.076963, -0.202692, -0.976213 )
vector[19] = ( -0.354234, 0.663631, 0.658872 )
vector[20] = ( -0.245839, 0.923577, 0.294225 )
vector[21] = ( -0.646526, -0.378550, -0.662347 )
vector[22] = ( 0.782685, 0.616196, -0.087788 )
vector[23] = ( -0.102171, -0.675368, -0.730369 )
vector[24] = ( -0.593833, 0.627627, -0.503435 )
vector[25] = ( -0.289839, 0.954652, -0.068065 )
vector[26] = ( 0.000000, 0.000000, 0.000000 )
vector[27] = ( 0.932852, 0.268018, -0.240735 )
vector[28] = ( -0.292661, 0.011816, 0.956143 )
vector[29] = ( -0.125932, -0.877649, -0.462465 )
vector[30] = ( 0.287138, 0.947828, -0.138468 )
vector[31] = ( -0.400507, -0.785392, -0.471967 )
vector[32] = ( 0.046561, 0.178494, -0.982839 )
vector[33] = ( 0.774106, -0.243372, -0.584405 )
vector[34] = ( -0.709331, 0.570685, 0.413724 )
vector[35] = ( 0.258673, -0.649858, 0.714684 )
vector[36] = ( 0.000000, 0.000000, 0.000000 )
vector[37] = ( 0.812504, 0.520520, 0.262482 )
vector[38] = ( -0.551995, -0.116325, -0.825694 )
vector[39] = ( -0.680119, 0.223136, -0.698319 )
vector[40] = ( -0.848362, -0.280672, -0.448893 )
```

```
vector[41] = ( -0.460227, -0.230447, 0.857371 )
vector[42] = ( 0.639224, 0.615748, 0.460703 )
vector[43] = ( 0.953358, -0.285443, 0.098132 )
vector[44] = ( -0.501430, 0.459528, -0.733077 )
vector[45] = ( 0.922461, 0.385130, 0.027209 )
vector[46] = ( -0.815410, 0.546002, -0.192323 )
vector[47] = ( 0.000000, 0.000000, 0.000000 )
vector[48] = ( -0.924442, 0.129694, -0.358591 )
vector[49] = ( 0.549990, 0.820347, -0.156657 )
vector[50] = ( 0.774802, 0.509647, -0.374089 )
vector[51] = ( 0.907672, -0.355700, -0.222731 )
vector[52] = ( 0.051712, 0.985317, 0.162714 )
vector[53] = ( -0.970546, -0.135098, -0.199471 )
vector[54] = ( -0.621107, -0.417526, 0.663249 )
vector[55] = ( -0.776136, 0.621968, 0.103774 )
vector[56] = ( 0.551897, -0.830144, -0.079188 )
vector[57] = ( 0.555009, 0.711394, -0.431142 )
vector[58] = ( 0.000000, 0.000000, 0.000000 )
vector[59] = ( -0.239295, 0.451777, 0.859439 )
vector[60] = ( -0.325801, -0.314211, -0.891698 )
vector[61] = ( 0.649939, -0.012663, -0.759881 )
vector[62] = ( -0.042327, 0.894181, -0.445699 )
vector[63] = ( -0.159022, 0.408833, -0.898648 )
vector[64] = ( 0.388219, 0.606776, -0.693620 )
vector[65] = ( -0.329997, 0.825600, -0.457697 )
vector[66] = ( 0.060764, 0.443276, 0.894323 )
vector[67] = ( -0.794452, 0.390958, -0.464756 )
vector[68] = ( -0.392295, -0.567128, -0.724204 )
vector[69] = ( 0.000000, 0.000000, 0.000000 )
vector[70] = ( 0.272234, 0.851327, -0.448477 )
vector[71] = ( 0.785891, 0.193927, -0.587169 )
vector[72] = ( -0.145787, 0.828569, 0.540573 )
vector[73] = ( 0.616784, 0.765973, 0.181281 )
vector[74] = ( -0.808755, -0.029868, -0.587387 )
vector[75] = ( 0.997247, -0.010658, -0.073384 )
vector[76] = ( -0.152743, -0.477444, 0.865284 )
vector[77] = ( -0.040188, -0.715882, 0.697064 )
vector[78] = ( -0.907740, 0.040990, 0.417525 )
vector[79] = ( 0.008357, -0.985450, 0.169758 )
```
