# Supplementary material for: Impact of preterm birth on brain development and long-term outcome: protocol for a cohort study in Scotland
Source: BMJ Open. 2020 Mar 4;10(3):e035854. doi: 10.1136/bmjopen-2019-035854 (PMC7059503; doi:10.1136/bmjopen-2019-035854)
Supplement: Supplementary data [file bmjopen-2019-035854supp004.pdf]

SIEMENS MAGNETOM Prisma

\\Study Protocols\BRAIN\Neonates\Theirworld - E161723 - MT_test\MTSatOn_neonate_v2
TA: 2:58 PM: REF Voxel size: 2.0×2.0×2.0 mmPAT: 3 Rel. SNR: 1.00 : qfl

Properties

Prio recon	Off
Load images to viewer	On
Inline movie	Off
Auto store images	On
Load images to stamp segments	Off
Load images to graphic segments	Off
Auto open inline display	Off
Auto close inline display	Off
Start measurement without further preparation	Off
Wait for user to start	Off
Start measurements	Single measurement

Routine

Slab group	1
Slabs	1
Dist. factor	20 %
Position	R6.7 P19.4 H34.5 mm
Orientation	Sagittal
Phase enc. dir.	A >> P
AutoAlign	---
Phase oversampling	0 %
Slice oversampling	0.0 %
Slices per slab	72
FoV read	128 mm
FoV phase	121.9 %
Slice thickness	2.00 mm
TR	75.0 ms
TE 1	1.54 ms
TE 2	4.55 ms
TE 3	8.56 ms
Averages	1
Concatenations	1
Filter	Prescan Normalize
Coil elements	PeH;PeN

Contrast - Common

TR	75.0 ms
TE 1	1.54 ms
TE 2	4.55 ms
TE 3	8.56 ms
MTC	On
Magn. preparation	None
Flip angle	5 deg
Fat suppr.	None
Water suppr.	None
SWI	Off

Contrast - Dynamic

Averages	1
Averaging mode	Short term
Reconstruction	Magnitude
Measurements	1
Multiple series	Each measurement

Resolution - Common

FoV read	128 mm
FoV phase	121.9 %
Slice thickness	2.00 mm
Base resolution	64

Resolution - Common

Phase resolution	100 %
Slice resolution	100 %
Phase partial Fourier	6/8
Slice partial Fourier	Off
Interpolation	Off

Resolution - iPAT

PAT mode	GRAPPA
Accel. factor PE	3
Ref. lines PE	24
Accel. factor 3D	1
Reference scan mode	Integrated

Resolution - Filter Image

Image Filter	Off
Distortion Corr.	Off
Prescan Normalize	On
Unfiltered images	Off
Normalize	Off
B1 filter	Off

Resolution - Filter Rawdata

Raw filter	Off
Elliptical filter	Off

Geometry - Common

Slab group	1
Slabs	1
Dist. factor	20 %
Position	R6.7 P19.4 H34.5 mm
Orientation	Sagittal
Phase enc. dir.	A >> P
Slice oversampling	0.0 %
Slices per slab	72
FoV read	128 mm
FoV phase	121.9 %
Slice thickness	2.00 mm
TR	75.0 ms
Multi-slice mode	Interleaved
Series	Interleaved
Concatenations	1

Geometry - AutoAlign

Slab group	1
Position	R6.7 P19.4 H34.5 mm
Orientation	Sagittal
Phase enc. dir.	A >> P
AutoAlign	---
Initial Position	R6.7 P19.4 H34.5
R	6.7 mm
P	19.4 mm
H	34.5 mm
Initial Rotation	0.00 deg
Initial Orientation	Sagittal

Geometry - Saturation

Saturation mode	Standard
Fat suppr.	None
Water suppr.	None
Special sat.	None

SIEMENS MAGNETOM Prisma

Geometry - Tim Planning Suite

Set-n-Go Protocol	Off
Table position	H
Table position	0 mm
Inline Composing	Off

System - Miscellaneous

Positioning mode	REF
Table position	H
Table position	0 mm
MSMA	S - C - T
Sagittal	R >> L
Coronal	A >> P
Transversal	F >> H
Coil Combine Mode	Sum of Squares
Save uncombined	Off
Matrix Optimization	Off
AutoAlign	---
Coil Select Mode	Off - AutoCoilSelect

System - Adjustments

B0 Shim mode	Tune up
B1 Shim mode	TrueForm
Adjust with body coil	Off
Confirm freq. adjustment	Off
Assume Dominant Fat	Off
Assume Silicone	Off
Adjustment Tolerance	Auto

System - Adjust Volume

Position	Isocenter
Orientation	Transversal
Rotation	0.00 deg
A >> P	263 mm
R >> L	350 mm
F >> H	350 mm
Reset	Off

System - pTx Volumes

B1 Shim mode	TrueForm
Excitation	Non-sel.

System - Tx/Rx

Frequency 1H	123.244480 MHz
Correction factor	1
Gain	Low
Img. Scale Cor.	3.000
Reset	Off
? Ref. amplitude 1H	0.000 V

Physio - Signal1

1st Signal/Mode	None
TR	75.0 ms
Concatenations	1
Segments	1

Physio - Cardiac

Tagging	None
Magn. preparation	None
Fat suppr.	None
Dark blood	Off
FoV read	128 mm
FoV phase	121.9 %
Phase resolution	100 %

Physio - PACE

Resp. control	Off
Concatenations	1

Inline - Common

Subtract	Off
Measurements	1
StdDev	Off
Liver registration	Off
Save original images	On

Inline - MIP

MIP-Sag	Off
MIP-Cor	Off
MIP-Tra	Off
MIP-Time	Off
Save original images	On

Inline - Soft Tissue

Wash - In	Off
Wash - Out	Off
TTP	Off
PEI	Off
MIP - time	Off
Measurements	1

Inline - Composing

Inline Composing	Off
Distortion Corr.	Off

Inline - MapIt

Save original images	On
MapIt	None
Flip angle	5 deg
Measurements	1
Contrasts	3
TR	75.0 ms
TE 1	1.54 ms
TE 2	4.55 ms
TE 3	8.56 ms

Sequence - Part 1

Introduction	Off
Dimension	3D
Elliptical scanning	On
Phase stabilisation	Off
Asymmetric echo	Off
Contrasts	3
Flow comp. 1	No
Readout mode	Bipolar
Multi-slice mode	Interleaved
Bandwidth 1	580 Hz/Px
Bandwidth 2	580 Hz/Px
Bandwidth 3	580 Hz/Px

Sequence - Part 2

Segments	1
Acoustic noise reduction	Active
RF pulse type	Low SAR
Gradient mode	Normal
Excitation	Non-sel.
RF spoiling	On

Boardman JP, *et al. BMJ Open* 2020; 10:e035854. doi: 10.1136/bmjopen-2019-035854

SIEMENS MAGNETOM Prisma

Sequence - Assistant

Mode	Off
Allowed delay	30 s

Boardman JP, *et al. BMJ Open* 2020; 10:e035854. doi: 10.1136/bmjopen-2019-035854

SIEMENS MAGNETOM Prisma

\\Study Protocols\BRAIN\Neonates\Theirworld - E161723 - MT_test\MTSatOff_neonate_v2

TA: 2:58 PM: FIX Voxel size: 2.0×2.0×2.0 mmPAT: 3 Rel. SNR: 1.00 : qfl

Properties

Prio recon	Off
Load images to viewer	On
Inline movie	Off
Auto store images	On
Load images to stamp segments	Off
Load images to graphic segments	Off
Auto open inline display	Off
Auto close inline display	Off
Start measurement without further preparation	On
Wait for user to start	Off
Start measurements	Single measurement

Routine

Slab group	1
Slabs	1
Dist. factor	20 %
Position	R6.7 P19.4 H34.5 mm
Orientation	Sagittal
Phase enc. dir.	A >> P
AutoAlign	---
Phase oversampling	0 %
Slice oversampling	0.0 %
Slices per slab	72
FoV read	128 mm
FoV phase	121.9 %
Slice thickness	2.00 mm
TR	75.0 ms
TE 1	1.54 ms
TE 2	4.55 ms
TE 3	8.56 ms
Averages	1
Concatenations	1
Filter	Prescan Normalize
Coil elements	PeH;PeN

Contrast - Common

TR	75.0 ms
TE 1	1.54 ms
TE 2	4.55 ms
TE 3	8.56 ms
MTC	Off
Magn. preparation	None
Flip angle	5 deg
Fat suppr.	None
Water suppr.	None
SWI	Off

Contrast - Dynamic

Averages	1
Averaging mode	Short term
Reconstruction	Magnitude
Measurements	1
Multiple series	Each measurement

Resolution - Common

FoV read	128 mm
FoV phase	121.9 %
Slice thickness	2.00 mm
Base resolution	64

Resolution - Common

Phase resolution	100 %
Slice resolution	100 %
Phase partial Fourier	6/8
Slice partial Fourier	Off
Interpolation	Off

Resolution - iPAT

PAT mode	GRAPPA
Accel. factor PE	3
Ref. lines PE	24
Accel. factor 3D	1
Reference scan mode	Integrated

Resolution - Filter Image

Image Filter	Off
Distortion Corr.	Off
Prescan Normalize	On
Unfiltered images	Off
Normalize	Off
B1 filter	Off

Resolution - Filter Rawdata

Raw filter	Off
Elliptical filter	Off

Geometry - Common

Slab group	1
Slabs	1
Dist. factor	20 %
Position	R6.7 P19.4 H34.5 mm
Orientation	Sagittal
Phase enc. dir.	A >> P
Slice oversampling	0.0 %
Slices per slab	72
FoV read	128 mm
FoV phase	121.9 %
Slice thickness	2.00 mm
TR	75.0 ms
Multi-slice mode	Interleaved
Series	Interleaved
Concatenations	1

Geometry - AutoAlign

Slab group	1
Position	R6.7 P19.4 H34.5 mm
Orientation	Sagittal
Phase enc. dir.	A >> P
AutoAlign	---
Initial Position	R6.7 P19.4 H34.5
R	6.7 mm
P	19.4 mm
H	34.5 mm
Initial Rotation	0.00 deg
Initial Orientation	Sagittal

Geometry - Saturation

Saturation mode	Standard
Fat suppr.	None
Water suppr.	None
Special sat.	None

Boardman JP, *et al. BMJ Open* 2020; 10:e035854. doi: 10.1136/bmjopen-2019-035854

SIEMENS MAGNETOM Prisma

Geometry - Tim Planning Suite

Set-n-Go Protocol	Off
Table position	H
Table position	0 mm
Inline Composing	Off

System - Miscellaneous

Positioning mode	FIX
Table position	H
Table position	0 mm
MSMA	S - C - T
Sagittal	R >> L
Coronal	A >> P
Transversal	F >> H
Coil Combine Mode	Sum of Squares
Save uncombined	Off
Matrix Optimization	Off
AutoAlign	---
Coil Select Mode	Off - AutoCoilSelect

System - Adjustments

B0 Shim mode	Tune up
B1 Shim mode	TrueForm
Adjust with body coil	Off
Confirm freq. adjustment	Off
Assume Dominant Fat	Off
Assume Silicone	Off
Adjustment Tolerance	Auto

System - Adjust Volume

Position	Isocenter
Orientation	Transversal
Rotation	0.00 deg
A >> P	263 mm
R >> L	350 mm
F >> H	350 mm
Reset	Off

System - pTx Volumes

B1 Shim mode	TrueForm
Excitation	Non-sel.

System - Tx/Rx

Frequency 1H	123.244480 MHz
Correction factor	1
Gain	Low
Img. Scale Cor.	3.000
Reset	Off
? Ref. amplitude 1H	0.000 V

Physio - Signal1

1st Signal/Mode	None
TR	75.0 ms
Concatenations	1
Segments	1

Physio - Cardiac

Tagging	None
Magn. preparation	None
Fat suppr.	None
Dark blood	Off
FoV read	128 mm
FoV phase	121.9 %
Phase resolution	100 %

Physio - PACE

Resp. control	Off
Concatenations	1

Inline - Common

Subtract	Off
Measurements	1
StdDev	Off
Liver registration	Off
Save original images	On

Inline - MIP

MIP-Sag	Off
MIP-Cor	Off
MIP-Tra	Off
MIP-Time	Off
Save original images	On

Inline - Soft Tissue

Wash - In	Off
Wash - Out	Off
TTP	Off
PEI	Off
MIP - time	Off
Measurements	1

Inline - Composing

Inline Composing	Off
Distortion Corr.	Off

Inline - MapIt

Save original images	On
MapIt	None
Flip angle	5 deg
Measurements	1
Contrasts	3
TR	75.0 ms
TE 1	1.54 ms
TE 2	4.55 ms
TE 3	8.56 ms

Sequence - Part 1

Introduction	Off
Dimension	3D
Elliptical scanning	On
Phase stabilisation	Off
Asymmetric echo	Off
Contrasts	3
Flow comp. 1	No
Readout mode	Bipolar
Multi-slice mode	Interleaved
Bandwidth 1	580 Hz/Px
Bandwidth 2	580 Hz/Px
Bandwidth 3	580 Hz/Px

Sequence - Part 2

Segments	1
Acoustic noise reduction	Active
RF pulse type	Low SAR
Gradient mode	Normal
Excitation	Non-sel.
RF spoiling	On

Boardman JP, *et al. BMJ Open* 2020; 10:e035854. doi: 10.1136/bmjopen-2019-035854

SIEMENS MAGNETOM Prisma

Sequence - Assistant

Mode	Off
Allowed delay	30 s

Boardman JP, *et al. BMJ Open* 2020; 10:e035854. doi: 10.1136/bmjopen-2019-035854

SIEMENS MAGNETOM Prisma

\\Study Protocols\BRAIN\Neonates\Theirworld - E161723 - MT_test\MTSatT1_neonate_v2

TA: 0:36 PM: FIX Voxel size: 2.0×2.0×2.0 mmPAT: 3 Rel. SNR: 1.00 : qfl

Properties

Prio recon	Off
Load images to viewer	On
Inline movie	Off
Auto store images	On
Load images to stamp segments	Off
Load images to graphic segments	Off
Auto open inline display	Off
Auto close inline display	Off
Start measurement without further preparation	On
Wait for user to start	Off
Start measurements	Single measurement

Routine

Slab group	1
Slabs	1
Dist. factor	20 %
Position	R6.7 P19.4 H34.5 mm
Orientation	Sagittal
Phase enc. dir.	A >> P
AutoAlign	---
Phase oversampling	0 %
Slice oversampling	0.0 %
Slices per slab	72
FoV read	128 mm
FoV phase	121.9 %
Slice thickness	2.00 mm
TR	15.0 ms
TE 1	1.54 ms
TE 2	4.55 ms
TE 3	8.56 ms
Averages	1
Concatenations	1
Filter	Prescan Normalize
Coil elements	PeH;PeN

Contrast - Common

TR	15.0 ms
TE 1	1.54 ms
TE 2	4.55 ms
TE 3	8.56 ms
MTC	Off
Magn. preparation	None
Flip angle	14 deg
Fat suppr.	None
Water suppr.	None
SWI	Off

Contrast - Dynamic

Averages	1
Averaging mode	Short term
Reconstruction	Magnitude
Measurements	1
Multiple series	Each measurement

Resolution - Common

FoV read	128 mm
FoV phase	121.9 %
Slice thickness	2.00 mm
Base resolution	64

Resolution - Common

Phase resolution	100 %
Slice resolution	100 %
Phase partial Fourier	6/8
Slice partial Fourier	Off
Interpolation	Off

Resolution - iPAT

PAT mode	GRAPPA
Accel. factor PE	3
Ref. lines PE	24
Accel. factor 3D	1
Reference scan mode	Integrated

Resolution - Filter Image

Image Filter	Off
Distortion Corr.	Off
Prescan Normalize	On
Unfiltered images	Off
Normalize	Off
B1 filter	Off

Resolution - Filter Rawdata

Raw filter	Off
Elliptical filter	Off

Geometry - Common

Slab group	1
Slabs	1
Dist. factor	20 %
Position	R6.7 P19.4 H34.5 mm
Orientation	Sagittal
Phase enc. dir.	A >> P
Slice oversampling	0.0 %
Slices per slab	72
FoV read	128 mm
FoV phase	121.9 %
Slice thickness	2.00 mm
TR	15.0 ms
Multi-slice mode	Interleaved
Series	Interleaved
Concatenations	1

Geometry - AutoAlign

Slab group	1
Position	R6.7 P19.4 H34.5 mm
Orientation	Sagittal
Phase enc. dir.	A >> P
AutoAlign	---
Initial Position	R6.7 P19.4 H34.5
R	6.7 mm
P	19.4 mm
H	34.5 mm
Initial Rotation	0.00 deg
Initial Orientation	Sagittal

Geometry - Saturation

Saturation mode	Standard
Fat suppr.	None
Water suppr.	None
Special sat.	None

Boardman JP, *et al. BMJ Open* 2020; 10:e035854. doi: 10.1136/bmjopen-2019-035854

SIEMENS MAGNETOM Prisma

Geometry - Tim Planning Suite

Set-n-Go Protocol	Off
Table position	H
Table position	0 mm
Inline Composing	Off

System - Miscellaneous

Positioning mode	FIX
Table position	H
Table position	0 mm
MSMA	S - C - T
Sagittal	R >> L
Coronal	A >> P
Transversal	F >> H
Coil Combine Mode	Sum of Squares
Save uncombined	Off
Matrix Optimization	Off
AutoAlign	---
Coil Select Mode	Off - AutoCoilSelect

System - Adjustments

B0 Shim mode	Tune up
B1 Shim mode	TrueForm
Adjust with body coil	Off
Confirm freq. adjustment	Off
Assume Dominant Fat	Off
Assume Silicone	Off
Adjustment Tolerance	Auto

System - Adjust Volume

Position	Isocenter
Orientation	Transversal
Rotation	0.00 deg
A >> P	263 mm
R >> L	350 mm
F >> H	350 mm
Reset	Off

System - pTx Volumes

B1 Shim mode	TrueForm
Excitation	Non-sel.

System - Tx/Rx

Frequency 1H	123.244480 MHz
Correction factor	1
Gain	Low
Img. Scale Cor.	3.000
Reset	Off
? Ref. amplitude 1H	0.000 V

Physio - Signal1

1st Signal/Mode	None
TR	15.0 ms
Concatenations	1
Segments	1

Physio - Cardiac

Tagging	None
Magn. preparation	None
Fat suppr.	None
Dark blood	Off
FoV read	128 mm
FoV phase	121.9 %
Phase resolution	100 %

Physio - PACE

Resp. control	Off
Concatenations	1

Inline - Common

Subtract	Off
Measurements	1
StdDev	Off
Liver registration	Off
Save original images	On

Inline - MIP

MIP-Sag	Off
MIP-Cor	Off
MIP-Tra	Off
MIP-Time	Off
Save original images	On

Inline - Soft Tissue

Wash - In	Off
Wash - Out	Off
TTP	Off
PEI	Off
MIP - time	Off
Measurements	1

Inline - Composing

Inline Composing	Off
Distortion Corr.	Off

Inline - MapIt

Save original images	On
MapIt	None
Flip angle	14 deg
Measurements	1
Contrasts	3
TR	15.0 ms
TE 1	1.54 ms
TE 2	4.55 ms
TE 3	8.56 ms

Sequence - Part 1

Introduction	Off
Dimension	3D
Elliptical scanning	On
Phase stabilisation	Off
Asymmetric echo	Off
Contrasts	3
Flow comp. 1	No
Readout mode	Bipolar
Multi-slice mode	Interleaved
Bandwidth 1	580 Hz/Px
Bandwidth 2	580 Hz/Px
Bandwidth 3	580 Hz/Px

Sequence - Part 2

Segments	1
Acoustic noise reduction	Active
RF pulse type	Low SAR
Gradient mode	Normal
Excitation	Non-sel.
RF spoiling	On

Boardman JP, *et al. BMJ Open* 2020; 10:e035854. doi: 10.1136/bmjopen-2019-035854

SIEMENS MAGNETOM Prisma

Sequence - Assistant

Mode	Off
Allowed delay	30 s

Boardman JP, *et al. BMJ Open* 2020; 10:e035854. doi: 10.1136/bmjopen-2019-035854
