# Supplementary material for: Impact of preterm birth on brain development and long-term outcome: protocol for a cohort study in Scotland
Source: BMJ Open. 2020 Mar 4;10(3):e035854. doi: 10.1136/bmjopen-2019-035854 (PMC7059503; doi:10.1136/bmjopen-2019-035854)
Supplement: Author's manuscript [file bmjopen-2019-035854.draft_revisions.pdf]

BMJ Open is committed to open peer review. As part of this commitment we make the peer review history of every article we publish publicly available.

When an article is published we post the peer reviewers' comments and the authors' responses online. We also post the versions of the paper that were used during peer review. These are the versions that the peer review comments apply to.

The versions of the paper that follow are the versions that were submitted during the peer review process. They are not the versions of record or the final published versions. They should not be cited or distributed as the published version of this manuscript.

BMJ Open is an open access journal and the full, final, typeset and author-corrected version of record of the manuscript is available on our site with no access controls, subscription charges or pay-per-view fees (http://bmjopen.bmj.com).

If you have any questions on BMJ Open's open peer review process please email info.bmjopen@bmj.com

BMJ Open

Protocol: Theirworld Edinburgh Birth Cohort

Journal:	*BMJ Open*
Manuscript ID	bmjopen-2019-035854
Article Type:	Protocol
Date Submitted by the Author:	19-Nov-2019

Complete List of Authors:	Boardman, James; The University of Edinburgh, MRC Centre for Reproductive Health; The University of Edinburgh, Centre for Clinical Brain Sciences<br>Hall, Jill; The University of Edinburgh, MRC Centre for Reproductive Health<br>Thrippleton, Michael; The University of Edinburgh, Edinburgh Imaging<br>Reynolds, Rebecca; The University of Edinburgh, Centre for Cardiovascular Science<br>Bogaert, Debby; The University of Edinburgh, Centre for Inflammation Research<br>Davidson, Donald ; The University of Edinburgh, Centre for Inflammation Research<br>Schwarze, Jurgen; The University of Edinburgh, Centre for Inflammation Research<br>Drake, Amanda; The University of Edinburgh, Centre for Cardiovascular Sciences<br>Chandran, Siddharthan ; The University of Edinburgh, Centre for Clinical Brain Sciences<br>Bastin, Mark; The University of Edinburgh, Centre for Clinical Brain Sciences<br>Fletcher-Watson, Sue; The University of Edinburgh, Centre for Clinical Brain Sciences

Keywords:	Neonatal intensive & critical care < INTENSIVE & CRITICAL CARE, Paediatric neurology < NEUROLOGY, Maternal medicine < OBSTETRICS, Developmental neurology & neurodisability < PAEDIATRICS, Child & adolescent psychiatry < PSYCHIATRY, Magnetic resonance imaging < RADIOLOGY & IMAGING

SCHOLARONE™
Manuscripts

I, the Submitting Author has the right to grant and does grant on behalf of all authors of the Work (as defined in the below author licence), an exclusive licence and/or a non-exclusive licence for contributions from authors who are: i) UK Crown employees; ii) where BMJ has agreed a CC-BY licence shall apply, and/or iii) in accordance with the terms applicable for US Federal Government officers or employees acting as part of their official duties; on a worldwide, perpetual, irrevocable, royalty-free basis to BMJ Publishing Group Ltd ("BMJ") its licensees and where the relevant Journal is co-owned by BMJ to the co-owners of the Journal, to publish the Work in this journal and any other BMJ products and to exploit all rights, as set out in our licence.

The Submitting Author accepts and understands that any supply made under these terms is made by BMJ to the Submitting Author unless you are acting as an employee on behalf of your employer or a postgraduate student of an affiliated institution which is paying any applicable article publishing charge ("APC") for Open Access articles. Where the Submitting Author wishes to make the Work available on an Open Access basis (and intends to pay the relevant APC), the terms of reuse of such Open Access shall be governed by a Creative Commons licence – details of these licences and which Creative Commons licence will apply to this Work are set out in our licence referred to above.

Other than as permitted in any relevant BMJ Author's Self Archiving Policies, I confirm this Work has not been accepted for publication elsewhere, is not being considered for publication elsewhere and does not duplicate material already published. I confirm all authors consent to publication of this Work and authorise the granting of this licence.

For peer review only

Protocol: Theirworld Edinburgh Birth Cohort

James P Boardman[1,2], Jill Hall[1], Michael J Thrippleton[3], Rebecca M Reynolds[4], Debby Bogaert[5], Donald J. Davidson[5], Jürgen Schwarze[5], Amanda J Drake[4], Siddharthan Chandran[2], Mark E Bastin[2], Sue Fletcher-Watson[2]

[1] Medical Research Centre for Reproductive Health, University of Edinburgh, Edinburgh, UK

[2] Centre for Clinical Brain Sciences, University of Edinburgh, Edinburgh, UK

[3] Edinburgh Imaging, University of Edinburgh, UK

[4]Centre for Cardiovascular Science, University of Edinburgh, Edinburgh, UK

[5] University of Edinburgh Centre for Inflammation Research, Edinburgh, UK

Corresponding author:

Professor James Peter Boardman

W1.26 Queen's Medical Research Institute,

47 Little France Crescent,

Edinburgh EH16 4TJ,

United Kingdom.

T: +44 131 242 2567

E: james.boardman@ed.ac.uk

Word count (excl title page, abstract, references, figures and tables): 3,083

For peer review only

Abstract

Introduction. Preterm birth is closely associated with altered brain development and is a leading cause of neurodevelopmental, cognitive and behavioural impairment across the life course. We aim to investigate neuroanatomic variation and adverse outcomes associated with preterm birth by studying a cohort of preterm infants and controls born at term, using brain magnetic resonance imaging (MRI) linked to biosamples and clinical, environmental and neuropsychological data.

Methods and Analysis. Theirworld Edinburgh Birth Cohort is a prospective longitudinal cohort study at the University of Edinburgh. We plan to recruit 300 infants born at <33 weeks gestational age (GA) and 100 healthy control infants born after 37 weeks GA. Multiple domains are assessed: maternal and infant clinical and demographic information; placental histology; immunoregulatory and trophic proteins in umbilical cord and neonatal blood; brain macro- and microstructure from structural and diffusion MRI; DNA methylation; hypothalamic-pituitary-adrenal axis (HPAA) activity; social cognition, attention and processing speed from eye-tracking during infancy and childhood; neurodevelopment; gut and respiratory microbiota; susceptibility to viral infections; and participant experience. Main analyses include creation of novel methods for extracting information from neonatal structural and diffusion MRI, regression analyses of predictors of brain maldevelopment and neurocognitive outcome associated with preterm birth, and determination of the quantitative predictive performance of MRI and other early life factors for childhood outcome.

Ethics and Dissemination. Ethical approval has been obtained from the National Research Ethics Service, South East Scotland Research Ethics Committee and NHS Lothian Research and Development. Results are disseminated through open access journals, scientific meetings, social media, newsletters, a study website (www.tebc.ed.ac.uk), and we engage with the University of Edinburgh public relations and media office to ensure maximum publicity and benefit.

For peer review only

Strengths and limitations of this study

- 300 preterm infants and a comparator cohort of 100 term controls studied longitudinally from before birth to school age.
- Deep phenotyping using a combination of data from brain MRI, biosamples, participant report, direct observation and clinical data from medical records.
- Collection of data about a range of theoretically informed variables to understand the wider impact of preterm birth on everyday lives of families.
- Data access and collaboration policy sets out the terms and conditions on which deidentified TEBC data is available to the research community.
- Participants are recruited from a single centre.

For peer review only

INTRODUCTION

Preterm delivery is estimated to affect 10.6% of all live births around the world, which equates to 14.84 million births per annum[1]. In resource rich settings advances in perinatal care and service delivery have led to improved survival over the past two decades: around 30% of infants born at 22 weeks who are offered stabilisation at birth will survive, and this number increases to around 80% for births at 26 weeks[2-5]. However, early exposure to extrauterine life can impact brain development, and is closely associated with long term intellectual disability, cerebral palsy, autism spectrum disorder, attention deficit hyperactivity disorder, psychiatric disease, and problems with language, behaviour, and socioemotional function (for review [6]). There are no treatments that reduce risk of impairment, which extends across the life course and carries considerable personal cost to affected individuals, and high health and education costs to society[7].

Little is known about the ontogenesis of neurocognitive and psychiatric problems associated with preterm birth, or the biological, environmental and social risk factors associated with susceptibility and resilience. Much information about the cerebral effects of preterm birth comes from historic cohorts that do not reflect modern perinatal care practices; studies have been cross-sectional with outcomes assessed in very early childhood before important cognitive and social functions emerge; conventional diagnostic tools for assessing neurodevelopment are imprecise; and cohorts linked to imaging and biological metadata are few so mechanisms are poorly understood. There is an unmet need to study a contemporary cohort of preterm infants that is comprehensively characterised from genes to anatomy to function, integrated with information about the social graph.

Our aims are: first, to build a longitudinal cohort of preterm infants and term controls that is phenotyped with brain imaging and biological information to investigate causal pathways to, and consequences of, atypical brain development and injury; second, to develop novel computational algorithms for mapping brain growth and connectivity in early life; third, to identify new and multi-factorial methods for early detection of children at risk of long-term impairment; and fourth, to identify early life biological and environmental risk and resilience factors that affect the developing brain and so pave the way for new therapeutic strategies.

For peer review only

METHODS AND ANALYSIS

Study design

Single-centre prospective longitudinal cohort study.

Study setting

The Theirworld Edinburgh Birth Cohort ("TEBC") study is conducted at the University of Edinburgh and the Simpson Centre for Reproductive Health (SCRH) which is located at the Royal Infirmary of Edinburgh, NHS Lothian, UK. The SCRH provides maternity and newborn services for residents of the City of Edinburgh and the Lothians. It receives 7,000 deliveries per annum and is the regional centre for all neonatal intensive care in South East Scotland. Approximately 100 infants with birthweight <1500g receive intensive care at SCRH per annum.

Participant recruitment, initial assessment and data collection points 1-3 (Table 1) take place in the SCRH or the Edinburgh Imaging Facility, Royal Infirmary of Edinburgh. Follow-up assessments take place in a dedicated child development laboratory at the University of Edinburgh, through online and in-person completion of questionnaires, and in Neonatal Out-patient clinics at the SCRH (timepoints 4-7, Table1). Recruitment began in November 2016 and is planned to complete in 2021.

Study participants

Inclusion criteria

Cases: 300 preterm infants born at <33 weeks gestational age (GA)*.

Controls: 100 term infants born at >37 weeks GA*.

*GA is estimated based on first trimester ultrasound.

Exclusion criteria

1. Infants with congenital anomalies: structural or functional anomalies (e.g. metabolic disorders) that occur during intrauterine life and can be identified prenatally, at birth or later in life (World Health Organisation definition).
2. Infants with a contraindication to MRI at 3 Tesla.

Sample selection and recruitment

Sample size

The primary objective of the study is to investigate causes and consequences of preterm brain injury / atypical development by analysing data about brain macro- and microstructure from structural and quantitative MRI with biological, environmental and neuropsychological

For peer review only

outcome data. There is no established methodology for power calculations using quantitative MRI techniques; sample size is based on sensitivity analysis for tract-based Spatial Statistics[8], and precedents for detecting group differences in neonatal structural and diffusion MRI (dMRI) based on exposures and outcomes [9-18]. It assumes a successful image acquisition rate of 85%.

Identifying participants

Cases: Infants born to women who present to the SCRH with threatened preterm labour and for whom delivery is planned or expected at less than 32 completed weeks GA.

Controls: Infants born to women who attend the SCRH and deliver at >36 weeks GA.

The protocol reported here was partially developed through a separate, pilot "phase 1' cohort of 150 cases and 40 controls. This phase 1 pilot included neonatal MRI and infant-eye-tracking, and a subset of this group are now participating in the 5-year assessment as described here (time point 7, table 1).

Screening for eligibility

The research nurse / clinical research fellow identifies potential participants using maternity TRAK, which is a system used by maternity services throughout NHS Lothian to record information about pregnancies and maternal care, and the neonatal electronic patient record. The clinical team provides an introductory leaflet about TEBC to eligible parents, and then informs the research team of parents who wish to discuss the study in greater detail. Those parents meet with a member of the research team and are provided with the Participant Information Sheet.

Participants from phase 1 studies being recalled for time point 7 (at 5 years) are contacted by the research team using contact details provided previously. Study information (introductory letter, patient information sheet, reply slip and prepaid envelope) is sent by post and followed up with a telephone call to answer any questions and review willingness to participate.

Consenting participants

Informed written consent is sought in two stages: first, consent for perinatal and neonatal sampling and assessment at initial enrolment to the study; second, consent for assessments post-discharge to 5 years is taken at time point 3 (see Table 1 below).

For phase 1 participants being recalled, consent is taken at the recall appointment, following circulation and discussion of the content by post and phone, as described above.

For peer review only

Informed consent may only be taken by a member of the research team with training in International Council for Harmonisation-Good Clinical Practice (ICH-GCP) and procedures for research involving children and young people.

Co-enrolment

The SCRH is an academic perinatal medicine centre that hosts observational research studies, and it is a recruiting centre for randomised controlled trials of therapies designed to improve the outcome of preterm infants and their mothers. Parents / carers of TEBC participants are encouraged to consider entry into such studies if eligible. Co-enrolment is informed by 'Guidelines for Co-enrolment' produced by the Academic and Clinical Central Office for Research and Development (ACCORD), which is a partnership between the University of Edinburgh and NHS Lothian Health Board. Co-enrolment will be recorded.

Cohort retention

Participants and their families are kept up to date with research progress through Newsletters, Twitter, Facebook and a website (www.tebc.ed.ac.uk). Birthday cards are sent to participants and we hold an annual event for research updates and public outreach.

Withdrawal of study participants

The decision to withdraw from the study is either at parental / carer request, or at the request of the attending consultant physician or the PI for clinical reasons.

Outcomes and data analysis

Table 1 summarises the assessment schedule, data collection methods, sample type / domain, and the test or task. Data from cases and controls are collected using the same data collection instruments.

For peer review only

Time point	Age	Data collection method	Sample type / domain of measurement	Test / task
1	Antenatal	Records & interview	Socio-economic status	Maternal & paternal education, Scottish Index of Multiple Deprivation derived from home postcode
			Medical / demographic	Family and medical history and exposures
2	Birth	Records, questionnaire & tissue	Medical	History and exposures
				Anthropometry
			Placenta	Structured histopathology rating and storage
			Cord blood	Panel of immunoregulatory and trophic proteins<br>Gene expression array*
3	Neonatal	Tissue: blood	Blood spot	Panel of immunoregulatory and trophic proteins<br>Gene expression array*
		Tissue: saliva	Epigenetics	DNA methylation
		Tissue: nasal swab	Nasal lining fluid	Antimicrobial peptides including cathelicidin levels*
			DNA/RNA	Respiratory microbiota*
		Stool	DNA/RNA	Gut microbiota*
		Direct observation	Medical	Anthropometry
			ROP assessment	Grade retinopathy
			Parent IQ	National Adult Reading Test
		MRI	Brain structure and connectivity	Structural and diffusion 3T MRI
		Questionnaire	Medical / demographic	Breast-feeding and updated perinatal medical history
				Edinburgh Post-natal Depression Scale
				Parenting Daily Hassles
				World Health Organisation – Quality Of Life
				Adult Temperament Questionnaire
4	4.5 months	Questionnaire, by post or online or phone interview	Demographics	Updated Socio-economic status, maternal education, breastfeeding / nutrition, activities
			Infant temperament	Infant Behaviour Questionnaire, Revised, short form
			Parent wellbeing	Edinburgh Post-natal Depression Scale
				World Health Organisation – Quality Of Life
		Tissue: nasal swab	DNA/RNA	Respiratory microbiota*
5	9 months	Tissue: saliva	Epigenetics	DNAm
			HPA axis	Cortisol: Waking, 30 minutes after waking, before bed.<br>Pre and post Still Face procedure
		Tissue: nasal swab	Nasal lining fluid	Antimicrobial peptides including cathelicidin levels*
			DNA/RNA	Respiratory microbiota*
		Eye-tracking	Social development	Free scanning: neutral faces
				Free scanning: “pop-out” task, looking to faces and distractors
				Free scanning: “social preferential looking” to social and non-social images

For peer review only

				Free scanning: “dancing ladies” social and non-social videos
			Attention	Switching and disengagement: “gap-overlap” task, fixation to central and peripheral cues
				Sustained attention: “follow the bird” task, following moving target
			Processing speed	Free scanning: odd-one-out visual search task (simple letters version)
				Free-scanning: word-picture matching task
		**Direct observation**	Visual acuity	Keeler card assessment
			Social development	Still Face procedure (sub-set with computational motor assessment)
				Parent-child play, for later behavioural coding: (sub-set with computational motor assessment)
		**Questionnaire**	Infant temperament	Infant Behaviour Questionnaire, Revised, short form
				Sleep & Settle Questionnaire
			Language	MacArthur Communicative Development Inventory (words and gestures)
			Parent wellbeing	World Health Organisation – Quality Of Life
			Feedback	Feedback form, monitoring satisfaction with research project
		**Direct observation**	Anthropometry	Growth
		**Parent interview**	Demographics	Family circumstances update form including breastfeeding, socio-economic status (home postcode)
			Developmental level	Vineland Adaptive Behaviour Scales: comprehensive interview form
**6**	**2 years**	**Direct observation**	Ophthalmology	Refraction
			Anthropometry	Growth
		**Tissue: nasal swab**	Nasal lining fluid	Antimicrobial peptides including cathelicidin levels*
			DNA/RNA	Respiratory microbiota*
		**Eye-tracking**	Social development	Free scanning: neutral faces
				Free scanning: “pop-out” task, looking to faces and distractors
				Free scanning: “social preferential looking” to social and non-social images
				Free scanning: “dancing ladies” social and non-social videos
			Attention	Switching and disengagement: “gap-overlap” task, fixation to central and peripheral cues
				Sustained attention: “follow the bird” task, following moving target
			Processing speed	Free scanning: odd-one-out visual search task
				Free-scanning: word-picture matching task
		**Direct observation**	Social development	Parent-child play, for later behavioural coding
			Executive function	Following Instructions task
			Bayley-III	General developmental level*
		**Questionnaire**	Temperament	Early Childhood Behaviour Questionnaire, Revised, short form
				Child Sleep Habits Questionnaire
			Language	MacArthur Communicative Development Inventory (words and sentences)
			Social development	Quantitative Checklist for Autism in Toddlers
			Executive function	Behaviour Rating Inventory for Executive Function, Pre-school (BRIEF-P)<br>Early Executive Function Questionnaire
			Developmental level	Vineland Adaptive Behaviour Scales: comprehensive parent rating form

For peer review only

For peer review only - http://bmjopen.bmj.com/site/about/guidelines.xhtml

			Parent wellbeing	World Health Organisation – Quality Of Life
			Feedback	Feedback form, monitoring satisfaction with research project
		**Parent interview**	Demographics	Family circumstances update form including breastfeeding, socio-economic status (home postcode)
**7**	**5 years**	**Tissue: saliva**	Epigenetics	DNA methylation
			HPA axis	Cortisol
		**Tissue: nasal swab**	DNA/RNA	Respiratory microbiota*
		**Direct observation**	Anthropometry	Growth
			Blood pressure	Hypertension
			Ophthalmology	Refraction and acuity
			Social development	Parent-child play, for later behavioural coding
			Executive function	Following Instructions task
			Developmental level	Mullen Scales of Early Learning
		**Eye-tracking**	Social development	Free scanning: neutral faces
				Free scanning: “pop-out” task, looking to faces and distractors
				Free scanning: “social preferential looking” to social and non-social images
				Free scanning: “dancing ladies” social and non-social videos
			Attention	Switching and disengagement: “gap-overlap” task, fixation to central and peripheral cues
				Sustained attention: “follow the bird” task, following moving target
			Processing speed	Free scanning: odd-one-out visual search task (complex objects version)
		**Questionnaire**	Temperament	Strengths and Difficulties Questionnaire (both teacher and parent report versions)
			Language	Children's Communication Checklist
			Social development	Social Communication Questionnaire: Current
			Executive function	DUPaul ADHD rating scale
				Behaviour Rating Inventory for Executive Function -Pre-school (BRIEF-P)
			Visual perception	Cerebral Visual Impairment Inventory
			Parent wellbeing	World Health Organisation – Quality Of Life
			Feedback	Feedback form monitoring satisfaction with research project
			Developmental level	Vineland Adaptive Behaviour Scales: domain-level parent rating form
		**Parent interview**	Demographics	Family circumstances update form including socio-economic status (home postcode)

Table 1. Schedule of assessments, data collection methods, sample type / domain, and the test or task.
*subset of participants

For peer review only

Maternal and infant clinical and demographic information

Data are abstracted from the mothers' and infants' electronic medical records onto a standardised data collection sheet. A structured maternal interview is used to collect additional information that may not be recorded in routinely collected data, for example detailed family history about neurodevelopmental and mental health problems, and over-the-counter prescription and recreational drugs taken during pregnancy. For deaths the cause and post-mortem findings will be recorded.

Placentas

After delivery, placentae from all preterm infants are formalin fixed and stored at 4°C before sampling. The placentae are sampled according to a standardized protocol; distal and proximal sections of cord (the proximal section being taken at 1.5 cm from above the fetal surface), a roll of extraplacental membranes starting at the point of rupture and 4 full thickness sections from each quadrant. All are stained with Haematoxylin and Eosin and reported using a standardised, structured approach that describes any pathological features present, including but not limited to, fetal thrombotic vasculopathy, villitis, chorioamnionitis, funisitis and features of uteroplacental ischaemia[19 20].

Immunoregulatory and trophic proteins

Analysis of a panel of immunoregulatory and trophic proteins (IL-1b, IL-2, IL-4, IL-5, IL-6, IL-8, IL-12, IL-17, TNF-a, MIP-1b, BDNF, GM-CSF, IL-10, IL-18, IFN-g, TNF-b, MCP-1, MIP-1a, C3, C5a, C9, MMP-9, RANTES and CRP) is undertaken on umbilical cord and neonatal blood samples. These proteins are selected to offer information with respect to the pro- and anti-inflammatory innate response as well as the adaptive immune response. Blood is collected using Schleicher and Schuell 903 filter paper (6 x 3.2mm spots per subject) and analysed using a multiplex immunoassay (Meso Scale Discovery) at Statens Serum Institute, Copenhagen. We use the approach described by Skögstrand et al[21] to analyse differences in concentration between cases and controls.

Structural and diffusion magnetic resonance imaging

A Siemens MAGNETOM Prisma 3T MRI clinical scanner (Siemens Healthcare, Erlangen, Germany) and 16-channel phased-array paediatric head receive coil is used to acquire: 3D T1-weighted MPRAGE (T1w) structural volume scan (acquired voxel size = 1 mm isotropic) with TI 1100 ms, TE 4.69 ms and TR 1970 ms; a 3D T2-weighted SPACE (T2w) structural scan (voxel size = 1mm isotropic) with TE 409 ms and TR 3200 ms; and a multi-shell axial dMRI scan (16 ×

For peer review only - http://bmjopen.bmj.com/site/about/guidelines.xhtml

b = 0 s/mm^2, 3 × b = 200 s/mm^2, 6 × b = 500 s/mm^2, 64 × b = 750 s/mm^2, 64 × b = 2500 s/mm^2) with optimal angular coverage[22] (see Supplementary material 1-3). If the infant stays settled axial 3D susceptibility weighted imaging (SWI; TR = 28 ms, TE = 20 ms, 0.75 x 0.75 x 3 mm acquired resolution) and axial 2D fluid-attenuated inversion-recovery BLADE imaging (FLAIR; TR = 10000 ms, TE = 130 ms, TI = 2606 ms, 0.94 x 0.94 x 3 mm acquired resolution) are acquired. In a subgroup of participants magnetisation transfer saturation imaging is acquired for evaluation of tissue myelin content, consisting of three sagittal 3D multi-echo spoiled gradient echo scans (TE = {1.54 ms, 4.55 ms, 8.56 ms}, 2-mm isotropic acquired resolution): magnetisation-transfer and proton-density weighted (TR = 75 ms, FA = 5°), and T1-weighted (TR = 15 ms, FA = 14°) acquisitions, supplementary material 4. Tissue heating and acoustic noise exposure are limited throughout the examination through the use of active noise cancellation and by setting the gradient slew rate and other pulse sequence parameters appropriately. Participants are scanned in normal mode with respect to both tissue heating and peripheral nerve stimulation.

Conventional images are reported by a paediatric radiologist using a structured system [18, 23]. We use image data to generate novel processing techniques optimised for neonatal data[15 24-27], and we will use these and other publicly available pipelines for processing neonatal data[28-30] to derive image features for analyses with collateral data relating to exposures and outcomes. These include but are not limited to tract-based, morphometric and structural connectivity analyses [13 16 20 24 31-34].

DNA storage

DNA is extracted form saliva, stored and catalogued at the Edinburgh Clinical Research Facility, ready for downstream analyses.

DNA methylation

Saliva is sampled using the DNA OG-575 kit (DNAGenotek, Ottawa, ON, Canada). DNA extraction is performed using published methods[16] and DNAm analyses are carried out at the Genetics Core of the Edinburgh Clinical Research Facility (Edinburgh, UK), using Illumina Infinium MethylationEPIC (San Diego, CA, USA), with interrogation of the arrays against ~850k methylation sites. We will investigate perinatal influences on DNAm using principal component analysis, mediation, and correlation analyses.

For peer review only

Hypothalamic-pituitary-adrenal axis (HPAA)

Salivary cortisol is used as a marker of HPAA activity. Saliva is collected in Sarstedt tubes at specified times at 9 months and 5 years. Timed saliva samples are also collected during the 9 months appointment before and after a behavioural paradigm (Still Face) which is known to elicit a biological stress response (one sample pretest and two samples post test to capture reaction and recovery). Samples are stored at -20C and analysed in batches at each time point. Anthropometric data are recorded at 9 months, 2 years and 5 years, and blood pressure is measured at 5 years.

Eye-tracking

We record eye-movements in response to visual stimuli at 9 months, 2 years and 5 years using a Tobii© x60 eye-tracker and bespoke analysis software (Matlab). Images are presented on a display monitor with a resolution of 1,440 × 900 pixels. The Tobii© ×60 system tracks both eyes to a rated accuracy of 0.3 degrees at a rate of 60 Hz. We analyse looking patterns, including time to first fixate and looking time at areas of interest, in tasks designed to enable inference about social development, attention, and processing speed[31 35].

Standardised assessments

Standardised assessments of neurodevelopment by direct observation at appropriate time points are: Bayley-III scales; Mullen Scales of Early Learning; parental IQ (National Adult Reading Test). We will use validated questionnaires to assess: infant/parent temperament; parent/family characteristics (postnatal depression, stress, quality of life, socioeconomic status); infant / child sleep habits; language development; social development; executive functions; cerebral visual impairment; medical diagnoses; and behavioural outcomes (parent and teacher ratings). We also record parent-child interaction for subsequent analysis via video coding of complex behaviours in a naturalistic context.

Susceptibility to viral infection

We collect unstimulated nasal secretion samples (nasosorption samples) using methods described by Thwaites et al[36]. This collection is brief, minimally invasive and a minimally distressing process. Nasosorption Nasal lining fluid is collected using Nasosorption Fxi synthetic absorption matrix strips inserted into the anterior part of the inferior turbinate of the nasal cavity. After 30 seconds of absorption, the strip is removed, capped, maintained at 4°C for up to 4 hours and then frozen at −80°C. From these nasal fluid samples we will assess the levels of antimicrobial peptides, including cathelicidin, and inflammatory cytokines, by

For peer review only

ELISA or luminex assay. Collection of these at birth (term equivalent age), 9 months and 2 years will enable us to characterise birth levels, levels at timepoints significant for respiratory syncytial virus (RSV) infection/disease and at a later time point.

Respiratory and gut microbiota

We collect faecal and nasopharyngeal swabs (paediatric Copan e-swab with flocked nylon fiber tip) as has been described in the WHO-guideline for respiratory sampling of bacterial pathogens[66]. Fecal material and e-swabs (in RNA protect), are frozen at -80°C until further analyses. DNA and RNA will be extracted[37] and metagenomics analyses will be executed by 16S-based sequencing according to previously described methods[38]. We will study temporal relationships between preterm birth and early life characteristics, consecutive microbiota development, inflammation and methylation findings, and respiratory and neurocognitive developmental outcomes.

Computational Motor Assessment

Light-weight, wearable, wireless motion sensors are deployed to record the movement of a sub-set of infants at 9 months during the Still-Face paradigm and Parent-Child interaction. Data are anonymised before being securely transferred to the University of Strathclyde for analysis. These data will be analysed to test for differences in motor function between at-risk and low-risk infants, and will employ machine learning algorithms to detect patterns predictive of developmental outcome at 2 and 5 years, and their potential for clinical stratification across the neurodevelopmental disorders and psychometric profiles (IQ, adaptive function, language). Further, motor data at 9 months can be correlated against neuroanatomical features measured by MRI scan at birth and developmental scales at 9 months.

Patient and Public Involvement

We seek feedback from parents / carers to monitor satisfaction with research participation at 9 months, 2 years and 5 years, and we have a public facing website that describes results from the study.

ETHICS AND DISSEMINATION

Safety assessment

For peer review only

There are no safety issues associated with collection of: placental tissue, umbilical cord / neonatal blood, saliva, faeces or hair. There are no safety issues in the conduct of planned neuropsychological assessments.

MRI does not involve ionizing radiation and there are no known risks from MRI provided standard safety measures for 3T scanning are in place. Infants are fed and wrapped and allowed to sleep naturally in the scanner. Pulse oximetry, electrocardiography and temperature are monitored. Flexible earplugs and neonatal earmuffs (MiniMuffs, Natus) are used for acoustic protection. All scans are supervised by a doctor or nurse trained in neonatal resuscitation. The scan is interrupted if there are any abnormalities in monitoring or if the baby wakes.

It is possible that incidental findings may be found on MRI or from questionnaires, for example intracranial structural anomalies or postnatal depression, respectively. In these circumstances, the findings are discussed with the participant's parent, and referral to the appropriate NHS service is made.

Ethical approvals

The study has been approved by the National Research Ethics Service (South East Scotland Research Ethics Committee), NRES numbers 11/55/0061 and 13/SS/0143 (Phase 1) and NRES number 16/SS/0154 (Phase 2); and by NHS Lothian Research & Development (2016/0255).

Governance

The study is run by a management group that includes the principal investigator, a minimum of two co-investigators, the study coordinator and administrative and financial officers. A delegation log details the responsibilities of each member of staff working on the study. A scientific advisory board oversees the conduct and progress of the study. The study is co-sponsored by the University of Edinburgh & NHS Lothian Academic and Clinical Central Office for Research and Development (ACCORD).

Publication and data statement

The principles set down by the International Committee of Medical Journal Editors for authorship and non-author contributors are followed for publications and presentations resulting from the study. A Data Access and Collaboration Policy sets out the terms and conditions on which deidentified TEBC data, stimuli and tasks are accessible to the research community following reasonable request (www.tebc.ed.ac.uk).

For peer review only

SUMMARY

The aim of TEBC is to recruit a longitudinal cohort of 300 preterm infants and 100 term controls and to acquire brain MRI data that are linked to comprehensive biosampling and detailed clinical, environmental and neuropsychological data.

Data from TEBC will be used to:

- develop novel image processing algorithms for mapping brain growth and connectivity in early life;
- identify biological and environmental exposures that modify brain development;
- deepen understanding of the complex interaction between perinatal events and later environmental influences on brain health and outcome after preterm birth;
- develop methods for early detection of risk and resilience factors for long-term outcome.

Author contributions

JPB designed the study with input from all the authors. JPB, JH, MJT, RMR, SC, JS, DB, DJD, AJD, MEB and SF-W contributed to the establishment and refinement of study procedures and critically revised the manuscript. All authors approved the final version of the manuscript.

Competing interests

None declared.

Acknowledgements

The authors would like to thank participating families, and NHS colleagues at the Simpson Centre for Reproductive Health who support this study. We would like to thank Mrs Bavanthe Navarathne, a parent representative on the scientific advisory board, and other past and present members of the scientific advisory board (Frances Cowan, Chiara Nosarti, David Porteous, Hugh Rabagliati, Joanna Wardlaw, Heather Whalley). We are grateful to the following collaborators, colleagues and students who support the study: Gayle Barclay, Justyna Binkowska, Gillian Black, Manuel Blesa, Nis Borbye-Lorenzen, Geoff Carlson, Yu Wei Chua, Simon Cox, Hilary Cruikshank, Bethan Dean, Jonathan Delafield-Butt, Fiona Denison, Margaret Evans, Paola Galdi, Peter Ghazal, Lorna Ginnell, Charlotte Jardine, Gillian Lamb, Victoria Ledsham, Riccardo Marioni, Andrew McIntosh, Barbara Nugent, Lee Murphy, Sinéad O'Carroll, Alan Quigley, Alan Mulvihill, Magda Rudnicka, Scott Semple, Kristin Skögstrand,

For peer review only

Sarah Stock, David Stoye, Gemma Sullivan, Kadi Vaher, colleagues at the Genetics Core of the Edinburgh Clinical Research Facility, and radiographers at the Edinburgh Imaging Facility Royal Infirmary of Edinburgh.

Funding statement

The TEBC study is funded by the charity Theirworld (www.theirworld.org) and is carried out in the University of Edinburgh MRC Centre for Reproductive Health (MRC G1002033). Susceptibility to viral infection studies are supported by grants from Action Medical Research (GN2703) and Chief Scientist Office (TCS/18/02). Respiratory microbiota studies are supported by grants from the Chief Scientist Office (SCAF/16/03), and DNA methylation and gut microbiota studies are supported by the Wellcome Trust (203769/Z/16/A and 220043/Z/19/Z). The MRI facility is funded by Wellcome Trust 104916/Z/14/Z, Dunhill Trust R380R/1114, Edinburgh and Lothians Health Foundation 2012/17, Muir Maxwell Research Fund, and Edinburgh Imaging, University of Edinburgh. MJT was supported by NHS Lothian Research and Development Office, and RMR and AJD receive support from the British Heart Foundation (RE/18/5/34216).

For peer review only

REFERENCES

1. Chawanpaiboon S, Vogel JP, Moller AB, et al. Global, regional, and national estimates of levels of preterm birth in 2014: a systematic review and modelling analysis. *The Lancet Global health* 2019;7(1):e37-e46. doi: 10.1016/s2214-109x(18)30451-0 [published Online First: 2018/11/06]
2. Mehler K, Oberthuer A, Keller T, et al. Survival Among Infants Born at 22 or 23 Weeks' Gestation Following Active Prenatal and Postnatal Care. *JAMA pediatrics* 2016;170(7):671-7. doi: 10.1001/jamapediatrics.2016.0207 [published Online First: 2016/05/24]
3. Patel RM, Rysavy MA, Bell EF, et al. Survival of Infants Born at Periviable Gestational Ages. *Clinics in perinatology* 2017;44(2):287-303. doi: 10.1016/j.clp.2017.01.009 [published Online First: 2017/05/10]
4. Norman M, Hallberg B, Abrahamsson T, et al. Association Between Year of Birth and 1-Year Survival Among Extremely Preterm Infants in Sweden During 2004-2007 and 2014-2016. *Jama* 2019;321(12):1188-99. doi: 10.1001/jama.2019.2021 [published Online First: 2019/03/27]
5. Myrhaug HT, Brurberg KG, Hov L, et al. Survival and Impairment of Extremely Premature Infants: A Meta-analysis. *Pediatrics* 2019;143(2) doi: 10.1542/peds.2018-0933 [published Online First: 2019/02/02]
6. Johnson S, Marlow N. Early and long-term outcome of infants born extremely preterm. *Archives of disease in childhood* 2017;102(1):97-102. doi: 10.1136/archdischild-2015-309581 [published Online First: 2016/08/12]
7. Mangham LJ, Petrou S, Doyle LW, et al. The cost of preterm birth throughout childhood in England and Wales. *Pediatrics* 2009;123(2):e312-e27.
8. Ball G, Boardman JP, Arichi T, et al. Testing the sensitivity of tract-based spatial statistics to simulated treatment effects in preterm neonates. *PLoSOne* 2013;8(7):e67706.
9. Peterson BS, Vohr B, Staib LH, et al. Regional brain volume abnormalities and long-term cognitive outcome in preterm infants. *JAMA* 2000;284(15):1939-47.
10. Boardman JP, Counsell SJ, Rueckert D, et al. Early growth in brain volume is preserved in the majority of preterm infants. *AnnNeurol* 2007;62(2):185-92.
11. Boardman JP, Craven C, Valappil S, et al. A common neonatal image phenotype predicts adverse neurodevelopmental outcome in children born preterm. *Neuroimage* 2010;52(2):409-14.
12. Ball G, Boardman JP, Aljabar P, et al. The influence of preterm birth on the developing thalamocortical connectome. *Cortex* 2012
13. Boardman JP, Walley A, Ball G, et al. Common genetic variants and risk of brain injury after preterm birth. *Pediatrics* 2014;133(6):e1655-63. doi: 10.1542/peds.2013-3011 [published Online First: 2014/05/14]
14. Ball G, Aljabar P, Zebari S, et al. Rich-club organization of the newborn human brain. *Proceedings of the National Academy of Sciences of the United States of America* 2014;111(20):7456-61. doi: 10.1073/pnas.1324118111 [published Online First: 2014/05/07]
15. Anblagan D, Bastin ME, Sparrow S, et al. Tract shape modeling detects changes associated with preterm birth and neuroprotective treatment effects. *NeuroImage: Clinical* 2015;8:51-58. doi: doi:10.1016/j.nicl.2015.03.021
16. Sparrow S, Manning JR, Cartier J, et al. Epigenomic profiling of preterm infants reveals DNA methylation differences at sites associated with neural function. *Translational*

For peer review only

psychiatry 2016;6:e716. doi: 10.1038/tp.2015.210 [published Online First: 2016/01/20]

17. Kersbergen KJ, de Vries LS, van Kooij BJ, et al. Hydrocortisone treatment for bronchopulmonary dysplasia and brain volumes in preterm infants. *J Pediatr* 2013;163(3):666-71.e1. doi: 10.1016/j.jpeds.2013.04.001 [published Online First: 2013/05/28]
18. Woodward LJ, Anderson PJ, Austin NC, et al. Neonatal MRI to predict neurodevelopmental outcomes in preterm infants. *NEnglJ Med* 2006;355(7):685-94.
19. Redline RW, Faye-Petersen O, Heller D, et al. Amniotic infection syndrome: nosology and reproducibility of placental reaction patterns. *Pediatric and developmental pathology : the official journal of the Society for Pediatric Pathology and the Paediatric Pathology Society* 2003;6(5):435-48. doi: 10.1007/s10024-003-7070-y [published Online First: 2004/01/08]
20. Anblagan D, Pataky R, Evans MJ, et al. Association between preterm brain injury and exposure to chorioamnionitis during fetal life. *Scientific reports* 2016;6:37932. doi: 10.1038/srep37932 [published Online First: 2016/12/03]
21. Skogstrand K, Thorsen P, Norgaard-Pedersen B, et al. Simultaneous measurement of 25 inflammatory markers and neurotrophins in neonatal dried blood spots by immunoassay with xMAP technology. *Clinical chemistry* 2005;51(10):1854-66. doi: 10.1373/clinchem.2005.052241 [published Online First: 2005/08/06]
22. Caruyer E, Lenglet C, Sapiro G, et al. Design of multishell sampling schemes with uniform coverage in diffusion MRI. *Magnetic resonance in medicine* 2013;69(6):1534-40. doi: 10.1002/mrm.24736 [published Online First: 2013/04/30]
23. Leuchter RH, Gui L, Poncet A, et al. Association between early administration of high-dose erythropoietin in preterm infants and brain MRI abnormality at term-equivalent age. *Jama* 2014;312(8):817-24. doi: 10.1001/jama.2014.9645 [published Online First: 2014/08/27]
24. Blesa M, Sullivan G, Anblagan D, et al. Early breast milk exposure modifies brain connectivity in preterm infants. *NeuroImage* 2019;184:431-39. doi: 10.1016/j.neuroimage.2018.09.045 [published Online First: 2018/09/22]
25. Blesa M, Serag A, Wilkinson AG, et al. Parcellation of the Healthy Neonatal Brain into 107 Regions Using Atlas Propagation through Intermediate Time Points in Childhood. *Frontiers in neuroscience* 2016;10:220. doi: 10.3389/fnins.2016.00220 [published Online First: 2016/06/01]
26. Serag A, Blesa M, Moore EJ, et al. Accurate Learning with Few Atlases (ALFA): an algorithm for MRI neonatal brain extraction and comparison with 11 publicly available methods. *Scientific reports* 2016;6:23470. doi: 10.1038/srep23470 [published Online First: 2016/03/25]
27. Serag A, Wilkinson AG, Telford EJ, et al. SEGMA: An Automatic SEGMentation Approach for Human Brain MRI Using Sliding Window and Random Forests. *Frontiers in neuroinformatics* 2017;11:2. doi: 10.3389/fninf.2017.00002 [published Online First: 2017/02/07]
28. Ball G, Counsell SJ, Anjari M, et al. An optimised tract-based spatial statistics protocol for neonates: applications to prematurity and chronic lung disease. *Neuroimage* 2010;53(1):94-102.
29. Smith SM, Jenkinson M, Johansen-Berg H, et al. Tract-based spatial statistics: voxelwise analysis of multi-subject diffusion data. *Neuroimage* 2006;31(4):1487-505.

For peer review only

30. Makropoulos A, Robinson EC, Schuh A, et al. The developing human connectome project: A minimal processing pipeline for neonatal cortical surface reconstruction. *NeuroImage* 2018;173:88-112. doi: 10.1016/j.neuroimage.2018.01.054 [published Online First: 2018/02/08]
31. Telford EJ, Fletcher-Watson S, Gillespie-Smith K, et al. Preterm birth is associated with atypical social orienting in infancy detected using eye tracking. *Journal of child psychology and psychiatry, and allied disciplines* 2016;57(7):861-8. doi: 10.1111/jcpp.12546 [published Online First: 2016/03/05]
32. Krishnan ML, Van Steenwinckel J, Schang AL, et al. Integrative genomics of microglia implicates DLG4 (PSD95) in the white matter development of preterm infants. *Nature communications* 2017;8(1):428. doi: 10.1038/s41467-017-00422-w [published Online First: 2017/09/07]
33. Telford EJ, Cox SR, Fletcher-Watson S, et al. A latent measure explains substantial variance in white matter microstructure across the newborn human brain. *Brain structure & function* 2017;222(9):4023-33. doi: 10.1007/s00429-017-1455-6 [published Online First: 2017/06/08]
34. Monnelly VJ, Anblagan D, Quigley A, et al. Prenatal methadone exposure is associated with altered neonatal brain development. *NeuroImage Clinical* 2018;18:9-14. doi: 10.1016/j.nicl.2017.12.033 [published Online First: 2018/01/13]
35. Gillespie-Smith K, Boardman JP, Murray IC, et al. Multiple Measures of Fixation on Social Content in Infancy: Evidence for a Single Social Cognitive Construct? *Infancy* 2016;21(2):241-57. doi: 10.1111/infa.12103 [published Online First: 2016/03/08]
36. Thwaites RS, Jarvis HC, Singh N, et al. Absorption of Nasal and Bronchial Fluids: Precision Sampling of the Human Respiratory Mucosa and Laboratory Processing of Samples. *Journal of visualized experiments : JoVE* 2018(131) doi: 10.3791/56413 [published Online First: 2018/02/15]
37. de Steenhuijsen Piters WA, Heinonen S, Hasrat R, et al. Nasopharyngeal Microbiota, Host Transcriptome, and Disease Severity in Children with Respiratory Syncytial Virus Infection. *American journal of respiratory and critical care medicine* 2016;194(9):1104-15. doi: 10.1164/rccm.201602-0220OC [published Online First: 2016/11/01]
38. Bosch A, de Steenhuijsen Piters WAA, van Houten MA, et al. Maturation of the Infant Respiratory Microbiota, Environmental Drivers, and Health Consequences. A Prospective Cohort Study. *American journal of respiratory and critical care medicine* 2017;196(12):1582-90. doi: 10.1164/rccm.201703-0554OC [published Online First: 2017/07/01]

For peer review only

SIEMENS MAGNETOM Prisma

Table of contents

\\Study Protocols
BRAIN
Neonates
Theirworld - E161723
localizer_neonate
t2_haste_localiser
t2_blade_v3
t2_space_sag_p4_iso_v2x
DTI_Neonate_v6b_dummy
DTI_Neonate_v6b_rev
DTI_Neonate_v6b_pt1
DTI_Neonate_v6b_pt2
MPRAGE-v4
SWI_v2
t2_blade_dark-fluid_tra_v3

For peer review only

For peer review only - http://bmjopen.bmj.com/site/about/guidelines.xhtml

SIEMENS MAGNETOM Prisma

\\Study Protocols\BRAIN\Neonates\Theirworld - E161723\localizer_neonate

TA: 0:12 PM: REF Voxel size: 0.5×0.5×7.0 mmPAT: Off Rel. SNR: 1.00 : fl

Properties

Prio recon	On
Load images to viewer	On
Inline movie	Off
Auto store images	On
Load images to stamp segments	On
Load images to graphic segments	On
Auto open inline display	Off
Auto close inline display	Off
Start measurement without further preparation	Off
Wait for user to start	Off
Start measurements	Single measurement

Routine

Slice group	1
Slices	1
Dist. factor	20 %
Position	L0.0 P47.8 F62.3 mm
Orientation	Sagittal
Phase enc. dir.	A >> P
Slice group	2
Slices	1
Dist. factor	20 %
Position	L0.0 P47.8 F62.3 mm
Orientation	Transversal
Phase enc. dir.	A >> P
Slice group	3
Slices	1
Dist. factor	20 %
Position	L0.0 P47.8 F62.3 mm
Orientation	Coronal
Phase enc. dir.	R >> L
AutoAlign	---
Phase oversampling	0 %
FoV read	250 mm
FoV phase	100.0 %
Slice thickness	7.0 mm
TR	7.5 ms
TE	3.69 ms
Averages	2
Concatenations	3
Filter	Prescan Normalize, Elliptical filter
Coil elements	PeH;PeN

Contrast - Common

TR	7.5 ms
TE	3.69 ms
TD	0 ms
MTC	Off
Magn. preparation	None
Flip angle	20 deg
Fat suppr.	None
Water suppr.	None
SWI	Off

Contrast - Dynamic

Averages	2
Averaging mode	Short term
Reconstruction	Magnitude
Measurements	1

Contrast - Dynamic

Multiple series	Each measurement

Resolution - Common

FoV read	250 mm
FoV phase	100.0 %
Slice thickness	7.0 mm
Base resolution	256
Phase resolution	91 %
Phase partial Fourier	Off
Interpolation	On

Resolution - iPAT

PAT mode	None

Resolution - Filter Image

Image Filter	Off
Distortion Corr.	Off
Prescan Normalize	On
Unfiltered images	Off
Normalize	Off
B1 filter	Off

Resolution - Filter Rawdata

Raw filter	Off
Elliptical filter	On

Geometry - Common

Slice group	1
Slices	1
Dist. factor	20 %
Position	L0.0 P47.8 F62.3 mm
Orientation	Sagittal
Phase enc. dir.	A >> P
Slice group	2
Slices	1
Dist. factor	20 %
Position	L0.0 P47.8 F62.3 mm
Orientation	Transversal
Phase enc. dir.	A >> P
Slice group	3
Slices	1
Dist. factor	20 %
Position	L0.0 P47.8 F62.3 mm
Orientation	Coronal
Phase enc. dir.	R >> L
FoV read	250 mm
FoV phase	100.0 %
Slice thickness	7.0 mm
TR	7.5 ms
Multi-slice mode	Sequential
Series	Interleaved
Concatenations	3

Geometry - AutoAlign

Slice group	1
Position	L0.0 P47.8 F62.3 mm
Orientation	Sagittal
Phase enc. dir.	A >> P
Slice group	2
Position	L0.0 P47.8 F62.3 mm

For peer review only

SIEMENS MAGNETOM Prisma

Geometry - AutoAlign

Orientation	Transversal
Phase enc. dir.	A >> P
Slice group	3
Position	L0.0 P47.8 F62.3 mm
Orientation	Coronal
Phase enc. dir.	R >> L
AutoAlign	---
Initial Position	L0.0 P47.8 F62.3
L	0.0 mm
P	47.8 mm
F	62.3 mm
Initial Rotation	0.00 deg
Initial Orientation	Sagittal

Geometry - Saturation

Saturation mode	Standard
Fat suppr.	None
Water suppr.	None
Special sat.	None

Geometry - Tim Planning Suite

Set-n-Go Protocol	Off
Table position	H
Table position	0 mm
Inline Composing	Off

System - Miscellaneous

Positioning mode	REF
Table position	H
Table position	0 mm
MSMA	S - C - T
Sagittal	R >> L
Coronal	A >> P
Transversal	F >> H
Coil Combine Mode	Adaptive Combine
Save uncombined	Off
Matrix Optimization	Off
AutoAlign	---
Coil Select Mode	Default

System - Adjustments

B0 Shim mode	Tune up
B1 Shim mode	TrueForm
Adjust with body coil	Off
Confirm freq. adjustment	Off
Assume Dominant Fat	Off
Assume Silicone	Off
Adjustment Tolerance	Auto

System - Adjust Volume

Position	Isocenter
Orientation	Transversal
Rotation	0.00 deg
A >> P	263 mm
R >> L	350 mm
F >> H	350 mm
Reset	Off

System - pTx Volumes

B1 Shim mode	TrueForm
Excitation	Slice-sel.

System - Tx/Rx

Frequency 1H	123.244318 MHz
Correction factor	1
Gain	High
Img. Scale Cor.	1.000
Reset	Off
? Ref. amplitude 1H	0.000 V

Physio - Signal1

1st Signal/Mode	None
TR	7.5 ms
Concatenations	3
Segments	1

Physio - Cardiac

Tagging	None
Magn. preparation	None
Fat suppr.	None
Dark blood	Off
FoV read	250 mm
FoV phase	100.0 %
Phase resolution	91 %

Physio - PACE

Resp. control	Off
Concatenations	3

Inline - Common

Subtract	Off
Measurements	1
StdDev	Off
Liver registration	Off
Save original images	On

Inline - MIP

MIP-Sag	Off
MIP-Cor	Off
MIP-Tra	Off
MIP-Time	Off
Save original images	On

Inline - Soft Tissue

Wash - In	Off
Wash - Out	Off
TTP	Off
PEI	Off
MIP - time	Off
Measurements	1

Inline - Composing

Inline Composing	Off
Distortion Corr.	Off

Inline - MapIt

Save original images	On
MapIt	None
Flip angle	20 deg
Measurements	1
Contrasts	1
TR	7.5 ms
TE	3.69 ms

Sequence - Part 1

Introduction	On

For peer review only - http://bmjopen.bmj.com/site/about/guidelines.xhtml

SIEMENS MAGNETOM Prisma

Sequence - Part 1

Dimension	2D
Phase stabilisation	Off
Asymmetric echo	Allowed
Contrasts	1
Flow comp.	No
Multi-slice mode	Sequential
Bandwidth	320 Hz/Px

Sequence - Part 2

Segments	1
Acoustic noise reduction	None
RF pulse type	Fast
Gradient mode	Fast
Excitation	Slice-sel.
RF spoiling	On

Sequence - Assistant

Mode	Off
Allowed delay	0 s

For peer review only

SIEMENS MAGNETOM Prisma

\\Study Protocols\BRAIN\Neonates\Theirworld - E161723\t2_haste_localiser
TA: 6.0 s PM: REF Voxel size: 0.7×0.7×4.0 mmPAT: 2 Rel. SNR: 1.00 : h

Properties

Prio recon	Off
Load images to viewer	On
Inline movie	Off
Auto store images	On
Load images to stamp segments	On
Load images to graphic segments	On
Auto open inline display	Off
Auto close inline display	Off
Start measurement without further preparation	Off
Wait for user to start	Off
Start measurements	Single measurement

Routine

Slice group	1
Slices	1
Dist. factor	30 %
Position	Isocenter
Orientation	Sagittal
Phase enc. dir.	A >> P
Slice group	2
Slices	1
Dist. factor	30 %
Position	L0.0 P0.0 H5.2 mm
Orientation	Transversal
Phase enc. dir.	R >> L
Slice group	3
Slices	1
Dist. factor	30 %
Position	L0.0 P0.0 H10.4 mm
Orientation	Coronal
Phase enc. dir.	R >> L
AutoAlign	---
Phase oversampling	0 %
FoV read	220 mm
FoV phase	100.0 %
Slice thickness	4.0 mm
TR	1500.0 ms
TE	94 ms
Averages	1
Concatenations	1
Filter	Prescan Normalize, Elliptical filter
Coil elements	HE1-4

Contrast - Common

TR	1500.0 ms
TE	94 ms
MTC	Off
Magn. preparation	None
Flip angle	150 deg
Fat suppr.	None
Water suppr.	None
Restore magn.	Off

Contrast - Dynamic

Averages	1
Averaging mode	Long term
Reconstruction	Magnitude
Measurements	1
Multiple series	Each measurement

Resolution - Common

FoV read	220 mm
FoV phase	100.0 %
Slice thickness	4.0 mm
Base resolution	320
Phase resolution	80 %
Phase partial Fourier	4/8
Interpolation	Off

Resolution - iPAT

PAT mode	GRAPPA
Accel. factor PE	2
Ref. lines PE	24
Reference scan mode	Integrated

Resolution - Filter Image

Image Filter	Off
Distortion Corr.	Off
Prescan Normalize	On
Unfiltered images	Off
Normalize	Off
B1 filter	Off

Resolution - Filter Rawdata

Raw filter	Off
Elliptical filter	On

Geometry - Common

Slice group	1
Slices	1
Dist. factor	30 %
Position	Isocenter
Orientation	Sagittal
Phase enc. dir.	A >> P
Slice group	2
Slices	1
Dist. factor	30 %
Position	L0.0 P0.0 H5.2 mm
Orientation	Transversal
Phase enc. dir.	R >> L
Slice group	3
Slices	1
Dist. factor	30 %
Position	L0.0 P0.0 H10.4 mm
Orientation	Coronal
Phase enc. dir.	R >> L
FoV read	220 mm
FoV phase	100.0 %
Slice thickness	4.0 mm
TR	1500.0 ms
Multi-slice mode	Single shot
Series	Interleaved
Concatenations	1

Geometry - AutoAlign

Slice group	1
Position	Isocenter
Orientation	Sagittal
Phase enc. dir.	A >> P
Slice group	2
Position	L0.0 P0.0 H5.2 mm

For peer review only - http://bmjopen.bmj.com/site/about/guidelines.xhtml

SIEMENS MAGNETOM Prisma

Geometry - AutoAlign

Orientation	Transversal
Phase enc. dir.	R >> L
Slice group	3
Position	L0.0 P0.0 H10.4 mm
Orientation	Coronal
Phase enc. dir.	R >> L
AutoAlign	---
Initial Position	Isocenter
L	0.0 mm
P	0.0 mm
H	0.0 mm
Initial Rotation	0.00 deg
Initial Orientation	Sagittal

Geometry - Saturation

Fat suppr.	None
Water suppr.	None
Restore magn.	Off
Special sat.	None

Geometry - Navigator

Geometry - Tim Planning Suite

Set-n-Go Protocol	Off
Table position	H
Table position	0 mm
Inline Composing	Off

System - Miscellaneous

Positioning mode	REF
Table position	H
Table position	0 mm
MSMA	S - C - T
Sagittal	R >> L
Coronal	A >> P
Transversal	F >> H
Coil Combine Mode	Adaptive Combine
Save uncombined	Off
Matrix Optimization	Off
AutoAlign	---
Coil Select Mode	On - AutoCoilSelect

System - Adjustments

B0 Shim mode	Tune up
B1 Shim mode	TrueForm
Adjust with body coil	Off
Confirm freq. adjustment	Off
Assume Dominant Fat	Off
Assume Silicone	Off
Adjustment Tolerance	Auto

System - Adjust Volume

Position	Isocenter
Orientation	Transversal
Rotation	0.00 deg
A >> P	263 mm
R >> L	350 mm
F >> H	350 mm
Reset	Off

System - pTx Volumes

B1 Shim mode	TrueForm

System - Tx/Rx

Frequency 1H	123.244318 MHz
Correction factor	1
Gain	High
Img. Scale Cor.	1.000
Reset	Off
? Ref. amplitude 1H	0.000 V

Physio - Signal1

1st Signal/Mode	None
TR	1500.0 ms
Concatenations	1

Physio - Cardiac

Magn. preparation	None
Fat suppr.	None
Dark blood	Off
FoV read	220 mm
FoV phase	100.0 %
Phase resolution	80 %

Physio - PACE

Resp. control	Off
Concatenations	1

Inline - Common

Subtract	Off
Measurements	1
StdDev	Off
Save original images	On

Inline - MIP

MIP-Sag	Off
MIP-Cor	Off
MIP-Tra	Off
MIP-Time	Off
Save original images	On

Inline - Composing

Inline Composing	Off
Distortion Corr.	Off

Sequence - Part 1

Introduction	On
Dimension	2D
Contrasts	1
Flow comp.	No
Multi-slice mode	Single shot
Echo spacing	7.22 ms
Bandwidth	601 Hz/Px

Sequence - Part 2

RF pulse type	Normal
Gradient mode	Whisper
Hyperecho	Off
Turbo factor	256

Sequence - Assistant

Mode	Min flip angle
Min flip angle	130 deg
Allowed delay	60 s

For peer review only

SIEMENS MAGNETOM Prisma

\\Study Protocols\BRAIN\Neonates\Theirworld - E161723\t2_blade_v3
TA: 2:29 PM: REF Voxel size: 0.7×0.7×3.0 mmPAT: 2 Rel. SNR: 1.00 : qtseBR_rr

Properties

Prio recon	Off
Load images to viewer	On
Inline movie	Off
Auto store images	On
Load images to stamp segments	On
Load images to graphic segments	Off
Auto open inline display	Off
Auto close inline display	Off
Start measurement without further preparation	Off
Wait for user to start	Off
Start measurements	Single measurement

Routine

Slice group	1
Slices	40
Dist. factor	0 %
Position	R1.2 P40.0 H50.2 mm
Orientation	Transversal
Phase enc. dir.	A >> P
AutoAlign	---
Phase oversampling	0.0 %
FoV read	220 mm
FoV phase	100.0 %
Slice thickness	3.0 mm
TR	4100.0 ms
TE	207 ms
Averages	1
Concatenations	4
Filter	Prescan Normalize
Coil elements	PeH;PeN

Contrast - Common

TR	4100.0 ms
TE	207 ms
TD	0.0 ms
MTC	Off
Magn. preparation	None
Flip angle	90 deg
Fat suppr.	None
Water suppr.	None
Restore magn.	On

Contrast - Dynamic

Averages	1
Averaging mode	Short term
Reconstruction	Magnitude
Measurements	1
Multiple series	Each measurement

Resolution - Common

FoV read	220 mm
FoV phase	100.0 %
Slice thickness	3.0 mm
Base resolution	320
BLADE coverage	100.0 %
Trajectory	BLADE
Interpolation	Off

Resolution - iPAT

PAT mode	GRAPPA
Accel. factor PE	2
Ref. lines PE	8
Reference scan mode	Integrated

Resolution - Filter Image

Image Filter	Off
Distortion Corr.	Off
Prescan Normalize	On
Unfiltered images	Off
Normalize	Off
B1 filter	Off

Resolution - Filter Rawdata

Raw filter	Off
Elliptical filter	Off

Geometry - Common

Slice group	1
Slices	40
Dist. factor	0 %
Position	R1.2 P40.0 H50.2 mm
Orientation	Transversal
Phase enc. dir.	A >> P
FoV read	220 mm
FoV phase	100.0 %
Slice thickness	3.0 mm
TR	4100.0 ms
Multi-slice mode	Interleaved
Series	Interleaved
Concatenations	4

Geometry - AutoAlign

Slice group	1
Position	R1.2 P40.0 H50.2 mm
Orientation	Transversal
Phase enc. dir.	A >> P
AutoAlign	---
Initial Position	R1.2 P40.0 H50.2
R	1.2 mm
P	40.0 mm
H	50.2 mm
Initial Rotation	0.00 deg
Initial Orientation	Transversal

Geometry - Saturation

Fat suppr.	None
Water suppr.	None
Restore magn.	On
Special sat.	None

Geometry - Navigator

Geometry - Tim Planning Suite

Set-n-Go Protocol	Off
Table position	H
Table position	0 mm
Inline Composing	Off

For peer review only - http://bmjopen.bmj.com/site/about/guidelines.xhtml

SIEMENS MAGNETOM Prisma

System - Miscellaneous

Positioning mode	REF
Table position	H
Table position	0 mm
MSMA	S - C - T
Sagittal	R >> L
Coronal	A >> P
Transversal	F >> H
Coil Combine Mode	Adaptive Combine
Save uncombined	Off
Matrix Optimization	Off
AutoAlign	---
Coil Select Mode	On - AutoCoilSelect

System - Adjustments

B0 Shim mode	Tune up
B1 Shim mode	TrueForm
Adjust with body coil	Off
Confirm freq. adjustment	Off
Assume Dominant Fat	Off
Assume Silicone	Off
Adjustment Tolerance	Auto

System - Adjust Volume

Position	Isocenter
Orientation	Transversal
Rotation	0.00 deg
A >> P	263 mm
R >> L	350 mm
F >> H	350 mm
Reset	Off

System - pTx Volumes

B1 Shim mode	TrueForm

System - Tx/Rx

Frequency 1H	123.244318 MHz
Correction factor	1
Gain	High
Img. Scale Cor.	1.000
Reset	Off
? Ref. amplitude 1H	0.000 V

Physio - Signal1

1st Signal/Mode	None
TR	4100.0 ms
Concatenations	4

Physio - Cardiac

Magn. preparation	None
Fat suppr.	None
Dark blood	Off
FoV read	220 mm
FoV phase	100.0 %
BLADE coverage	100.0 %
Trajectory	BLADE

Physio - PACE

Resp. control	Off
Concatenations	4

Inline - Common

Subtract	Off
Measurements	1

Inline - Common

StdDev	Off
Save original images	On

Inline - MIP

MIP-Sag	Off
MIP-Cor	Off
MIP-Tra	Off
MIP-Time	Off
Save original images	On

Inline - Composing

Inline Composing	Off
Distortion Corr.	Off

Sequence - Part 1

Introduction	On
Dimension	2D
Compensate T2 decay	Off
Contrasts	1
Flow comp.	Read
Multi-slice mode	Interleaved
Free echo spacing	Off
Echo spacing	10.9 ms
Bandwidth	363 Hz/Px

Sequence - Part 2

Define	Turbo factor
Echo trains per slice	8
Phase correction	Automatic
Acoustic noise reduction	Active
RF pulse type	Low SAR
Gradient mode	Fast
Hyperecho	On
WARP	Off
Motion correction	On
Red. EC sensitivity	Off
Turbo factor	36

Sequence - Assistant

Mode	Off
Allowed delay	30 s

For peer review only

For peer review only - http://bmjopen.bmj.com/site/about/guidelines.xhtml

SIEMENS MAGNETOM Prisma

\\Study Protocols\BRAIN\Neonates\Theirworld - E161723\t2_space_sag_p4_iso_v2x
TA: 2:13 PM: REF Voxel size: 1.0×1.0×1.0 mmPAT: 4 Rel. SNR: 1.00 : spcR

Properties

Prio recon	Off
Load images to viewer	On
Inline movie	Off
Auto store images	On
Load images to stamp segments	Off
Load images to graphic segments	Off
Auto open inline display	Off
Auto close inline display	Off
Start measurement without further preparation	Off
Wait for user to start	Off
Start measurements	Single measurement

Routine

Slab group	1
Slabs	1
Position	R1.2 P36.9 H0.0 mm
Orientation	Sagittal
Phase enc. dir.	A >> P
AutoAlign	---
Phase oversampling	0 %
Slice oversampling	0.0 %
Slices per slab	160
FoV read	128 mm
FoV phase	150.0 %
Slice thickness	1.00 mm
TR	3200 ms
TE	409 ms
Averages	1.4
Concatenations	1
Filter	Raw filter, Prescan Normalize
Coil elements	PeH;PeN

Contrast - Common

TR	3200 ms
TE	409 ms
MTC	Off
Magn. preparation	None
Fat suppr.	Fat sat.
Fat sat. mode	Strong
Blood suppr.	Off
Restore magn.	On

Contrast - Dynamic

Averages	1.4
Reconstruction	Magnitude
Measurements	1
Multiple series	Each measurement

Resolution - Common

FoV read	128 mm
FoV phase	150.0 %
Slice thickness	1.00 mm
Base resolution	128
Phase resolution	100 %
Slice resolution	100 %
Phase partial Fourier	Allowed
Slice partial Fourier	Off
Interpolation	Off

Resolution - iPAT

PAT mode	GRAPPA
Accel. factor PE	2
Ref. lines PE	24
Accel. factor 3D	2
Ref. lines 3D	24
Reference scan mode	Integrated

Resolution - Filter Image

Image Filter	Off
Distortion Corr.	Off
Prescan Normalize	On
Unfiltered images	Off
Normalize	Off
B1 filter	Off

Resolution - Filter Rawdata

Raw filter	On
Elliptical filter	Off

Geometry - Common

Slab group	1
Slabs	1
Position	R1.2 P36.9 H0.0 mm
Orientation	Sagittal
Phase enc. dir.	A >> P
Slice oversampling	0.0 %
Slices per slab	160
FoV read	128 mm
FoV phase	150.0 %
Slice thickness	1.00 mm
TR	3200 ms
Series	Interleaved
Concatenations	1

Geometry - AutoAlign

Slab group	1
Position	R1.2 P36.9 H0.0 mm
Orientation	Sagittal
Phase enc. dir.	A >> P
AutoAlign	---
Initial Position	R1.2 P36.9 H0.0
R	1.2 mm
P	36.9 mm
H	0.0 mm
Initial Rotation	0.00 deg
Initial Orientation	Sagittal

Geometry - Saturation

Fat suppr.	Fat sat.
Fat sat. mode	Strong
Restore magn.	On
Special sat.	None

Geometry - Navigator

Geometry - Tim Planning Suite

Set-n-Go Protocol	Off
Table position	H
Table position	0 mm
Inline Composing	Off

For peer review only - http://bmjopen.bmj.com/site/about/guidelines.xhtml

SIEMENS MAGNETOM Prisma

System - Miscellaneous

Positioning mode	REF
Table position	H
Table position	0 mm
MSMA	S - C - T
Sagittal	R >> L
Coronal	A >> P
Transversal	F >> H
Coil Combine Mode	Adaptive Combine
Save uncombined	Off
Matrix Optimization	Off
AutoAlign	---
Coil Select Mode	On - AutoCoilSelect

System - Adjustments

B0 Shim mode	Standard
B1 Shim mode	TrueForm
Adjust with body coil	Off
Confirm freq. adjustment	Off
Assume Dominant Fat	Off
Assume Silicone	Off
Adjustment Tolerance	Auto

System - Adjust Volume

Position	R1.2 P36.9 H0.0 mm
Orientation	Sagittal
Rotation	90.00 deg
F >> H	128 mm
A >> P	192 mm
R >> L	160 mm
Reset	Off

System - pTx Volumes

B1 Shim mode	TrueForm
Excitation	Non-sel.

System - Tx/Rx

Frequency 1H	123.244318 MHz
Correction factor	1
Gain	High
Img. Scale Cor.	3.000
Reset	Off
? Ref. amplitude 1H	0.000 V

Physio - Signal1

1st Signal/Mode	None
Trigger delay	0 ms
TR	3200 ms
Concatenations	1

Physio - Cardiac

Magn. preparation	None
Fat suppr.	Fat sat.
Dark blood	Off
FoV read	128 mm
FoV phase	150.0 %
Phase resolution	100 %

Physio - PACE

Resp. control	Off
Concatenations	1

Inline - Common

Subtract	Off

Inline - Common

Measurements	1
StdDev	Off
Save original images	On

Inline - MIP

MIP-Sag	Off
MIP-Cor	Off
MIP-Tra	Off
MIP-Time	Off
Save original images	On

Inline - Composing

Inline Composing	Off
Distortion Corr.	Off

Sequence - Part 1

Introduction	On
Dimension	3D
Elliptical scanning	Off
Reordering	Linear
Flow comp.	No
Echo spacing	4.4 ms
Adiabatic-mode	Off
Bandwidth	592 Hz/Px

Sequence - Part 2

Echo train duration	1034 ms
RF pulse type	Low SAR
Gradient mode	Whisper
Excitation	Non-sel.
Flip angle mode	T2 var
Turbo factor	282

Sequence - Assistant

Allowed delay	30 s

For peer review only

SIEMENS MAGNETOM Prisma

\\Study Protocols\BRAIN\Neonates\Theirworld - E161723\DTI_Neonate_v6b_dummy
TA: 0:28 PM: FIX Voxel size: 2.0×2.0×2.0 mmPAT: 4 Rel. SNR: 1.00 : epse

Properties

Prio recon	Off
Load images to viewer	On
Inline movie	Off
Auto store images	On
Load images to stamp segments	Off
Load images to graphic segments	Off
Auto open inline display	Off
Auto close inline display	Off
Start measurement without further preparation	Off
Wait for user to start	Off
Start measurements	Single measurement

Routine

Slice group	1
Slices	58
Dist. factor	0 %
Position	R1.2 P39.7 H47.8 mm
Orientation	Transversal
Phase enc. dir.	R >> L
AutoAlign	---
Phase oversampling	0 %
FoV read	256 mm
FoV phase	100.0 %
Slice thickness	2.0 mm
TR	3500 ms
TE	78.0 ms
Concatenations	1
Filter	Raw filter, Prescan Normalize
Coil elements	PeH;PeN

Contrast - Common

TR	3500 ms
TE	78.0 ms
MTC	Off
Magn. preparation	None
Fat suppr.	Fat sat.
Fat sat. mode	Strong

Contrast - Dynamic

Averaging mode	Long term
Reconstruction	Magnitude
Measurements	1
Delay in TR	0 ms
Multiple series	Off

Resolution - Common

FoV read	256 mm
FoV phase	100.0 %
Slice thickness	2.0 mm
Base resolution	128
Phase resolution	100 %
Phase partial Fourier	7/8
Interpolation	Off

Resolution - iPAT

Accel. mode	Slice accel.
Accel. factor PE	2
Ref. lines PE	40

Resolution - iPAT

Accel. factor slice	2
Reference scan mode	EPI/separate

Resolution - Filter Image

Distortion Corr.	Off
Prescan Normalize	On
Dynamic Field Corr.	Off

Resolution - Filter Rawdata

Raw filter	On
Elliptical filter	Off

Geometry - Common

Slice group	1
Slices	58
Dist. factor	0 %
Position	R1.2 P39.7 H47.8 mm
Orientation	Transversal
Phase enc. dir.	R >> L
FoV read	256 mm
FoV phase	100.0 %
Slice thickness	2.0 mm
TR	3500 ms
Multi-slice mode	Interleaved
Series	Interleaved
Concatenations	1

Geometry - AutoAlign

Slice group	1
Position	R1.2 P39.7 H47.8 mm
Orientation	Transversal
Phase enc. dir.	R >> L
AutoAlign	---
Initial Position	R1.2 P39.7 H47.8
R	1.2 mm
P	39.7 mm
H	47.8 mm
Initial Rotation	90.00 deg
Initial Orientation	Transversal

Geometry - Saturation

Fat suppr.	Fat sat.
Fat sat. mode	Strong
Special sat.	None

Geometry - Navigator

Geometry - Tim Planning Suite

Set-n-Go Protocol	Off
Table position	H
Table position	0 mm
Inline Composing	Off

System - Miscellaneous

Positioning mode	FIX
Table position	H
Table position	0 mm
MSMA	S - C - T
Sagittal	R >> L

For peer review only - http://bmjopen.bmj.com/site/about/guidelines.xhtml

SIEMENS MAGNETOM Prisma

System - Miscellaneous

Coronal	A >> P
Transversal	F >> H
Coil Combine Mode	Adaptive Combine
Matrix Optimization	Performance
AutoAlign	---
Coil Select Mode	On - AutoCoilSelect

System - Adjustments

B0 Shim mode	Standard
B1 Shim mode	TrueForm
Adjust with body coil	Off
Confirm freq. adjustment	Off
Assume Dominant Fat	Off
Assume Silicone	Off
Adjustment Tolerance	Auto

System - Adjust Volume

Position	R1.2 P39.7 H47.8 mm
Orientation	Transversal
Rotation	90.00 deg
R >> L	256 mm
A >> P	256 mm
F >> H	116 mm
Reset	Off

System - pTx Volumes

B1 Shim mode	TrueForm
Excitation	Standard

System - Tx/Rx

Frequency 1H	123.244318 MHz
Correction factor	1
Gain	High
Img. Scale Cor.	1.000
Reset	Off
? Ref. amplitude 1H	0.000 V

Physio - Signal1

1st Signal/Mode	None
TR	3500 ms
Concatenations	1

Physio - PACE

Resp. control	Off
Concatenations	1

Diff - Neuro

Diffusion mode	Free
Diff. directions	71
Diffusion Scheme	Monopolar
Diff. weightings	1
b-value	0 s/mm²
b-value	3
Diff. weighted images	On
Trace weighted images	Off
ADC maps	Off
FA maps	Off
Mosaic	Off
Tensor	Off
Noise level	40

Diff - Body

Diffusion mode	Free
Diff. directions	71
Diffusion Scheme	Monopolar
Diff. weightings	1
b-value	0 s/mm²
b-value	3
Diff. weighted images	On
Trace weighted images	Off
ADC maps	Off
Exponential ADC Maps	Off
FA maps	Off
Invert Gray Scale	Off
Calculated Image	Off
b-Value >=	0 s/mm²
Noise level	40

Diff - Composing

Inline Composing	Off
Distortion Corr.	Off

Sequence - Part 1

Introduction	Off
Optimization	None
Multi-slice mode	Interleaved
Free echo spacing	Off
Echo spacing	0.78 ms
Bandwidth	1446 Hz/Px

Sequence - Part 2

EPI factor	128
RF pulse type	Low SAR
Gradient mode	Normal
Excitation	Standard

Sequence - pTX Pulses

For peer review only

For peer review only - http://bmjopen.bmj.com/site/about/guidelines.xhtml

SIEMENS MAGNETOM Prisma

\\Study Protocols\BRAIN\Neonates\Theirworld - E161723\DTI_Neonate_v6b_rev

TA: 0:28 PM: FIX Voxel size: 2.0×2.0×2.0 mmPAT: 4 Rel. SNR: 1.00 : epse

Properties

Prio recon	Off
Load images to viewer	On
Inline movie	Off
Auto store images	On
Load images to stamp segments	Off
Load images to graphic segments	Off
Auto open inline display	Off
Auto close inline display	Off
Start measurement without further preparation	Off
Wait for user to start	Off
Start measurements	Single measurement

Routine

Slice group	1
Slices	58
Dist. factor	0 %
Position	R1.2 P39.7 H47.8 mm
Orientation	Transversal
Phase enc. dir.	R >> L
AutoAlign	---
Phase oversampling	0 %
FoV read	256 mm
FoV phase	100.0 %
Slice thickness	2.0 mm
TR	3500 ms
TE	78.0 ms
Concatenations	1
Filter	Raw filter, Prescan Normalize
Coil elements	PeH;PeN

Contrast - Common

TR	3500 ms
TE	78.0 ms
MTC	Off
Magn. preparation	None
Fat suppr.	Fat sat.
Fat sat. mode	Strong

Contrast - Dynamic

Averaging mode	Long term
Reconstruction	Magnitude
Measurements	1
Delay in TR	0 ms
Multiple series	Off

Resolution - Common

FoV read	256 mm
FoV phase	100.0 %
Slice thickness	2.0 mm
Base resolution	128
Phase resolution	100 %
Phase partial Fourier	7/8
Interpolation	Off

Resolution - iPAT

Accel. mode	Slice accel.
Accel. factor PE	2
Ref. lines PE	40

Resolution - iPAT

Accel. factor slice	2
Reference scan mode	EPI/separate

Resolution - Filter Image

Distortion Corr.	Off
Prescan Normalize	On
Dynamic Field Corr.	Off

Resolution - Filter Rawdata

Raw filter	On
Elliptical filter	Off

Geometry - Common

Slice group	1
Slices	58
Dist. factor	0 %
Position	R1.2 P39.7 H47.8 mm
Orientation	Transversal
Phase enc. dir.	R >> L
FoV read	256 mm
FoV phase	100.0 %
Slice thickness	2.0 mm
TR	3500 ms
Multi-slice mode	Interleaved
Series	Interleaved
Concatenations	1

Geometry - AutoAlign

Slice group	1
Position	R1.2 P39.7 H47.8 mm
Orientation	Transversal
Phase enc. dir.	R >> L
AutoAlign	---
Initial Position	R1.2 P39.7 H47.8
R	1.2 mm
P	39.7 mm
H	47.8 mm
Initial Rotation	90.00 deg
Initial Orientation	Transversal

Geometry - Saturation

Fat suppr.	Fat sat.
Fat sat. mode	Strong
Special sat.	None

Geometry - Navigator

Geometry - Tim Planning Suite

Set-n-Go Protocol	Off
Table position	H
Table position	0 mm
Inline Composing	Off

System - Miscellaneous

Positioning mode	FIX
Table position	H
Table position	0 mm
MSMA	S - C - T
Sagittal	R >> L

For peer review only

For peer review only - http://bmjopen.bmj.com/site/about/guidelines.xhtml

SIEMENS MAGNETOM Prisma

System - Miscellaneous

Coronal	A >> P
Transversal	F >> H
Coil Combine Mode	Adaptive Combine
Matrix Optimization	Performance
AutoAlign	---
Coil Select Mode	On - AutoCoilSelect

System - Adjustments

B0 Shim mode	Standard
B1 Shim mode	TrueForm
Adjust with body coil	Off
Confirm freq. adjustment	Off
Assume Dominant Fat	Off
Assume Silicone	Off
Adjustment Tolerance	Auto

System - Adjust Volume

Position	R1.2 P39.7 H47.8 mm
Orientation	Transversal
Rotation	90.00 deg
R >> L	256 mm
A >> P	256 mm
F >> H	116 mm
Reset	Off

System - pTx Volumes

B1 Shim mode	TrueForm
Excitation	Standard

System - Tx/Rx

Frequency 1H	123.244318 MHz
Correction factor	1
Gain	High
Img. Scale Cor.	1.000
Reset	Off
? Ref. amplitude 1H	0.000 V

Physio - Signal1

1st Signal/Mode	None
TR	3500 ms
Concatenations	1

Physio - PACE

Resp. control	Off
Concatenations	1

Diff - Neuro

Diffusion mode	MDDW
Diff. directions	6
Diffusion Scheme	Monopolar
Diff. weightings	1
b-value	0 s/mm²
b-value	3
Diff. weighted images	On
Trace weighted images	Off
ADC maps	Off
FA maps	Off
Mosaic	Off
Tensor	Off
Noise level	40

Diff - Body

Diffusion mode	MDDW
Diff. directions	6
Diffusion Scheme	Monopolar
Diff. weightings	1
b-value	0 s/mm²
b-value	3
Diff. weighted images	On
Trace weighted images	Off
ADC maps	Off
Exponential ADC Maps	Off
FA maps	Off
Invert Gray Scale	Off
Calculated Image	Off
b-Value >=	0 s/mm²
Noise level	40

Diff - Composing

Inline Composing	Off
Distortion Corr.	Off

Sequence - Part 1

Introduction	Off
Optimization	None
Multi-slice mode	Interleaved
Free echo spacing	Off
Echo spacing	0.78 ms
Bandwidth	1446 Hz/Px

Sequence - Part 2

EPI factor	128
RF pulse type	Low SAR
Gradient mode	Normal
Excitation	Standard

Sequence - pTX Pulses

For peer review only

SIEMENS MAGNETOM Prisma

\\Study Protocols\BRAIN\Neonates\Theirworld - E161723\DTI_Neonate_v6b_pt1

TA: 4:29 PM: FIX Voxel size: 2.0×2.0×2.0 mmPAT: 4 Rel. SNR: 1.00 : epse

Properties

Prio recon	Off
Load images to viewer	On
Inline movie	Off
Auto store images	On
Load images to stamp segments	Off
Load images to graphic segments	Off
Auto open inline display	Off
Auto close inline display	Off
Start measurement without further preparation	Off
Wait for user to start	Off
Start measurements	Single measurement

Routine

Slice group	1
Slices	58
Dist. factor	0 %
Position	R1.2 P39.7 H47.8 mm
Orientation	Transversal
Phase enc. dir.	L >> R
AutoAlign	---
Phase oversampling	0 %
FoV read	256 mm
FoV phase	100.0 %
Slice thickness	2.0 mm
TR	3500 ms
TE	78.0 ms
Averages	1
Concatenations	1
Filter	Raw filter, Prescan Normalize
Coil elements	PeH;PeN

Contrast - Common

TR	3500 ms
TE	78.0 ms
MTC	Off
Magn. preparation	None
Fat suppr.	Fat sat.
Fat sat. mode	Strong

Contrast - Dynamic

Averages	1
Averaging mode	Long term
Reconstruction	Magnitude
Measurements	1
Delay in TR	0 ms
Multiple series	Off

Resolution - Common

FoV read	256 mm
FoV phase	100.0 %
Slice thickness	2.0 mm
Base resolution	128
Phase resolution	100 %
Phase partial Fourier	7/8
Interpolation	Off

Resolution - iPAT

Accel. mode	Slice accel.

Resolution - iPAT

Accel. factor PE	2
Ref. lines PE	40
Accel. factor slice	2
Reference scan mode	EPI/separate

Resolution - Filter Image

Distortion Corr.	Off
Prescan Normalize	On
Dynamic Field Corr.	Off

Resolution - Filter Rawdata

Raw filter	On
Elliptical filter	Off

Geometry - Common

Slice group	1
Slices	58
Dist. factor	0 %
Position	R1.2 P39.7 H47.8 mm
Orientation	Transversal
Phase enc. dir.	L >> R
FoV read	256 mm
FoV phase	100.0 %
Slice thickness	2.0 mm
TR	3500 ms
Multi-slice mode	Interleaved
Series	Interleaved
Concatenations	1

Geometry - AutoAlign

Slice group	1
Position	R1.2 P39.7 H47.8 mm
Orientation	Transversal
Phase enc. dir.	L >> R
AutoAlign	---
Initial Position	R1.2 P39.7 H47.8
R	1.2 mm
P	39.7 mm
H	47.8 mm
Initial Rotation	-90.00 deg
Initial Orientation	Transversal

Geometry - Saturation

Fat suppr.	Fat sat.
Fat sat. mode	Strong
Special sat.	None

Geometry - Navigator

Geometry - Tim Planning Suite

Set-n-Go Protocol	Off
Table position	H
Table position	0 mm
Inline Composing	Off

System - Miscellaneous

Positioning mode	FIX
Table position	H
Table position	0 mm

For peer review only - http://bmjopen.bmj.com/site/about/guidelines.xhtml

SIEMENS MAGNETOM Prisma

System - Miscellaneous

MSMA	S - C - T
Sagittal	R >> L
Coronal	A >> P
Transversal	F >> H
Coil Combine Mode	Adaptive Combine
Matrix Optimization	Performance
AutoAlign	---
Coil Select Mode	On - AutoCoilSelect

System - Adjustments

B0 Shim mode	Standard
B1 Shim mode	TrueForm
Adjust with body coil	Off
Confirm freq. adjustment	Off
Assume Dominant Fat	Off
Assume Silicone	Off
Adjustment Tolerance	Auto

System - Adjust Volume

Position	R1.2 P39.7 H47.8 mm
Orientation	Transversal
Rotation	-90.00 deg
R >> L	256 mm
A >> P	256 mm
F >> H	116 mm
Reset	Off

System - pTx Volumes

B1 Shim mode	TrueForm
Excitation	Standard

System - Tx/Rx

Frequency 1H	123.244318 MHz
Correction factor	1
Gain	High
Img. Scale Cor.	1.000
Reset	Off
? Ref. amplitude 1H	0.000 V

Physio - Signal1

1st Signal/Mode	None
TR	3500 ms
Concatenations	1

Physio - PACE

Resp. control	Off
Concatenations	1

Diff - Neuro

Diffusion mode	Free
Diff. directions	71
Diffusion Scheme	Monopolar
Diff. weightings	2
b-value 1	0 s/mm^2
b-value 2	750 s/mm^2
b-value 1	1
b-value 2	1
Diff. weighted images	On
Trace weighted images	Off
ADC maps	Off
FA maps	Off
Mosaic	On
Tensor	Off

Diff - Neuro

Noise level	40

Diff - Body

Diffusion mode	Free
Diff. directions	71
Diffusion Scheme	Monopolar
Diff. weightings	2
b-value 1	0 s/mm^2
b-value 2	750 s/mm^2
b-value 1	1
b-value 2	1
Diff. weighted images	On
Trace weighted images	Off
ADC maps	Off
Exponential ADC Maps	Off
FA maps	Off
Invert Gray Scale	Off
Calculated Image	Off
b-Value >=	0 s/mm^2
Noise level	40

Diff - Composing

Inline Composing	Off
Distortion Corr.	Off

Sequence - Part 1

Introduction	Off
Optimization	None
Multi-slice mode	Interleaved
Free echo spacing	Off
Echo spacing	0.78 ms
Bandwidth	1446 Hz/Px

Sequence - Part 2

EPI factor	128
RF pulse type	Low SAR
Gradient mode	Normal
Excitation	Standard

Sequence - pTX Pulses

For peer review only

For peer review only - http://bmjopen.bmj.com/site/about/guidelines.xhtml

SIEMENS MAGNETOM Prisma

\\Study Protocols\BRAIN\Neonates\Theirworld - E161723\DTI_Neonate_v6b_pt2

TA: 5:01 PM: FIX Voxel size: 2.0×2.0×2.0 mmPAT: 4 Rel. SNR: 1.00 : epse

Properties

Prio recon	Off
Load images to viewer	On
Inline movie	Off
Auto store images	On
Load images to stamp segments	Off
Load images to graphic segments	Off
Auto open inline display	Off
Auto close inline display	Off
Start measurement without further preparation	Off
Wait for user to start	Off
Start measurements	Single measurement

Routine

Slice group	1
Slices	58
Dist. factor	0 %
Position	R1.2 P39.7 H47.8 mm
Orientation	Transversal
Phase enc. dir.	L >> R
AutoAlign	---
Phase oversampling	0 %
FoV read	256 mm
FoV phase	100.0 %
Slice thickness	2.0 mm
TR	3500 ms
TE	78.0 ms
Averages	1
Concatenations	1
Filter	Raw filter, Prescan Normalize
Coil elements	PeH;PeN

Contrast - Common

TR	3500 ms
TE	78.0 ms
MTC	Off
Magn. preparation	None
Fat suppr.	Fat sat.
Fat sat. mode	Strong

Contrast - Dynamic

Averages	1
Averaging mode	Long term
Reconstruction	Magnitude
Measurements	1
Delay in TR	0 ms
Multiple series	Off

Resolution - Common

FoV read	256 mm
FoV phase	100.0 %
Slice thickness	2.0 mm
Base resolution	128
Phase resolution	100 %
Phase partial Fourier	7/8
Interpolation	Off

Resolution - iPAT

Accel. mode	Slice accel.

Resolution - iPAT

Accel. factor PE	2
Ref. lines PE	40
Accel. factor slice	2
Reference scan mode	EPI/separate

Resolution - Filter Image

Distortion Corr.	Off
Prescan Normalize	On
Dynamic Field Corr.	Off

Resolution - Filter Rawdata

Raw filter	On
Elliptical filter	Off

Geometry - Common

Slice group	1
Slices	58
Dist. factor	0 %
Position	R1.2 P39.7 H47.8 mm
Orientation	Transversal
Phase enc. dir.	L >> R
FoV read	256 mm
FoV phase	100.0 %
Slice thickness	2.0 mm
TR	3500 ms
Multi-slice mode	Interleaved
Series	Interleaved
Concatenations	1

Geometry - AutoAlign

Slice group	1
Position	R1.2 P39.7 H47.8 mm
Orientation	Transversal
Phase enc. dir.	L >> R
AutoAlign	---
Initial Position	R1.2 P39.7 H47.8
R	1.2 mm
P	39.7 mm
H	47.8 mm
Initial Rotation	-90.00 deg
Initial Orientation	Transversal

Geometry - Saturation

Fat suppr.	Fat sat.
Fat sat. mode	Strong
Special sat.	None

Geometry - Navigator

Geometry - Tim Planning Suite

Set-n-Go Protocol	Off
Table position	H
Table position	0 mm
Inline Composing	Off

System - Miscellaneous

Positioning mode	FIX
Table position	H
Table position	0 mm

For peer review only - http://bmjopen.bmj.com/site/about/guidelines.xhtml

SIEMENS MAGNETOM Prisma

System - Miscellaneous

MSMA	S - C - T
Sagittal	R >> L
Coronal	A >> P
Transversal	F >> H
Coil Combine Mode	Adaptive Combine
Matrix Optimization	Performance
AutoAlign	---
Coil Select Mode	On - AutoCoilSelect

System - Adjustments

B0 Shim mode	Standard
B1 Shim mode	TrueForm
Adjust with body coil	Off
Confirm freq. adjustment	Off
Assume Dominant Fat	Off
Assume Silicone	Off
Adjustment Tolerance	Auto

System - Adjust Volume

Position	R1.2 P39.7 H47.8 mm
Orientation	Transversal
Rotation	-90.00 deg
R >> L	256 mm
A >> P	256 mm
F >> H	116 mm
Reset	Off

System - pTx Volumes

B1 Shim mode	TrueForm
Excitation	Standard

System - Tx/Rx

Frequency 1H	123.244318 MHz
Correction factor	1
Gain	High
Img. Scale Cor.	1.000
Reset	Off
? Ref. amplitude 1H	0.000 V

Physio - Signal1

1st Signal/Mode	None
TR	3500 ms
Concatenations	1

Physio - PACE

Resp. control	Off
Concatenations	1

Diff - Neuro

Diffusion mode	Free
Diff. directions	80
Diffusion Scheme	Monopolar
Diff. weightings	2
b-value 1	0 s/mm²
b-value 2	2500 s/mm²
b-value 1	1
b-value 2	1
Diff. weighted images	On
Trace weighted images	Off
ADC maps	Off
FA maps	Off
Mosaic	On
Tensor	Off

Diff - Neuro

Noise level	40

Diff - Body

Diffusion mode	Free
Diff. directions	80
Diffusion Scheme	Monopolar
Diff. weightings	2
b-value 1	0 s/mm²
b-value 2	2500 s/mm²
b-value 1	1
b-value 2	1
Diff. weighted images	On
Trace weighted images	Off
ADC maps	Off
Exponential ADC Maps	Off
FA maps	Off
Invert Gray Scale	Off
Calculated Image	Off
b-Value >=	0 s/mm²
Noise level	40

Diff - Composing

Inline Composing	Off
Distortion Corr.	Off

Sequence - Part 1

Introduction	Off
Optimization	None
Multi-slice mode	Interleaved
Free echo spacing	Off
Echo spacing	0.78 ms
Bandwidth	1446 Hz/Px

Sequence - Part 2

EPI factor	128
RF pulse type	Low SAR
Gradient mode	Normal
Excitation	Standard

Sequence - pTX Pulses

For peer review only

For peer review only - http://bmjopen.bmj.com/site/about/guidelines.xhtml

SIEMENS MAGNETOM Prisma

\\Study Protocols\BRAIN\Neonates\Theirworld - E161723\MPRAGE-v4

TA: 3:09 PM: FIX Voxel size: 1.0×1.0×1.0 mmPAT: 2 Rel. SNR: 1.00 : tfl

Properties

Prio recon	Off
Load images to viewer	On
Inline movie	Off
Auto store images	On
Load images to stamp segments	On
Load images to graphic segments	Off
Auto open inline display	Off
Auto close inline display	Off
Start measurement without further preparation	Off
Wait for user to start	Off
Start measurements	Single measurement

Routine

Slab group	1
Slabs	1
Dist. factor	50 %
Position	R1.1 P38.9 F20.7 mm
Orientation	Sagittal
Phase enc. dir.	A >> P
AutoAlign	---
Phase oversampling	20 %
Slice oversampling	0.0 %
Slices per slab	160
FoV read	160 mm
FoV phase	100.0 %
Slice thickness	1.00 mm
TR	1970.0 ms
TE	4.69 ms
Averages	1
Concatenations	1
Filter	Prescan Normalize
Coil elements	PeH;PeN;SP1

Contrast - Common

TR	1970.0 ms
TE	4.69 ms
Magn. preparation	Non-sel. IR
TI	1100 ms
Flip angle	9 deg
Fat suppr.	None
Water suppr.	None

Contrast - Dynamic

Averages	1
Averaging mode	Long term
Reconstruction	Magnitude
Measurements	1
Multiple series	Each measurement

Resolution - Common

FoV read	160 mm
FoV phase	100.0 %
Slice thickness	1.00 mm
Base resolution	160
Phase resolution	100 %
Slice resolution	100 %
Phase partial Fourier	7/8
Slice partial Fourier	Off
Interpolation	Off

Resolution - iPAT

PAT mode	GRAPPA
Accel. factor PE	2
Ref. lines PE	24
Accel. factor 3D	1
Reference scan mode	Integrated

Resolution - Filter Image

Image Filter	Off
Distortion Corr.	Off
Prescan Normalize	On
Unfiltered images	Off
Normalize	Off
B1 filter	Off

Resolution - Filter Rawdata

Raw filter	Off
Elliptical filter	Off

Geometry - Common

Slab group	1
Slabs	1
Dist. factor	50 %
Position	R1.1 P38.9 F20.7 mm
Orientation	Sagittal
Phase enc. dir.	A >> P
Slice oversampling	0.0 %
Slices per slab	160
FoV read	160 mm
FoV phase	100.0 %
Slice thickness	1.00 mm
TR	1970.0 ms
Multi-slice mode	Single shot
Series	Interleaved
Concatenations	1

Geometry - AutoAlign

Slab group	1
Position	R1.1 P38.9 F20.7 mm
Orientation	Sagittal
Phase enc. dir.	A >> P
AutoAlign	---
Initial Position	R1.1 P38.9 F20.7
R	1.1 mm
P	38.9 mm
F	20.7 mm
Initial Rotation	0.00 deg
Initial Orientation	Sagittal

Geometry - Navigator

Geometry - Tim Planning Suite

Set-n-Go Protocol	Off
Table position	H
Table position	0 mm
Inline Composing	Off

System - Miscellaneous

Positioning mode	FIX
Table position	H
Table position	0 mm

For peer review only

For peer review only - http://bmjopen.bmj.com/site/about/guidelines.xhtml

SIEMENS MAGNETOM Prisma

System - Miscellaneous

MSMA	S - C - T
Sagittal	R >> L
Coronal	A >> P
Transversal	F >> H
Coil Combine Mode	Adaptive Combine
Save uncombined	Off
Matrix Optimization	Off
Coil Focus	Flat
AutoAlign	---
Coil Select Mode	On - AutoCoilSelect

System - Adjustments

B0 Shim mode	Standard
B1 Shim mode	TrueForm
Adjust with body coil	Off
Confirm freq. adjustment	Off
Assume Dominant Fat	Off
Assume Silicone	Off
Adjustment Tolerance	Auto

System - Adjust Volume

Position	R1.1 P38.9 F20.7 mm
Orientation	Sagittal
Rotation	0.00 deg
A >> P	160 mm
F >> H	160 mm
R >> L	160 mm
Reset	Off

System - pTx Volumes

B1 Shim mode	TrueForm
Excitation	Non-sel.

System - Tx/Rx

Frequency 1H	123.244318 MHz
Correction factor	1
Gain	Low
Img. Scale Cor.	4.000
Reset	Off
? Ref. amplitude 1H	0.000 V

Physio - Signal1

1st Signal/Mode	None
TR	1970.0 ms
Concatenations	1

Physio - Cardiac

Magn. preparation	Non-sel. IR
TI	1100 ms
Fat suppr.	None
Dark blood	Off
FoV read	160 mm
FoV phase	100.0 %
Phase resolution	100 %

Physio - PACE

Resp. control	Off
Concatenations	1

Inline - Common

Subtract	Off
Measurements	1
StdDev	Off

Inline - Common

Save original images	On

Inline - MIP

MIP-Sag	Off
MIP-Cor	Off
MIP-Tra	Off
MIP-Time	Off
Save original images	On

Inline - Composing

Inline Composing	Off
Distortion Corr.	Off

Inline - MapIt

Save original images	On
MapIt	None
Flip angle	9 deg
Measurements	1
TR	1970.0 ms
TE	4.69 ms

Sequence - Part 1

Introduction	On
Dimension	3D
Elliptical scanning	Off
Reordering	Linear
Asymmetric echo	Off
Flow comp.	No
Multi-slice mode	Single shot
Echo spacing	10.8 ms
Bandwidth	140 Hz/Px

Sequence - Part 2

RF pulse type	Normal
Gradient mode	Whisper
Excitation	Non-sel.
RF spoiling	On
Incr. Gradient spoiling	Off
Turbo factor	160

Sequence - Assistant

Mode	Off

For peer review only

SIEMENS MAGNETOM Prisma

\\Study Protocols\BRAIN\Neonates\Theirworld - E161723\SWI_v2

TA: 2:23 PM: FIX Voxel size: 0.8×0.8×3.0 mmPAT: 3 Rel. SNR: 1.00 : qswi_r

Properties

Prio recon	Off
Load images to viewer	On
Inline movie	Off
Auto store images	On
Load images to stamp segments	Off
Load images to graphic segments	Off
Auto open inline display	Off
Auto close inline display	Off
Start measurement without further preparation	Off
Wait for user to start	Off
Start measurements	Single measurement

Routine

Slab group	1
Slabs	1
Dist. factor	20 %
Position	L0.0 A2.3 H2.2 mm
Orientation	Transversal
Phase enc. dir.	R >> L
AutoAlign	---
Phase oversampling	0 %
Slice oversampling	20.0 %
Slices per slab	40
FoV read	240 mm
FoV phase	84.4 %
Slice thickness	3.00 mm
TR	28.0 ms
TE	20.00 ms
Averages	1
Concatenations	1
Filter	Prescan Normalize
Coil elements	HEA;HEP

Contrast - Common

TR	28.0 ms
TE	20.00 ms
MTC	Off
Magn. preparation	None
Flip angle	9 deg
Fat suppr.	None
Water suppr.	None
SWI	On

Contrast - Dynamic

Averages	1
Averaging mode	Short term
Reconstruction	Magn./Phase
Measurements	1
Multiple series	Each measurement

Resolution - Common

FoV read	240 mm
FoV phase	84.4 %
Slice thickness	3.00 mm
Base resolution	320
Phase resolution	100 %
Slice resolution	100 %
Phase partial Fourier	Off
Slice partial Fourier	Off

Resolution - Common

Interpolation	Off

Resolution - iPAT

PAT mode	GRAPPA
Accel. factor PE	3
Ref. lines PE	24
Accel. factor 3D	1
Reference scan mode	Integrated

Resolution - Filter Image

Image Filter	Off
Distortion Corr.	Off
Prescan Normalize	On
Unfiltered images	Off
Normalize	Off
B1 filter	Off

Resolution - Filter Rawdata

Raw filter	Off
Elliptical filter	Off

Geometry - Common

Slab group	1
Slabs	1
Dist. factor	20 %
Position	L0.0 A2.3 H2.2 mm
Orientation	Transversal
Phase enc. dir.	R >> L
Slice oversampling	20.0 %
Slices per slab	40
FoV read	240 mm
FoV phase	84.4 %
Slice thickness	3.00 mm
TR	28.0 ms
Multi-slice mode	Interleaved
Series	Interleaved
Concatenations	1

Geometry - AutoAlign

Slab group	1
Position	L0.0 A2.3 H2.2 mm
Orientation	Transversal
Phase enc. dir.	R >> L
AutoAlign	---
Initial Position	L0.0 A2.3 H2.2
L	0.0 mm
A	2.3 mm
H	2.2 mm
Initial Rotation	89.61 deg
Initial Orientation	Transversal

Geometry - Saturation

Saturation mode	Standard
Fat suppr.	None
Water suppr.	None
Special sat.	None

Geometry - Tim Planning Suite

Set-n-Go Protocol	Off
Table position	H

For peer review only

For peer review only - http://bmjopen.bmj.com/site/about/guidelines.xhtml

SIEMENS MAGNETOM Prisma

Geometry - Tim Planning Suite

Table position	0 mm
Inline Composing	Off

System - Miscellaneous

Positioning mode	FIX
Table position	H
Table position	0 mm
MSMA	S - C - T
Sagittal	R >> L
Coronal	A >> P
Transversal	F >> H
Coil Combine Mode	Adaptive Combine
Save uncombined	Off
Matrix Optimization	Off
AutoAlign	---
Coil Select Mode	On - AutoCoilSelect

System - Adjustments

B0 Shim mode	Standard
B1 Shim mode	TrueForm
Adjust with body coil	Off
Confirm freq. adjustment	Off
Assume Dominant Fat	Off
Assume Silicone	Off
Adjustment Tolerance	Auto

System - Adjust Volume

Position	L0.0 A2.3 H2.2 mm
Orientation	Transversal
Rotation	89.61 deg
R >> L	203 mm
A >> P	240 mm
F >> H	120 mm
Reset	Off

System - pTx Volumes

B1 Shim mode	TrueForm
Excitation	Slab-sel.

System - Tx/Rx

Frequency 1H	123.244318 MHz
Correction factor	1
Gain	Low
Img. Scale Cor.	1.000
Reset	Off
? Ref. amplitude 1H	0.000 V

Physio - Signal1

1st Signal/Mode	None
TR	28.0 ms
Concatenations	1
Segments	1

Physio - Cardiac

Tagging	None
Magn. preparation	None
Fat suppr.	None
Dark blood	Off
FoV read	240 mm
FoV phase	84.4 %
Phase resolution	100 %

Physio - PACE

Resp. control	Off
Concatenations	1

Inline - Common

Subtract	Off
Measurements	1
StdDev	Off
Liver registration	Off
Save original images	On

Inline - MIP

MIP-Sag	Off
MIP-Cor	Off
MIP-Tra	Off
MIP-Time	Off
Save original images	On

Inline - Soft Tissue

Wash - In	Off
Wash - Out	Off
TTP	Off
PEI	Off
MIP - time	Off
Measurements	1

Inline - Composing

Inline Composing	Off
Distortion Corr.	Off

Inline - MapIt

Save original images	On
MapIt	None
Flip angle	9 deg
Measurements	1
Contrasts	1
TR	28.0 ms
TE	20.00 ms

Sequence - Part 1

Introduction	On
Dimension	3D
Elliptical scanning	Off
Phase stabilisation	Off
Asymmetric echo	Off
Contrasts	1
Flow comp.	Yes
Multi-slice mode	Interleaved
Bandwidth	120 Hz/Px

Sequence - Part 2

Segments	1
Acoustic noise reduction	Active
RF pulse type	Fast
Gradient mode	Whisper
Excitation	Slab-sel.
RF spoiling	On

Sequence - Assistant

Mode	Off
Allowed delay	30 s

For peer review only

SIEMENS MAGNETOM Prisma

\\Study Protocols\BRAIN\Neonates\Theirworld - E161723\t2_blade_dark-fluid_tra_v3

TA: 3:22 PM: REF Voxel size: 0.9×0.9×3.0 mmPAT: 2 Rel. SNR: 1.00 : qtirB_rr

Properties

Prio recon	Off
Load images to viewer	On
Inline movie	Off
Auto store images	On
Load images to stamp segments	On
Load images to graphic segments	Off
Auto open inline display	Off
Auto close inline display	Off
Start measurement without further preparation	Off
Wait for user to start	Off
Start measurements	Single measurement

Routine

Slice group	1
Slices	40
Dist. factor	0 %
Position	Isocenter
Orientation	Transversal
Phase enc. dir.	R >> L
AutoAlign	---
Phase oversampling	0.0 %
FoV read	240 mm
FoV phase	100.0 %
Slice thickness	3.0 mm
TR	10000.0 ms
TE	130 ms
Averages	1
Concatenations	2
Filter	Prescan Normalize
Coil elements	HEA;HEP

Contrast - Common

TR	10000.0 ms
TE	130 ms
TD	0.0 ms
MTC	Off
Magn. preparation	Slice-sel. IR
TI	2606 ms
Flip angle	130 deg
Fat suppr.	Fat sat.
Fat sat. mode	Strong
Water suppr.	None
Restore magn.	Off
Freeze suppressed tissue	On

Contrast - Dynamic

Averages	1
Averaging mode	Short term
Reconstruction	Magnitude
Measurements	1
Multiple series	Each measurement

Resolution - Common

FoV read	240 mm
FoV phase	100.0 %
Slice thickness	3.0 mm
Base resolution	256
BLADE coverage	100.0 %
Trajectory	BLADE

Resolution - Common

Interpolation	Off

Resolution - iPAT

PAT mode	GRAPPA
Accel. factor PE	2
Ref. lines PE	8
Reference scan mode	Integrated

Resolution - Filter Image

Image Filter	Off
Distortion Corr.	Off
Prescan Normalize	On
Unfiltered images	Off
Normalize	Off
B1 filter	Off

Resolution - Filter Rawdata

Raw filter	Off
Elliptical filter	Off

Geometry - Common

Slice group	1
Slices	40
Dist. factor	0 %
Position	Isocenter
Orientation	Transversal
Phase enc. dir.	R >> L
FoV read	240 mm
FoV phase	100.0 %
Slice thickness	3.0 mm
TR	10000.0 ms
Multi-slice mode	Interleaved
Series	Interleaved
Concatenations	2

Geometry - AutoAlign

Slice group	1
Position	Isocenter
Orientation	Transversal
Phase enc. dir.	R >> L
AutoAlign	---
Initial Position	Isocenter
L	0.0 mm
P	0.0 mm
H	0.0 mm
Initial Rotation	90.00 deg
Initial Orientation	Transversal

Geometry - Saturation

Fat suppr.	Fat sat.
Fat sat. mode	Strong
Water suppr.	None
Restore magn.	Off
Special sat.	Parallel F
Gap	10 mm
Thickness	70 mm

Geometry - Navigator

For peer review only

SIEMENS MAGNETOM Prisma

Geometry - Tim Planning Suite

Set-n-Go Protocol	Off
Table position	H
Table position	0 mm
Inline Composing	Off

System - Miscellaneous

Positioning mode	REF
Table position	H
Table position	0 mm
MSMA	S - C - T
Sagittal	R >> L
Coronal	A >> P
Transversal	F >> H
Coil Combine Mode	Adaptive Combine
Save uncombined	Off
Matrix Optimization	Off
AutoAlign	---
Coil Select Mode	On - AutoCoilSelect

System - Adjustments

B0 Shim mode	Standard
B1 Shim mode	TrueForm
Adjust with body coil	Off
Confirm freq. adjustment	Off
Assume Dominant Fat	Off
Assume Silicone	Off
Adjustment Tolerance	Auto

System - Adjust Volume

Position	Isocenter
Orientation	Transversal
Rotation	90.00 deg
R >> L	240 mm
A >> P	240 mm
F >> H	120 mm
Reset	Off

System - pTx Volumes

B1 Shim mode	TrueForm

System - Tx/Rx

Frequency 1H	123.244318 MHz
Correction factor	1
Gain	High
Img. Scale Cor.	1.000
Reset	Off
? Ref. amplitude 1H	0.000 V

Physio - Signal1

1st Signal/Mode	None
TR	10000.0 ms
Concatenations	2

Physio - Cardiac

Magn. preparation	Slice-sel. IR
TI	2606 ms
Fat suppr.	Fat sat.
Dark blood	Off
FoV read	240 mm
FoV phase	100.0 %
BLADE coverage	100.0 %
Trajectory	BLADE

Physio - PACE

Resp. control	Off
Concatenations	2

Inline - Common

Subtract	Off
Measurements	1
StdDev	Off
Save original images	On

Inline - MIP

MIP-Sag	Off
MIP-Cor	Off
MIP-Tra	Off
MIP-Time	Off
Save original images	On

Inline - Composing

Inline Composing	Off
Distortion Corr.	Off

Sequence - Part 1

Introduction	On
Dimension	2D
Compensate T2 decay	Off
Contrasts	1
Flow comp.	Read
Multi-slice mode	Interleaved
Free echo spacing	Off
Echo spacing	8.64 ms
Bandwidth	362 Hz/Px

Sequence - Part 2

Define	Turbo factor
Echo trains per slice	9
Phase correction	Automatic
Acoustic noise reduction	Active
RF pulse type	Low SAR
Gradient mode	Normal
Hyperecho	Off
WARP	Off
Motion correction	On
Red. EC sensitivity	Off
Turbo factor	28

Sequence - Assistant

Mode	Min flip angle
Min flip angle	130 deg
Allowed delay	30 s

For peer review only

For peer review only - http://bmjopen.bmj.com/site/about/guidelines.xhtml

```
# Author: qspace2siemens.m (Michael Thrippleton), manually edited
into 2 parts
# Source file: ./vector_tables/neonate/04-shells-3-6-64-64.txt
# b-value at UI: 750
# non-zero b-values: 750
# number of non-zero shells: 1
# number of directions per non-zero shell: 64
# number of b=0 volumes: 7
# total number of directions including b0: 71
[directions=71]
normalization = none
coordinatesystem = xyz
comment=bUI: 750, b: 750, Nb0: 7
vector[0] = ( 0.000000, 0.000000, 0.000000 )
vector[1] = ( -0.538981, 0.033731, -0.091439 )
vector[2] = ( -0.000440, 0.429608, 0.339760 )
vector[3] = ( -0.147395, -0.494556, -0.183546 )
vector[4] = ( 0.239035, -0.347062, 0.349872 )
vector[5] = ( -0.016278, -0.195328, 0.511451 )
vector[6] = ( -0.061295, -0.451376, 0.304143 )
vector[7] = ( 0.025626, -0.008709, -0.547053 )
vector[8] = ( -0.231133, -0.471788, 0.154896 )
vector[9] = ( -0.397538, -0.105537, -0.361699 )
vector[10] = ( 0.447399, -0.280126, -0.146162 )
vector[11] = ( 0.000000, 0.000000, 0.000000 )
vector[12] = ( -0.347344, -0.305418, 0.293379 )
vector[13] = ( 0.195148, -0.224679, 0.459823 )
vector[14] = ( 0.219722, 0.401006, -0.301523 )
vector[15] = ( 0.496386, 0.051099, 0.225809 )
vector[16] = ( -0.490022, 0.181524, -0.164098 )
vector[17] = ( 0.415886, 0.250359, 0.253691 )
vector[18] = ( 0.293795, 0.319409, 0.334159 )
vector[19] = ( 0.446457, -0.091032, 0.303955 )
vector[20] = ( 0.218923, -0.268898, -0.423989 )
vector[21] = ( -0.245685, -0.236576, 0.428568 )
vector[22] = ( 0.000000, 0.000000, 0.000000 )
vector[23] = ( 0.023434, -0.514342, -0.186823 )
vector[24] = ( 0.210090, -0.495890, -0.099773 )
vector[25] = ( 0.127918, 0.282591, 0.451419 )
vector[26] = ( -0.497742, -0.190842, -0.125826 )
vector[27] = ( -0.352216, -0.116300, 0.403012 )
vector[28] = ( -0.439047, 0.004691, 0.327438 )
vector[29] = ( 0.143700, -0.138995, -0.509932 )
vector[30] = ( -0.483604, 0.256940, -0.010438 )
vector[31] = ( 0.536886, 0.108072, -0.008594 )
vector[32] = ( -0.113008, -0.337640, 0.416207 )
vector[33] = ( 0.000000, 0.000000, 0.000000 )
vector[34] = ( 0.346021, -0.402459, -0.135263 )
vector[35] = ( -0.172278, 0.446108, 0.267035 )
vector[36] = ( -0.309270, 0.076830, -0.445476 )
vector[37] = ( 0.274066, -0.423055, 0.214272 )
vector[38] = ( 0.052227, -0.321802, 0.440132 )
vector[39] = ( 0.075465, 0.519169, -0.157382 )
vector[40] = ( 0.152874, 0.405328, 0.335170 )
```

For peer review only

```
vector[41] = ( 0.109576, 0.536320, 0.018825 )
vector[42] = ( -0.045652, 0.300780, 0.455464 )
vector[43] = ( 0.000000, 0.000000, 0.000000 )
vector[44] = ( -0.533887, 0.114345, 0.043471 )
vector[45] = ( -0.097529, 0.434255, -0.319235 )
vector[46] = ( 0.391774, -0.236122, -0.301263 )
vector[47] = ( 0.399513, -0.317429, 0.199068 )
vector[48] = ( 0.200167, 0.067226, 0.505385 )
vector[49] = ( 0.385668, -0.387145, 0.037137 )
vector[50] = ( 0.059543, 0.145424, 0.524697 )
vector[51] = ( -0.445546, -0.189946, 0.255752 )
vector[52] = ( 0.263180, -0.007998, -0.480284 )
vector[53] = ( -0.375132, -0.375662, 0.134735 )
vector[54] = ( 0.000000, 0.000000, 0.000000 )
vector[55] = ( -0.100958, 0.513042, -0.163080 )
vector[56] = ( 0.266095, 0.478340, 0.019604 )
vector[57] = ( 0.480516, -0.133538, -0.226434 )
vector[58] = ( 0.253431, -0.482875, 0.051025 )
vector[59] = ( 0.361384, -0.227994, 0.342667 )
vector[60] = ( -0.479164, -0.248769, 0.092279 )
vector[61] = ( -0.422438, -0.343026, -0.062282 )
vector[62] = ( 0.525823, 0.037772, -0.148605 )
vector[63] = ( 0.112166, -0.092301, 0.528109 )
vector[64] = ( 0.050487, -0.545354, 0.006363 )
vector[65] = ( 0.000000, 0.000000, 0.000000 )
vector[66] = ( -0.290577, 0.355116, 0.299095 )
vector[67] = ( -0.303506, -0.415037, -0.188755 )
vector[68] = ( -0.340501, 0.129187, 0.409109 )
vector[69] = ( -0.275521, -0.188617, -0.434179 )
vector[70] = ( 0.148849, 0.097956, -0.517928 )
```

For peer review only

```
# Author: qspace2siemens.m (Michael Thrippleton), manually edited
into 2 parts
# Source file: ./vector_tables/neonate/04-shells-3-6-64-64.txt
# b-value at UI: 2500
# non-zero b-values: 200   500  2500
# number of non-zero shells: 2
# number of directions per non-zero shell: 3   6  64
# number of b=0 volumes: 7
# total number of directions including b0: 151
[directions=80]
normalization = none
coordinatesystem = xyz
comment=bUI: 2500, b: 200   500   2500, Nb0: 7
vector[0] = ( 0.000000, 0.000000, 0.000000 )
vector[1] = ( 0.252007, 0.053675, -0.116668 )
vector[2] = ( 0.118341, -0.013011, 0.256566 )
vector[3] = ( 0.047528, -0.276133, -0.038625 )
vector[4] = ( -0.303298, -0.002700, -0.328638 )
vector[5] = ( -0.128927, -0.159163, 0.397549 )
vector[6] = ( 0.288240, 0.341931, 0.000938 )
vector[7] = ( -0.166829, 0.397185, -0.120052 )
vector[8] = ( -0.069301, 0.303423, 0.321142 )
vector[9] = ( 0.425645, -0.074339, -0.115324 )
vector[10] = ( 0.391424, -0.221918, 0.893051 )
vector[11] = ( 0.458593, -0.241695, -0.855147 )
vector[12] = ( 0.354539, 0.919288, 0.170913 )
vector[13] = ( 0.495263, -0.780339, -0.381819 )
vector[14] = ( -0.574230, 0.458191, 0.678470 )
vector[15] = ( 0.000000, 0.000000, 0.000000 )
vector[16] = ( -0.188453, -0.033220, -0.981520 )
vector[17] = ( 0.594951, -0.772279, 0.222754 )
vector[18] = ( 0.076963, -0.202692, -0.976213 )
vector[19] = ( -0.354234, 0.663631, 0.658872 )
vector[20] = ( -0.245839, 0.923577, 0.294225 )
vector[21] = ( -0.646526, -0.378550, -0.662347 )
vector[22] = ( 0.782685, 0.616196, -0.087788 )
vector[23] = ( -0.102171, -0.675368, -0.730369 )
vector[24] = ( -0.593833, 0.627627, -0.503435 )
vector[25] = ( -0.289839, 0.954652, -0.068065 )
vector[26] = ( 0.000000, 0.000000, 0.000000 )
vector[27] = ( 0.932852, 0.268018, -0.240735 )
vector[28] = ( -0.292661, 0.011816, 0.956143 )
vector[29] = ( -0.125932, -0.877649, -0.462465 )
vector[30] = ( 0.287138, 0.947828, -0.138468 )
vector[31] = ( -0.400507, -0.785392, -0.471967 )
vector[32] = ( 0.046561, 0.178494, -0.982839 )
vector[33] = ( 0.774106, -0.243372, -0.584405 )
vector[34] = ( -0.709331, 0.570685, 0.413724 )
vector[35] = ( 0.258673, -0.649858, 0.714684 )
vector[36] = ( 0.000000, 0.000000, 0.000000 )
vector[37] = ( 0.812504, 0.520520, 0.262482 )
vector[38] = ( -0.551995, -0.116325, -0.825694 )
vector[39] = ( -0.680119, 0.223136, -0.698319 )
vector[40] = ( -0.848362, -0.280672, -0.448893 )
```

For peer review only

```
vector[41] = ( -0.460227, -0.230447, 0.857371 )
vector[42] = ( 0.639224, 0.615748, 0.460703 )
vector[43] = ( 0.953358, -0.285443, 0.098132 )
vector[44] = ( -0.501430, 0.459528, -0.733077 )
vector[45] = ( 0.922461, 0.385130, 0.027209 )
vector[46] = ( -0.815410, 0.546002, -0.192323 )
vector[47] = ( 0.000000, 0.000000, 0.000000 )
vector[48] = ( -0.924442, 0.129694, -0.358591 )
vector[49] = ( 0.549990, 0.820347, -0.156657 )
vector[50] = ( 0.774802, 0.509647, -0.374089 )
vector[51] = ( 0.907672, -0.355700, -0.222731 )
vector[52] = ( 0.051712, 0.985317, 0.162714 )
vector[53] = ( -0.970546, -0.135098, -0.199471 )
vector[54] = ( -0.621107, -0.417526, 0.663249 )
vector[55] = ( -0.776136, 0.621968, 0.103774 )
vector[56] = ( 0.551897, -0.830144, -0.079188 )
vector[57] = ( 0.555009, 0.711394, -0.431142 )
vector[58] = ( 0.000000, 0.000000, 0.000000 )
vector[59] = ( -0.239295, 0.451777, 0.859439 )
vector[60] = ( -0.325801, -0.314211, -0.891698 )
vector[61] = ( 0.649939, -0.012663, -0.759881 )
vector[62] = ( -0.042327, 0.894181, -0.445699 )
vector[63] = ( -0.159022, 0.408833, -0.898648 )
vector[64] = ( 0.388219, 0.606776, -0.693620 )
vector[65] = ( -0.329997, 0.825600, -0.457697 )
vector[66] = ( 0.060764, 0.443276, 0.894323 )
vector[67] = ( -0.794452, 0.390958, -0.464756 )
vector[68] = ( -0.392295, -0.567128, -0.724204 )
vector[69] = ( 0.000000, 0.000000, 0.000000 )
vector[70] = ( 0.272234, 0.851327, -0.448477 )
vector[71] = ( 0.785891, 0.193927, -0.587169 )
vector[72] = ( -0.145787, 0.828569, 0.540573 )
vector[73] = ( 0.616784, 0.765973, 0.181281 )
vector[74] = ( -0.808755, -0.029868, -0.587387 )
vector[75] = ( 0.997247, -0.010658, -0.073384 )
vector[76] = ( -0.152743, -0.477444, 0.865284 )
vector[77] = ( -0.040188, -0.715882, 0.697064 )
vector[78] = ( -0.907740, 0.040990, 0.417525 )
vector[79] = ( 0.008357, -0.985450, 0.169758 )
```

For peer review only

SIEMENS MAGNETOM Prisma

\\Study Protocols\BRAIN\Neonates\Theirworld - E161723 - MT_test\MTSatOn_neonate_v2
TA: 2:58 PM: REF Voxel size: 2.0×2.0×2.0 mmPAT: 3 Rel. SNR: 1.00 : qfl

Properties

Prio recon	Off
Load images to viewer	On
Inline movie	Off
Auto store images	On
Load images to stamp segments	Off
Load images to graphic segments	Off
Auto open inline display	Off
Auto close inline display	Off
Start measurement without further preparation	Off
Wait for user to start	Off
Start measurements	Single measurement

Routine

Slab group	1
Slabs	1
Dist. factor	20 %
Position	R6.7 P19.4 H34.5 mm
Orientation	Sagittal
Phase enc. dir.	A >> P
AutoAlign	---
Phase oversampling	0 %
Slice oversampling	0.0 %
Slices per slab	72
FoV read	128 mm
FoV phase	121.9 %
Slice thickness	2.00 mm
TR	75.0 ms
TE 1	1.54 ms
TE 2	4.55 ms
TE 3	8.56 ms
Averages	1
Concatenations	1
Filter	Prescan Normalize
Coil elements	PeH;PeN

Contrast - Common

TR	75.0 ms
TE 1	1.54 ms
TE 2	4.55 ms
TE 3	8.56 ms
MTC	On
Magn. preparation	None
Flip angle	5 deg
Fat suppr.	None
Water suppr.	None
SWI	Off

Contrast - Dynamic

Averages	1
Averaging mode	Short term
Reconstruction	Magnitude
Measurements	1
Multiple series	Each measurement

Resolution - Common

FoV read	128 mm
FoV phase	121.9 %
Slice thickness	2.00 mm
Base resolution	64

Resolution - Common

Phase resolution	100 %
Slice resolution	100 %
Phase partial Fourier	6/8
Slice partial Fourier	Off
Interpolation	Off

Resolution - iPAT

PAT mode	GRAPPA
Accel. factor PE	3
Ref. lines PE	24
Accel. factor 3D	1
Reference scan mode	Integrated

Resolution - Filter Image

Image Filter	Off
Distortion Corr.	Off
Prescan Normalize	On
Unfiltered images	Off
Normalize	Off
B1 filter	Off

Resolution - Filter Rawdata

Raw filter	Off
Elliptical filter	Off

Geometry - Common

Slab group	1
Slabs	1
Dist. factor	20 %
Position	R6.7 P19.4 H34.5 mm
Orientation	Sagittal
Phase enc. dir.	A >> P
Slice oversampling	0.0 %
Slices per slab	72
FoV read	128 mm
FoV phase	121.9 %
Slice thickness	2.00 mm
TR	75.0 ms
Multi-slice mode	Interleaved
Series	Interleaved
Concatenations	1

Geometry - AutoAlign

Slab group	1
Position	R6.7 P19.4 H34.5 mm
Orientation	Sagittal
Phase enc. dir.	A >> P
AutoAlign	---
Initial Position	R6.7 P19.4 H34.5
R	6.7 mm
P	19.4 mm
H	34.5 mm
Initial Rotation	0.00 deg
Initial Orientation	Sagittal

Geometry - Saturation

Saturation mode	Standard
Fat suppr.	None
Water suppr.	None
Special sat.	None

For peer review only

SIEMENS MAGNETOM Prisma

Geometry - Tim Planning Suite

Set-n-Go Protocol	Off
Table position	H
Table position	0 mm
Inline Composing	Off

System - Miscellaneous

Positioning mode	REF
Table position	H
Table position	0 mm
MSMA	S - C - T
Sagittal	R >> L
Coronal	A >> P
Transversal	F >> H
Coil Combine Mode	Sum of Squares
Save uncombined	Off
Matrix Optimization	Off
AutoAlign	---
Coil Select Mode	Off - AutoCoilSelect

System - Adjustments

B0 Shim mode	Tune up
B1 Shim mode	TrueForm
Adjust with body coil	Off
Confirm freq. adjustment	Off
Assume Dominant Fat	Off
Assume Silicone	Off
Adjustment Tolerance	Auto

System - Adjust Volume

Position	Isocenter
Orientation	Transversal
Rotation	0.00 deg
A >> P	263 mm
R >> L	350 mm
F >> H	350 mm
Reset	Off

System - pTx Volumes

B1 Shim mode	TrueForm
Excitation	Non-sel.

System - Tx/Rx

Frequency 1H	123.244480 MHz
Correction factor	1
Gain	Low
Img. Scale Cor.	3.000
Reset	Off
? Ref. amplitude 1H	0.000 V

Physio - Signal1

1st Signal/Mode	None
TR	75.0 ms
Concatenations	1
Segments	1

Physio - Cardiac

Tagging	None
Magn. preparation	None
Fat suppr.	None
Dark blood	Off
FoV read	128 mm
FoV phase	121.9 %
Phase resolution	100 %

Physio - PACE

Resp. control	Off
Concatenations	1

Inline - Common

Subtract	Off
Measurements	1
StdDev	Off
Liver registration	Off
Save original images	On

Inline - MIP

MIP-Sag	Off
MIP-Cor	Off
MIP-Tra	Off
MIP-Time	Off
Save original images	On

Inline - Soft Tissue

Wash - In	Off
Wash - Out	Off
TTP	Off
PEI	Off
MIP - time	Off
Measurements	1

Inline - Composing

Inline Composing	Off
Distortion Corr.	Off

Inline - MapIt

Save original images	On
MapIt	None
Flip angle	5 deg
Measurements	1
Contrasts	3
TR	75.0 ms
TE 1	1.54 ms
TE 2	4.55 ms
TE 3	8.56 ms

Sequence - Part 1

Introduction	Off
Dimension	3D
Elliptical scanning	On
Phase stabilisation	Off
Asymmetric echo	Off
Contrasts	3
Flow comp. 1	No
Readout mode	Bipolar
Multi-slice mode	Interleaved
Bandwidth 1	580 Hz/Px
Bandwidth 2	580 Hz/Px
Bandwidth 3	580 Hz/Px

Sequence - Part 2

Segments	1
Acoustic noise reduction	Active
RF pulse type	Low SAR
Gradient mode	Normal
Excitation	Non-sel.
RF spoiling	On

For peer review only - http://bmjopen.bmj.com/site/about/guidelines.xhtml

SIEMENS MAGNETOM Prisma

Sequence - Assistant

Mode	Off
Allowed delay	30 s

For peer review only

For peer review only - http://bmjopen.bmj.com/site/about/guidelines.xhtml

SIEMENS MAGNETOM Prisma

\\Study Protocols\BRAIN\Neonates\Theirworld - E161723 - MT_test\MTSatOff_neonate_v2

TA: 2:58 PM: FIX Voxel size: 2.0×2.0×2.0 mmPAT: 3 Rel. SNR: 1.00 : qfl

Properties

Prio recon	Off
Load images to viewer	On
Inline movie	Off
Auto store images	On
Load images to stamp segments	Off
Load images to graphic segments	Off
Auto open inline display	Off
Auto close inline display	Off
Start measurement without further preparation	On
Wait for user to start	Off
Start measurements	Single measurement

Routine

Slab group	1
Slabs	1
Dist. factor	20 %
Position	R6.7 P19.4 H34.5 mm
Orientation	Sagittal
Phase enc. dir.	A >> P
AutoAlign	---
Phase oversampling	0 %
Slice oversampling	0.0 %
Slices per slab	72
FoV read	128 mm
FoV phase	121.9 %
Slice thickness	2.00 mm
TR	75.0 ms
TE 1	1.54 ms
TE 2	4.55 ms
TE 3	8.56 ms
Averages	1
Concatenations	1
Filter	Prescan Normalize
Coil elements	PeH;PeN

Contrast - Common

TR	75.0 ms
TE 1	1.54 ms
TE 2	4.55 ms
TE 3	8.56 ms
MTC	Off
Magn. preparation	None
Flip angle	5 deg
Fat suppr.	None
Water suppr.	None
SWI	Off

Contrast - Dynamic

Averages	1
Averaging mode	Short term
Reconstruction	Magnitude
Measurements	1
Multiple series	Each measurement

Resolution - Common

FoV read	128 mm
FoV phase	121.9 %
Slice thickness	2.00 mm
Base resolution	64

Resolution - Common

Phase resolution	100 %
Slice resolution	100 %
Phase partial Fourier	6/8
Slice partial Fourier	Off
Interpolation	Off

Resolution - iPAT

PAT mode	GRAPPA
Accel. factor PE	3
Ref. lines PE	24
Accel. factor 3D	1
Reference scan mode	Integrated

Resolution - Filter Image

Image Filter	Off
Distortion Corr.	Off
Prescan Normalize	On
Unfiltered images	Off
Normalize	Off
B1 filter	Off

Resolution - Filter Rawdata

Raw filter	Off
Elliptical filter	Off

Geometry - Common

Slab group	1
Slabs	1
Dist. factor	20 %
Position	R6.7 P19.4 H34.5 mm
Orientation	Sagittal
Phase enc. dir.	A >> P
Slice oversampling	0.0 %
Slices per slab	72
FoV read	128 mm
FoV phase	121.9 %
Slice thickness	2.00 mm
TR	75.0 ms
Multi-slice mode	Interleaved
Series	Interleaved
Concatenations	1

Geometry - AutoAlign

Slab group	1
Position	R6.7 P19.4 H34.5 mm
Orientation	Sagittal
Phase enc. dir.	A >> P
AutoAlign	---
Initial Position	R6.7 P19.4 H34.5
R	6.7 mm
P	19.4 mm
H	34.5 mm
Initial Rotation	0.00 deg
Initial Orientation	Sagittal

Geometry - Saturation

Saturation mode	Standard
Fat suppr.	None
Water suppr.	None
Special sat.	None

For peer review only

SIEMENS MAGNETOM Prisma

Geometry - Tim Planning Suite

Set-n-Go Protocol	Off
Table position	H
Table position	0 mm
Inline Composing	Off

System - Miscellaneous

Positioning mode	FIX
Table position	H
Table position	0 mm
MSMA	S - C - T
Sagittal	R >> L
Coronal	A >> P
Transversal	F >> H
Coil Combine Mode	Sum of Squares
Save uncombined	Off
Matrix Optimization	Off
AutoAlign	---
Coil Select Mode	Off - AutoCoilSelect

System - Adjustments

B0 Shim mode	Tune up
B1 Shim mode	TrueForm
Adjust with body coil	Off
Confirm freq. adjustment	Off
Assume Dominant Fat	Off
Assume Silicone	Off
Adjustment Tolerance	Auto

System - Adjust Volume

Position	Isocenter
Orientation	Transversal
Rotation	0.00 deg
A >> P	263 mm
R >> L	350 mm
F >> H	350 mm
Reset	Off

System - pTx Volumes

B1 Shim mode	TrueForm
Excitation	Non-sel.

System - Tx/Rx

Frequency 1H	123.244480 MHz
Correction factor	1
Gain	Low
Img. Scale Cor.	3.000
Reset	Off
? Ref. amplitude 1H	0.000 V

Physio - Signal1

1st Signal/Mode	None
TR	75.0 ms
Concatenations	1
Segments	1

Physio - Cardiac

Tagging	None
Magn. preparation	None
Fat suppr.	None
Dark blood	Off
FoV read	128 mm
FoV phase	121.9 %
Phase resolution	100 %

Physio - PACE

Resp. control	Off
Concatenations	1

Inline - Common

Subtract	Off
Measurements	1
StdDev	Off
Liver registration	Off
Save original images	On

Inline - MIP

MIP-Sag	Off
MIP-Cor	Off
MIP-Tra	Off
MIP-Time	Off
Save original images	On

Inline - Soft Tissue

Wash - In	Off
Wash - Out	Off
TTP	Off
PEI	Off
MIP - time	Off
Measurements	1

Inline - Composing

Inline Composing	Off
Distortion Corr.	Off

Inline - MapIt

Save original images	On
MapIt	None
Flip angle	5 deg
Measurements	1
Contrasts	3
TR	75.0 ms
TE 1	1.54 ms
TE 2	4.55 ms
TE 3	8.56 ms

Sequence - Part 1

Introduction	Off
Dimension	3D
Elliptical scanning	On
Phase stabilisation	Off
Asymmetric echo	Off
Contrasts	3
Flow comp. 1	No
Readout mode	Bipolar
Multi-slice mode	Interleaved
Bandwidth 1	580 Hz/Px
Bandwidth 2	580 Hz/Px
Bandwidth 3	580 Hz/Px

Sequence - Part 2

Segments	1
Acoustic noise reduction	Active
RF pulse type	Low SAR
Gradient mode	Normal
Excitation	Non-sel.
RF spoiling	On

For peer review only

SIEMENS MAGNETOM Prisma

Sequence - Assistant

Mode	Off
Allowed delay	30 s

For peer review only

SIEMENS MAGNETOM Prisma

\\Study Protocols\BRAIN\Neonates\Theirworld - E161723 - MT_test\MTSatT1_neonate_v2

TA: 0:36 PM: FIX Voxel size: 2.0×2.0×2.0 mmPAT: 3 Rel. SNR: 1.00 : qfl

Properties

Prio recon	Off
Load images to viewer	On
Inline movie	Off
Auto store images	On
Load images to stamp segments	Off
Load images to graphic segments	Off
Auto open inline display	Off
Auto close inline display	Off
Start measurement without further preparation	On
Wait for user to start	Off
Start measurements	Single measurement

Routine

Slab group	1
Slabs	1
Dist. factor	20 %
Position	R6.7 P19.4 H34.5 mm
Orientation	Sagittal
Phase enc. dir.	A >> P
AutoAlign	---
Phase oversampling	0 %
Slice oversampling	0.0 %
Slices per slab	72
FoV read	128 mm
FoV phase	121.9 %
Slice thickness	2.00 mm
TR	15.0 ms
TE 1	1.54 ms
TE 2	4.55 ms
TE 3	8.56 ms
Averages	1
Concatenations	1
Filter	Prescan Normalize
Coil elements	PeH;PeN

Contrast - Common

TR	15.0 ms
TE 1	1.54 ms
TE 2	4.55 ms
TE 3	8.56 ms
MTC	Off
Magn. preparation	None
Flip angle	14 deg
Fat suppr.	None
Water suppr.	None
SWI	Off

Contrast - Dynamic

Averages	1
Averaging mode	Short term
Reconstruction	Magnitude
Measurements	1
Multiple series	Each measurement

Resolution - Common

FoV read	128 mm
FoV phase	121.9 %
Slice thickness	2.00 mm
Base resolution	64

Resolution - Common

Phase resolution	100 %
Slice resolution	100 %
Phase partial Fourier	6/8
Slice partial Fourier	Off
Interpolation	Off

Resolution - iPAT

PAT mode	GRAPPA
Accel. factor PE	3
Ref. lines PE	24
Accel. factor 3D	1
Reference scan mode	Integrated

Resolution - Filter Image

Image Filter	Off
Distortion Corr.	Off
Prescan Normalize	On
Unfiltered images	Off
Normalize	Off
B1 filter	Off

Resolution - Filter Rawdata

Raw filter	Off
Elliptical filter	Off

Geometry - Common

Slab group	1
Slabs	1
Dist. factor	20 %
Position	R6.7 P19.4 H34.5 mm
Orientation	Sagittal
Phase enc. dir.	A >> P
Slice oversampling	0.0 %
Slices per slab	72
FoV read	128 mm
FoV phase	121.9 %
Slice thickness	2.00 mm
TR	15.0 ms
Multi-slice mode	Interleaved
Series	Interleaved
Concatenations	1

Geometry - AutoAlign

Slab group	1
Position	R6.7 P19.4 H34.5 mm
Orientation	Sagittal
Phase enc. dir.	A >> P
AutoAlign	---
Initial Position	R6.7 P19.4 H34.5
R	6.7 mm
P	19.4 mm
H	34.5 mm
Initial Rotation	0.00 deg
Initial Orientation	Sagittal

Geometry - Saturation

Saturation mode	Standard
Fat suppr.	None
Water suppr.	None
Special sat.	None

For peer review only

SIEMENS MAGNETOM Prisma

Geometry - Tim Planning Suite

Set-n-Go Protocol	Off
Table position	H
Table position	0 mm
Inline Composing	Off

System - Miscellaneous

Positioning mode	FIX
Table position	H
Table position	0 mm
MSMA	S - C - T
Sagittal	R >> L
Coronal	A >> P
Transversal	F >> H
Coil Combine Mode	Sum of Squares
Save uncombined	Off
Matrix Optimization	Off
AutoAlign	---
Coil Select Mode	Off - AutoCoilSelect

System - Adjustments

B0 Shim mode	Tune up
B1 Shim mode	TrueForm
Adjust with body coil	Off
Confirm freq. adjustment	Off
Assume Dominant Fat	Off
Assume Silicone	Off
Adjustment Tolerance	Auto

System - Adjust Volume

Position	Isocenter
Orientation	Transversal
Rotation	0.00 deg
A >> P	263 mm
R >> L	350 mm
F >> H	350 mm
Reset	Off

System - pTx Volumes

B1 Shim mode	TrueForm
Excitation	Non-sel.

System - Tx/Rx

Frequency 1H	123.244480 MHz
Correction factor	1
Gain	Low
Img. Scale Cor.	3.000
Reset	Off
? Ref. amplitude 1H	0.000 V

Physio - Signal1

1st Signal/Mode	None
TR	15.0 ms
Concatenations	1
Segments	1

Physio - Cardiac

Tagging	None
Magn. preparation	None
Fat suppr.	None
Dark blood	Off
FoV read	128 mm
FoV phase	121.9 %
Phase resolution	100 %

Physio - PACE

Resp. control	Off
Concatenations	1

Inline - Common

Subtract	Off
Measurements	1
StdDev	Off
Liver registration	Off
Save original images	On

Inline - MIP

MIP-Sag	Off
MIP-Cor	Off
MIP-Tra	Off
MIP-Time	Off
Save original images	On

Inline - Soft Tissue

Wash - In	Off
Wash - Out	Off
TTP	Off
PEI	Off
MIP - time	Off
Measurements	1

Inline - Composing

Inline Composing	Off
Distortion Corr.	Off

Inline - MapIt

Save original images	On
MapIt	None
Flip angle	14 deg
Measurements	1
Contrasts	3
TR	15.0 ms
TE 1	1.54 ms
TE 2	4.55 ms
TE 3	8.56 ms

Sequence - Part 1

Introduction	Off
Dimension	3D
Elliptical scanning	On
Phase stabilisation	Off
Asymmetric echo	Off
Contrasts	3
Flow comp. 1	No
Readout mode	Bipolar
Multi-slice mode	Interleaved
Bandwidth 1	580 Hz/Px
Bandwidth 2	580 Hz/Px
Bandwidth 3	580 Hz/Px

Sequence - Part 2

Segments	1
Acoustic noise reduction	Active
RF pulse type	Low SAR
Gradient mode	Normal
Excitation	Non-sel.
RF spoiling	On

For peer review only

SIEMENS MAGNETOM Prisma

Sequence - Assistant

Mode	Off
Allowed delay	30 s

For peer review only

BMJ Open

Impact of preterm birth on brain development and long-term outcome: protocol for a cohort study in Scotland

Journal:	*BMJ Open*
Manuscript ID	bmjopen-2019-035854.R1
Article Type:	Protocol
Date Submitted by the Author:	16-Jan-2020
Complete List of Authors:	Boardman, James; The University of Edinburgh, MRC Centre for Reproductive Health; The University of Edinburgh, Centre for Clinical Brain Sciences<br>Hall, Jill; The University of Edinburgh, MRC Centre for Reproductive Health<br>Thrippleton, Michael; The University of Edinburgh, Edinburgh Imaging<br>Reynolds, Rebecca; The University of Edinburgh, Centre for Cardiovascular Science<br>Bogaert, Debby; The University of Edinburgh, Centre for Inflammation Research<br>Davidson, Donald ; The University of Edinburgh, Centre for Inflammation Research<br>Schwarze, Jurgen; The University of Edinburgh, Centre for Inflammation Research<br>Drake, Amanda; The University of Edinburgh, Centre for Cardiovascular Sciences<br>Chandran, Siddharthan ; The University of Edinburgh, Centre for Clinical Brain Sciences<br>Bastin, Mark; The University of Edinburgh, Centre for Clinical Brain Sciences<br>Fletcher-Watson, Sue; The University of Edinburgh, Centre for Clinical Brain Sciences
**Primary Subject Heading**:	Paediatrics
Secondary Subject Heading:	Reproductive medicine, Radiology and imaging, Obstetrics and gynaecology, Neurology, Intensive care
Keywords:	Neonatal intensive & critical care < INTENSIVE & CRITICAL CARE, Paediatric neurology < NEUROLOGY, Maternal medicine < OBSTETRICS, Developmental neurology & neurodisability < PAEDIATRICS, Child & adolescent psychiatry < PSYCHIATRY, Magnetic resonance imaging < RADIOLOGY & IMAGING

I, the Submitting Author has the right to grant and does grant on behalf of all authors of the Work (as defined in the below author licence), an exclusive licence and/or a non-exclusive licence for contributions from authors who are: i) UK Crown employees; ii) where BMJ has agreed a CC-BY licence shall apply, and/or iii) in accordance with the terms applicable for US Federal Government officers or employees acting as part of their official duties; on a worldwide, perpetual, irrevocable, royalty-free basis to BMJ Publishing Group Ltd ("BMJ") its licensees and where the relevant Journal is co-owned by BMJ to the co-owners of the Journal, to publish the Work in this journal and any other BMJ products and to exploit all rights, as set out in our licence.

The Submitting Author accepts and understands that any supply made under these terms is made by BMJ to the Submitting Author unless you are acting as an employee on behalf of your employer or a postgraduate student of an affiliated institution which is paying any applicable article publishing charge ("APC") for Open Access articles. Where the Submitting Author wishes to make the Work available on an Open Access basis (and intends to pay the relevant APC), the terms of reuse of such Open Access shall be governed by a Creative Commons licence – details of these licences and which Creative Commons licence will apply to this Work are set out in our licence referred to above.

Other than as permitted in any relevant BMJ Author's Self Archiving Policies, I confirm this Work has not been accepted for publication elsewhere, is not being considered for publication elsewhere and does not duplicate material already published. I confirm all authors consent to publication of this Work and authorise the granting of this licence.

For peer review only

Impact of preterm birth on brain development and long-term outcome: protocol for a cohort study in Scotland

James P Boardman[1,2], Jill Hall[1], Michael J Thrippleton[3], Rebecca M Reynolds[4], Debby Bogaert[5], Donald J. Davidson[5], Jürgen Schwarze[5], Amanda J Drake[4], Siddharthan Chandran[2], Mark E Bastin[2], Sue Fletcher-Watson[2]

[1] Medical Research Centre for Reproductive Health, University of Edinburgh, Edinburgh, UK

[2] Centre for Clinical Brain Sciences, University of Edinburgh, Edinburgh, UK

[3] Edinburgh Imaging, University of Edinburgh, UK

[4]Centre for Cardiovascular Science, University of Edinburgh, Edinburgh, UK

[5] University of Edinburgh Centre for Inflammation Research, Edinburgh, UK

Corresponding author:

Professor James Peter Boardman

W1.26 Queen's Medical Research Institute,

47 Little France Crescent,

Edinburgh EH16 4TJ,

United Kingdom.

T: +44 131 242 2567

E: james.boardman@ed.ac.uk

Word count (excl title page, abstract, references, figures and tables): 3,538

For peer review only

Abstract

Introduction. Preterm birth is closely associated with altered brain development and is a leading cause of neurodevelopmental, cognitive and behavioural impairment across the life course. We aim to investigate neuroanatomic variation and adverse outcomes associated with preterm birth by studying a cohort of preterm infants and controls born at term, using brain magnetic resonance imaging (MRI) linked to biosamples and clinical, environmental and neuropsychological data.

Methods and Analysis. Theirworld Edinburgh Birth Cohort is a prospective longitudinal cohort study at the University of Edinburgh. We plan to recruit 300 infants born at <33 weeks gestational age (GA) and 100 healthy control infants born after 37 weeks GA. Multiple domains are assessed: maternal and infant clinical and demographic information; placental histology; immunoregulatory and trophic proteins in umbilical cord and neonatal blood; brain macro- and microstructure from structural and diffusion MRI; DNA methylation; hypothalamic-pituitary-adrenal axis (HPAA) activity; social cognition, attention and processing speed from eye-tracking during infancy and childhood; neurodevelopment; gut and respiratory microbiota; susceptibility to viral infections; and participant experience. Main analyses include creation of novel methods for extracting information from neonatal structural and diffusion MRI, regression analyses of predictors of brain maldevelopment and neurocognitive outcome associated with preterm birth, and determination of the quantitative predictive performance of MRI and other early life factors for childhood outcome.

Ethics and Dissemination. Ethical approval has been obtained from the National Research Ethics Service, South East Scotland Research Ethics Committee and NHS Lothian Research and Development. Results are disseminated through open access journals, scientific meetings, social media, newsletters, a study website (www.tebc.ed.ac.uk), and we engage with the University of Edinburgh public relations and media office to ensure maximum publicity and benefit.

For peer review only

Strengths and limitations of this study

- Three hundred preterm infants and a comparator group of 100 term controls are studied longitudinally from before birth to school age.
- Phenotypic information includes data from brain MRI, biosamples, participant report, direct observation and clinical data from maternal and infant medical records.
- We collected data about a range of theoretically informed variables to understand the impact of preterm birth on everyday lives of families.
- A data access and collaboration policy sets out the terms and conditions on which deidentified data are available to the research community.
- Participants are recruited from a single centre.

For peer review only

INTRODUCTION

Preterm delivery is estimated to affect 10.6% of all live births around the world, which equates to 14.84 million births per annum[1]. In resource rich settings advances in perinatal care and service delivery have led to improved survival over the past two decades: around 30% of infants born at 22 weeks who are offered stabilisation at birth will survive, and this number increases to around 80% for births at 26 weeks[2-5]. However, early exposure to extrauterine life can impact brain development, and is closely associated with long term intellectual disability, cerebral palsy, autism spectrum disorder, attention deficit hyperactivity disorder, psychiatric disease, and problems with language, behaviour, and socioemotional function (for review [6]). There are no treatments that reduce risk of impairment, which extends across the life course and carries considerable personal cost to affected individuals, and high health and education costs to society[7].

Little is known about the ontogenesis of neurocognitive and psychiatric problems associated with preterm birth, or the biological, environmental and social risk factors associated with susceptibility and resilience. Much information about the cerebral effects of preterm birth comes from historic cohorts that do not reflect modern perinatal care practices; studies have been cross-sectional with outcomes assessed in very early childhood before important cognitive and social functions emerge; conventional diagnostic tools for assessing neurodevelopment are imprecise; and cohorts linked to imaging and biological metadata are few so mechanisms are poorly understood. There is an unmet need to study a contemporary cohort of preterm infants that is comprehensively characterised from genes to anatomy to function, integrated with information about the social graph.

Our aims are: first, to build a longitudinal cohort of preterm infants and term controls that is phenotyped with brain imaging and biological information to investigate causal pathways to, and consequences of, atypical brain development and injury; second, to develop novel computational algorithms for mapping brain growth and connectivity in early life; third, to identify new and multi-factorial methods for early detection of children at risk of long-term impairment; and fourth, to identify early life biological and environmental risk and resilience factors that affect the developing brain and so pave the way for new therapeutic strategies.

For peer review only

METHODS AND ANALYSIS

Study design

Single-centre prospective longitudinal cohort study.

Study setting

The Theirworld Edinburgh Birth Cohort ("TEBC") study is conducted at the University of Edinburgh and the Simpson Centre for Reproductive Health (SCRH) which is located at the Royal Infirmary of Edinburgh, NHS Lothian, UK. The SCRH provides maternity and newborn services for residents of the City of Edinburgh and the Lothians. It receives 7,000 deliveries per annum and is the regional centre for all neonatal intensive care in South East Scotland. Approximately 100 infants with birthweight <1500g receive intensive care at SCRH per annum.

Participant recruitment, initial assessment and data collection points 1-3 (Table 1) take place in the SCRH or the Edinburgh Imaging Facility, Royal Infirmary of Edinburgh. Follow-up assessments take place in a dedicated child development laboratory at the University of Edinburgh, through online and in-person completion of questionnaires, and in Neonatal Out-patient clinics at the SCRH (timepoints 4-7, Table1). Recruitment began in November 2016 and is planned to complete in 2021.

Study participants

Inclusion criteria

Cases: 300 preterm infants born at <33 weeks gestational age (GA)*.

Controls: 100 term infants born at >37 weeks GA*.

*GA is estimated based on first trimester ultrasound.

Cases are included if a mother booked her pregnancy and delivered at SCRH (the study centre), or if a mother booked her pregnancy at a hospital outside the study centre but was transferred to it with her baby *in utero* due to planned or expected birth <33 weeks. Preterm infants who are transferred to SCRH *ex utero* for intensive care are not included.

Exclusion criteria

1. Infants with congenital anomalies: structural or functional anomalies (e.g. metabolic disorders) that occur during intrauterine life and can be identified prenatally, at birth or later in life (World Health Organisation definition).
2. Infants with a contraindication to MRI at 3 Tesla.

Sample selection and recruitment

For peer review only

Sample size

A key aim of the study is to investigate causes and consequences of preterm brain injury / atypical development by analysing data about brain macro- and microstructure from structural and quantitative MRI with biological, environmental and neuropsychological outcome data. In the absence of established methodology for power calculations using quantitative MRI techniques, the sample size is based on: exemplars of indicative sensitivity and power from computational modelling and previous data; and realistic assessment of recruitment, successful image acquisition of 85%, and follow-up. Studies indicate it is possible to detect group-wise differences in brain anatomy associated with specific exposures by applying computational techniques to MRI data from relatively small group sizes in univariate models: for example Tract-based Spatial Statistics (TBSS) and Network-based Statistics (NBS) are sensitive to generalised changes microstructure and connectivity with 20-60 infants per group[8-14], and morphometric methods detect anatomic variation with similar group sizes, depending on the image feature of interest[15 16]. However, a key strength of the study is that larger samples (n=300-400) are required to construct multivariate models (needed to investigate multiple exposures that influence brain development), to combine information from different MRI modalities using data-driven methods, to investigate associations between image phenotypes and behavioural outcomes which often require larger study populations[17 18], and to develop analytic methods that support causal inference. Another aim is the development of novel computational methods for mapping growth and connectivity in development. While certain technical developments such as image segmentation and methods for studying crossing fibres are achievable with sample sizes of <100[19-22], larger sample sizes are needed to address other challenges. For example, larger atlases of the developing brain than are currently available are required to understand population diversity, and machine learning methods are being used to develop image biomarkers, and to improve the interoperability of multi-site acquisitions, which will enable researchers to increase study power, carry out essential replication studies, and investigate risk and resilience in brain development conferred by the genome[23-25]. We expect to address some of these issues with the planned sample of 400, and to make material contribution to wider data-sharing initiatives subject to the study's Data Access and Collaboration policy.

For peer review only

Identifying participants

Cases: Infants born to women who present to the SCRH with threatened preterm labour and for whom delivery is planned or expected at less than 32 completed weeks GA.

Controls: Infants born to women who attend the SCRH and deliver at >36 weeks GA.

The protocol reported here was partially developed through a separate, pilot 'phase 1' cohort of 150 cases and 40 controls. This phase 1 pilot included neonatal MRI and infant-eye-tracking, and a subset of this group are now participating in the 5-year assessment as described here (time point 7, table 1).

Screening for eligibility

The research nurse / clinical research fellow identifies potential participants using maternity TRAK, which is a system used by maternity services throughout NHS Lothian to record information about pregnancies and maternal care, and the neonatal electronic patient record. The clinical team provides an introductory leaflet about TEBC to eligible parents, and then informs the research team of parents who wish to discuss the study in greater detail. Those parents meet with a member of the research team and are provided with the Participant Information Sheet.

Participants from phase 1 studies being recalled for time point 7 (at 5 years) are contacted by the research team using contact details provided previously. Study information (introductory letter, patient information sheet, reply slip and prepaid envelope) is sent by post and followed up with a telephone call to answer any questions and review willingness to participate.

Consenting participants

Informed written consent is sought in two stages: first, consent for perinatal and neonatal sampling and assessment at initial enrolment to the study; second, consent for assessments post-discharge to 5 years is taken at time point 3 (see Table 1 below).

For phase 1 participants being recalled, consent is taken at the recall appointment, following circulation and discussion of the content by post and phone, as described above.

Informed consent may only be taken by a member of the research team with training in International Council for Harmonisation-Good Clinical Practice (ICH-GCP) and procedures for research involving children and young people.

Co-enrolment

The SCRH is an academic perinatal medicine centre that hosts observational research studies, and it is a recruiting centre for randomised controlled trials of therapies designed to improve

For peer review only

the outcome of preterm infants and their mothers. Parents / carers of TEBC participants are encouraged to consider entry into such studies if eligible. Co-enrolment is informed by 'Guidelines for Co-enrolment' produced by the Academic and Clinical Central Office for Research and Development (ACCORD), which is a partnership between the University of Edinburgh and NHS Lothian Health Board. Co-enrolment will be recorded.

Cohort retention

Participants and their families are kept up to date with research progress through Newsletters, Twitter, Facebook and a website (www.tebc.ed.ac.uk). Birthday cards are sent to participants and we hold an annual event for research updates and public outreach.

Withdrawal of study participants

The decision to withdraw from the study is either at parental / carer request, or at the request of the attending consultant physician or the PI for clinical reasons.

Outcomes and data analysis

Table 1 summarises the assessment schedule, data collection methods, sample type / domain, and the test or task. Data from cases and controls are collected using the same data collection instruments.

For peer review only

Time point	Age	Data collection method	Sample type / domain of measurement	Test / task
1	Antenatal	Records & interview	Socio-economic status	Maternal & paternal education, Scottish Index of Multiple Deprivation derived from home postcode
			Medical / demographic	Family and medical history and exposures
2	Birth	Records, questionnaire & tissue	Medical	History and exposures
				Anthropometry
			Placenta	Structured histopathology rating and storage
			Cord blood	Panel of immunoregulatory and trophic proteins<br>Gene expression array*
3	Neonatal	Tissue: blood	Blood spot	Panel of immunoregulatory and trophic proteins<br>Gene expression array*
		Tissue: saliva	Epigenetics	DNA methylation
		Tissue: nasal swab	Nasal lining fluid	Antimicrobial peptides including cathelicidin levels*
			DNA/RNA	Respiratory microbiota*
		Stool	DNA/RNA	Gut microbiota*
		Direct observation	Medical	Anthropometry
			ROP assessment	Grade retinopathy
			Parent IQ	National Adult Reading Test
		MRI	Brain structure and connectivity	Structural and diffusion 3T MRI
		Questionnaire	Medical / demographic	Breast-feeding and updated perinatal medical history
				Edinburgh Post-natal Depression Scale
				Parenting Daily Hassles
				World Health Organisation – Quality Of Life
				Adult Temperament Questionnaire
4	4.5 months	Questionnaire, by post or online or phone interview	Demographics	Updated Socio-economic status, maternal education, breastfeeding / nutrition, activities
			Infant temperament	Infant Behaviour Questionnaire, Revised, short form
			Parent wellbeing	Edinburgh Post-natal Depression Scale
				World Health Organisation – Quality Of Life
		Tissue: nasal swab	DNA/RNA	Respiratory microbiota*
5	9 months	Tissue: saliva	Epigenetics	DNAm
			HPA axis	Cortisol: Waking, 30 minutes after waking, before bed.<br>Pre and post Still Face procedure
		Tissue: nasal swab	Nasal lining fluid	Antimicrobial peptides including cathelicidin levels*
			DNA/RNA	Respiratory microbiota*
		Eye-tracking	Social development	Free scanning: neutral faces
				Free scanning: “pop-out” task, looking to faces and distractors
				Free scanning: “social preferential looking” to social and non-social images

For peer review only

				Free scanning: "dancing ladies" social and non-social videos
			Attention	Switching and disengagement: "gap-overlap" task, fixation to central and peripheral cues
				Sustained attention: "follow the bird" task, following moving target
			Processing speed	Free scanning: odd-one-out visual search task (simple letters version)
				Free-scanning: word-picture matching task
		**Direct observation**	Visual acuity	Keeler card assessment
			Social development	Still Face procedure (sub-set with computational motor assessment)
				Parent-child play, for later behavioural coding: (sub-set with computational motor assessment)
		**Questionnaire**	Infant temperament	Infant Behaviour Questionnaire, Revised, short form
				Sleep & Settle Questionnaire
			Language	MacArthur Communicative Development Inventory (words and gestures)
			Parent wellbeing	World Health Organisation – Quality Of Life
			Feedback	Feedback form, monitoring satisfaction with research project
		**Direct observation**	Anthropometry	Growth
		**Parent interview**	Demographics	Family circumstances update form including breastfeeding, socio-economic status (home postcode)
			Developmental level	Vineland Adaptive Behaviour Scales: comprehensive interview form
**6**	**2 years**	**Direct observation**	Ophthalmology	Refraction
			Anthropometry	Growth
		**Tissue: nasal swab**	Nasal lining fluid	Antimicrobial peptides including cathelicidin levels*
			DNA/RNA	Respiratory microbiota*
		**Eye-tracking**	Social development	Free scanning: neutral faces
				Free scanning: "pop-out" task, looking to faces and distractors
				Free scanning: "social preferential looking" to social and non-social images
				Free scanning: "dancing ladies" social and non-social videos
			Attention	Switching and disengagement: "gap-overlap" task, fixation to central and peripheral cues
				Sustained attention: "follow the bird" task, following moving target
			Processing speed	Free scanning: odd-one-out visual search task
				Free-scanning: word-picture matching task
		**Direct observation**	Social development	Parent-child play, for later behavioural coding
			Executive function	Following Instructions task
			Bayley-III	General developmental level*
		**Questionnaire**	Temperament	Early Childhood Behaviour Questionnaire, Revised, short form
				Child Sleep Habits Questionnaire
			Language	MacArthur Communicative Development Inventory (words and sentences)
			Social development	Quantitative Checklist for Autism in Toddlers
			Executive function	Behaviour Rating Inventory for Executive Function, Pre-school (BRIEF-P)<br>Early Executive Function Questionnaire
			Developmental level	Vineland Adaptive Behaviour Scales: comprehensive parent rating form

For peer review only

			Parent wellbeing	World Health Organisation – Quality Of Life
			Feedback	Feedback form, monitoring satisfaction with research project
		**Parent interview**	Demographics	Family circumstances update form including breastfeeding, socio-economic status (home postcode)
**7**	**5 years**	**Tissue: saliva**	Epigenetics	DNA methylation
			HPA axis	Cortisol
		**Tissue: nasal swab**	DNA/RNA	Respiratory microbiota*
		**Direct observation**	Anthropometry	Growth
			Blood pressure	Hypertension
			Ophthalmology	Refraction and acuity
			Social development	Parent-child play, for later behavioural coding
			Executive function	Following Instructions task
			Developmental level	Mullen Scales of Early Learning
		**Eye-tracking**	Social development	Free scanning: neutral faces
				Free scanning: “pop-out” task, looking to faces and distractors
				Free scanning: “social preferential looking” to social and non-social images
				Free scanning: “dancing ladies” social and non-social videos
			Attention	Switching and disengagement: “gap-overlap” task, fixation to central and peripheral cues
				Sustained attention: “follow the bird” task, following moving target
			Processing speed	Free scanning: odd-one-out visual search task (complex objects version)
		**Questionnaire**	Temperament	Strengths and Difficulties Questionnaire (both teacher and parent report versions)
			Language	Children's Communication Checklist
			Social development	Social Communication Questionnaire: Current
			Executive function	DUPaul ADHD rating scale
				Behaviour Rating Inventory for Executive Function -Pre-school (BRIEF-P)
			Visual perception	Cerebral Visual Impairment Inventory
			Parent wellbeing	World Health Organisation – Quality Of Life
			Feedback	Feedback form monitoring satisfaction with research project
			Developmental level	Vineland Adaptive Behaviour Scales: domain-level parent rating form
		**Parent interview**	Demographics	Family circumstances update form including socio-economic status (home postcode)

Table 1. Schedule of assessments, data collection methods, sample type / domain, and the test or task.
*subset of participants

For peer review only

Maternal and infant clinical and demographic information

Data are abstracted from the mothers' and infants' electronic medical records onto a standardised data collection sheet. A structured maternal interview is used to collect additional information that may not be recorded in routinely collected data, for example detailed family history about neurodevelopmental and mental health problems, and over-the-counter prescription and recreational drugs taken during pregnancy. For deaths the cause and post-mortem findings will be recorded.

Placentas

After delivery, placentae from all preterm infants are formalin fixed and stored at 4°C before sampling. The placentae are sampled according to a standardized protocol; distal and proximal sections of cord (the proximal section being taken at 1.5 cm from above the fetal surface), a roll of extraplacental membranes starting at the point of rupture and 4 full thickness sections from each quadrant. All are stained with Haematoxylin and Eosin and reported using a standardised, structured approach that describes any pathological features present, including but not limited to, fetal thrombotic vasculopathy, villitis, chorioamnionitis, funisitis and features of uteroplacental ischaemia[12 26].

Immunoregulatory and trophic proteins

Analysis of a panel of immunoregulatory and trophic proteins (IL-1b, IL-2, IL-4, IL-5, IL-6, IL-8, IL-12, IL-17, TNF-a, MIP-1b, BDNF, GM-CSF, IL-10, IL-18, IFN-g, TNF-b, MCP-1, MIP-1a, C3, C5a, C9, MMP-9, RANTES and CRP) is undertaken on umbilical cord and neonatal blood samples. These proteins are selected to offer information with respect to the pro- and anti-inflammatory innate response as well as the adaptive immune response. Blood is collected using Schleicher and Schuell 903 filter paper (6 x 3.2mm spots per subject) and analysed using a multiplex immunoassay (Meso Scale Discovery) at Statens Serum Institute, Copenhagen. We use the approach described by Skogstrand et al[27] to analyse differences in concentration between cases and controls.

Structural and diffusion magnetic resonance imaging

A Siemens MAGNETOM Prisma 3T MRI clinical scanner (Siemens Healthcare, Erlangen, Germany) and 16-channel phased-array paediatric head receive coil is used to acquire: 3D T1-weighted MPRAGE (T1w) structural volume scan (acquired voxel size = 1 mm isotropic) with TI 1100 ms, TE 4.69 ms and TR 1970 ms; a 3D T2-weighted SPACE (T2w) structural scan (voxel size = 1mm isotropic) with TE 409 ms and TR 3200 ms; and a multi-shell axial dMRI scan (16 ×

b = 0 s/mm^2, 3 × b = 200 s/mm^2, 6 × b = 500 s/mm^2, 64 × b = 750 s/mm^2, 64 × b = 2500 s/mm^2) with optimal angular coverage[28] (see Supplementary material 1-3). If the infant stays settled axial 3D susceptibility weighted imaging (SWI; TR = 28 ms, TE = 20 ms, 0.75 x 0.75 x 3 mm acquired resolution) and axial 2D fluid-attenuated inversion-recovery BLADE imaging (FLAIR; TR = 10000 ms, TE = 130 ms, TI = 2606 ms, 0.94 x 0.94 x 3 mm acquired resolution) are acquired. In a subgroup of participants magnetisation transfer saturation imaging is acquired for evaluation of tissue myelin content, consisting of three sagittal 3D multi-echo spoiled gradient echo scans (TE = {1.54 ms, 4.55 ms, 8.56 ms}, 2-mm isotropic acquired resolution): magnetisation-transfer and proton-density weighted (TR = 75 ms, FA = 5°), and T1-weighted (TR = 15 ms, FA = 14°) acquisitions, supplementary material 4. Tissue heating and acoustic noise exposure are limited throughout the examination through the use of active noise cancellation and by setting the gradient slew rate and other pulse sequence parameters appropriately. Participants are scanned in normal mode with respect to both tissue heating and peripheral nerve stimulation.

Conventional images are reported by a paediatric radiologist using a structured system [29, 30]. We use image data to generate novel processing techniques optimised for neonatal data[11 19-21 31], and we will use these and other publicly available pipelines for processing neonatal data[13 32 33] to derive image features for analyses with collateral data relating to exposures and outcomes. These include but are not limited to tract-based, morphometric and structural connectivity analyses [10-12 34-38].

DNA storage

DNA is extracted form saliva, stored and catalogued at the Edinburgh Clinical Research Facility, ready for downstream analyses.

DNA methylation

Saliva is sampled using the DNA OG-575 kit (DNAGenotek, Ottawa, ON, Canada). DNA extraction is performed using published methods[36] and DNAm analyses are carried out at the Genetics Core of the Edinburgh Clinical Research Facility (Edinburgh, UK), using Illumina Infinium MethylationEPIC (San Diego, CA, USA), with interrogation of the arrays against ~850k methylation sites. We will investigate perinatal influences on DNAm using principal component analysis, mediation, and correlation analyses.

For peer review only

Hypothalamic-pituitary-adrenal axis (HPAA)

Salivary cortisol is used as a marker of HPAA activity. Saliva is collected in Sarstedt tubes at specified times at 9 months and 5 years. Timed saliva samples are also collected during the 9 months appointment before and after a behavioural paradigm (Still Face) which is known to elicit a biological stress response (one sample pretest and two samples post test to capture reaction and recovery). Samples are stored at -20C and analysed in batches at each time point. Anthropometric data are recorded at 9 months, 2 years and 5 years, and blood pressure is measured at 5 years.

Eye-tracking

We record eye-movements in response to visual stimuli at 9 months, 2 years and 5 years using a Tobii© x60 eye-tracker and bespoke analysis software (Matlab). Images are presented on a display monitor with a resolution of 1,440 × 900 pixels. The Tobii© ×60 system tracks both eyes to a rated accuracy of 0.3 degrees at a rate of 60 Hz. We analyse looking patterns, including time to first fixate and looking time at areas of interest, in tasks designed to enable inference about social development, attention, and processing speed[35 39].

Standardised assessments

Standardised assessments of neurodevelopment by direct observation at appropriate time points are: Bayley-III scales; Mullen Scales of Early Learning (MSEL); parental IQ (National Adult Reading Test). We selected the MSEL for assessing cognitive ability at 5 years because: it has separate verbal and nonverbal standardised scores so is useful for assessing cognitive abilities in children with social communication and language difficulties; internal consistency reliability and test/retest reliability for the 5 component scales is high; and the early learning composite (and its components) correlate with other psychometric tests used in this age group. We will use validated questionnaires to assess: infant/parent temperament; parent/family characteristics (postnatal depression, stress, quality of life, socioeconomic status); infant / child sleep habits; language development; social development; executive functions; cerebral visual impairment; medical diagnoses; and behavioural outcomes (parent and teacher ratings). We also record parent-child interaction for subsequent analysis via video coding of complex behaviours in a naturalistic context.

Susceptibility to viral infection

We collect unstimulated nasal secretion samples (nasosorption samples) using methods described by Thwaites et al[40]. This collection is brief, minimally invasive and a minimally

For peer review only

distressing process. Nasosorption Nasal lining fluid is collected using Nasosorption Fxi synthetic absorption matrix strips inserted into the anterior part of the inferior turbinate of the nasal cavity. After 30 seconds of absorption, the strip is removed, capped, maintained at 4°C for up to 4 hours and then frozen at −80°C. From these nasal fluid samples we will assess the levels of antimicrobial peptides, including cathelicidin, and inflammatory cytokines, by ELISA or luminex assay. Collection of these at birth (term equivalent age), 9 months and 2 years will enable us to characterise birth levels, levels at timepoints significant for respiratory syncytial virus (RSV) infection/disease and at a later time point.

Respiratory and gut microbiota

We collect faecal and nasopharyngeal swabs (paediatric Copan e-swab with flocked nylon fiber tip) as has been described in the WHO-guideline for respiratory sampling of bacterial pathogens[41]. Fecal material and e-swabs (in RNA protect), are frozen at -80°C until further analyses. DNA and RNA will be extracted[42] and metagenomics analyses will be executed by 16S-based sequencing according to previously described methods[43]. We will study temporal relationships between preterm birth and early life characteristics, consecutive microbiota development, inflammation and methylation findings, and respiratory and neurocognitive developmental outcomes.

Computational Motor Assessment

Light-weight, wearable, wireless motion sensors are deployed to record the movement of a sub-set of infants at 9 months during the Still-Face paradigm and Parent-Child interaction. Data are anonymised before being securely transferred to the University of Strathclyde for analysis. These data will be analysed to test for differences in motor function between at-risk and low-risk infants, and will employ machine learning algorithms to detect patterns predictive of developmental outcome at 2 and 5 years, and their potential for clinical stratification across the neurodevelopmental disorders and psychometric profiles (IQ, adaptive function, language). Further, motor data at 9 months can be correlated against neuroanatomical features measured by MRI scan at birth and developmental scales at 9 months.

Patient and Public Involvement

We seek feedback from parents / carers to monitor satisfaction with research participation at 9 months, 2 years and 5 years, and we have a public facing website that describes results from the study.

For peer review only

ETHICS AND DISSEMINATION

Safety assessment

There are no safety issues associated with collection of: placental tissue, umbilical cord / neonatal blood, saliva, faeces or hair. There are no safety issues in the conduct of planned neuropsychological assessments.

MRI does not involve ionizing radiation and there are no known risks from MRI provided standard safety measures for 3T scanning are in place. Infants are fed and wrapped and allowed to sleep naturally in the scanner. Pulse oximetry, electrocardiography and temperature are monitored. Flexible earplugs and neonatal earmuffs (MiniMuffs, Natus) are used for acoustic protection. All scans are supervised by a doctor or nurse trained in neonatal resuscitation. The scan is interrupted if there are any abnormalities in monitoring or if the baby wakes.

It is possible that incidental findings may be found on MRI or from questionnaires, for example intracranial structural anomalies or postnatal depression, respectively. In these circumstances, the findings are discussed with the participant's parent, and referral to the appropriate NHS service is made.

Ethical approvals

The study has been approved by the National Research Ethics Service (South East Scotland Research Ethics Committee), NRES numbers 11/55/0061 and 13/SS/0143 (Phase 1) and NRES number 16/SS/0154 (Phase 2); and by NHS Lothian Research & Development (2016/0255).

Governance

The study is run by a management group that includes the principal investigator, a minimum of two co-investigators, the study coordinator and administrative and financial officers. A delegation log details the responsibilities of each member of staff working on the study. A scientific advisory board oversees the conduct and progress of the study. The study is co-sponsored by the University of Edinburgh & NHS Lothian Academic and Clinical Central Office for Research and Development (ACCORD).

Publication and data statement

The principles set down by the International Committee of Medical Journal Editors for authorship and non-author contributors are followed for publications and presentations resulting from the study. A Data Access and Collaboration Policy sets out the terms and

For peer review only

conditions on which deidentified TEBC data, stimuli and tasks are accessible to the research community following reasonable request (www.tebc.ed.ac.uk).

Author contributions

JPB designed the study with input from all the authors. JPB, JH, MJT, RMR, SC, JS, DB, DJD, AJD, MEB and SF-W contributed to the establishment and refinement of study procedures and critically revised the manuscript. All authors approved the final version of the manuscript.

Competing interests

None declared.

Acknowledgements

The authors would like to thank participating families, and NHS colleagues at the Simpson Centre for Reproductive Health who support this study. We would like to thank Mrs Bavanthe Navarathne, a parent representative on the scientific advisory board, and other past and present members of the scientific advisory board (Frances Cowan, Chiara Nosarti, David Porteous, Hugh Rabagliati, Joanna Wardlaw, Heather Whalley). We are grateful to the following collaborators, colleagues and students who support the study: Gayle Barclay, Justyna Binkowska, Gillian Black, Manuel Blesa, Nis Borbye-Lorenzen, Geoff Carlson, Yu Wei Chua, Simon Cox, Hilary Cruikshank, Bethan Dean, Jonathan Delafield-Butt, Fiona Denison, Margaret Evans, Paola Galdi, Peter Ghazal, Lorna Ginnell, Charlotte Jardine, Gillian Lamb, Victoria Ledsham, Riccardo Marioni, Andrew McIntosh, Barbara Nugent, Lee Murphy, Sinéad O'Carroll, Alan Quigley, Alan Mulvihill, Magda Rudnicka, Scott Semple, Kristin Skögstrand, Sarah Stock, David Stoye, Gemma Sullivan, Kadi Vaher, colleagues at the Genetics Core of the Edinburgh Clinical Research Facility, and radiographers at the Edinburgh Imaging Facility Royal Infirmary of Edinburgh.

Funding statement

The TEBC study is funded by the charity Theirworld (www.theirworld.org) and is carried out in the University of Edinburgh MRC Centre for Reproductive Health (MRC G1002033). Susceptibility to viral infection studies are supported by grants from Action Medical Research (GN2703) and Chief Scientist Office (TCS/18/02). Respiratory microbiota studies are supported by grants from the Chief Scientist Office (SCAF/16/03), and DNA methylation and gut microbiota studies are supported by the Wellcome Trust (203769/Z/16/A and 220043/Z/19/Z). The MRI facility is funded by Wellcome Trust 104916/Z/14/Z, Dunhill Trust

For peer review only

R380R/1114, Edinburgh and Lothians Health Foundation 2012/17, Muir Maxwell Research Fund, and Edinburgh Imaging, University of Edinburgh. MJT was supported by NHS Lothian Research and Development Office, and RMR and AJD receive support from the British Heart Foundation (RE/18/5/34216).

For peer review only

REFERENCES

1. Chawanpaiboon S, Vogel JP, Moller AB, et al. Global, regional, and national estimates of levels of preterm birth in 2014: a systematic review and modelling analysis. *Lancet Glob Health* 2019;7(1):e37-e46. doi: 10.1016/s2214-109x(18)30451-0 [published Online First: 2018/11/06]
2. Mehler K, Oberthuer A, Keller T, et al. Survival Among Infants Born at 22 or 23 Weeks' Gestation Following Active Prenatal and Postnatal Care. *JAMA Pediatr* 2016;170(7):671-7. doi: 10.1001/jamapediatrics.2016.0207 [published Online First: 2016/05/24]
3. Patel RM, Rysavy MA, Bell EF, et al. Survival of Infants Born at Periviable Gestational Ages. *Clin Perinatol* 2017;44(2):287-303. doi: 10.1016/j.clp.2017.01.009 [published Online First: 2017/05/10]
4. Norman M, Hallberg B, Abrahamsson T, et al. Association Between Year of Birth and 1-Year Survival Among Extremely Preterm Infants in Sweden During 2004-2007 and 2014-2016. *Jama* 2019;321(12):1188-99. doi: 10.1001/jama.2019.2021 [published Online First: 2019/03/27]
5. Myrhaug HT, Brurberg KG, Hov L, et al. Survival and Impairment of Extremely Premature Infants: A Meta-analysis. *Pediatrics* 2019;143(2) doi: 10.1542/peds.2018-0933 [published Online First: 2019/02/02]
6. Johnson S, Marlow N. Early and long-term outcome of infants born extremely preterm. *Arch Dis Child* 2017;102(1):97-102. doi: 10.1136/archdischild-2015-309581 [published Online First: 2016/08/12]
7. Mangham LJ, Petrou S, Doyle LW, et al. The cost of preterm birth throughout childhood in England and Wales. *Pediatrics* 2009;123(2):e312-e27.
8. Porter EJ, Counsell SJ, Edwards AD, et al. Tract-based spatial statistics of magnetic resonance images to assess disease and treatment effects in perinatal asphyxial encephalopathy. *PediatrRes* 2010;68(3):205-09.
9. Ball G, Boardman JP, Arichi T, et al. Testing the sensitivity of tract-based spatial statistics to simulated treatment effects in preterm neonates. *PLoSOne* 2013;8(7):e67706.
10. Monnelly VJ, Anblagan D, Quigley A, et al. Prenatal methadone exposure is associated with altered neonatal brain development. *Neuroimage Clin* 2018;18:9-14. doi: 10.1016/j.nicl.2017.12.033 [published Online First: 2018/01/13]
11. Blesa M, Sullivan G, Anblagan D, et al. Early breast milk exposure modifies brain connectivity in preterm infants. *Neuroimage* 2019;184:431-39. doi: 10.1016/j.neuroimage.2018.09.045 [published Online First: 2018/09/22]
12. Anblagan D, Pataky R, Evans MJ, et al. Association between preterm brain injury and exposure to chorioamnionitis during fetal life. *Sci Rep* 2016;6:37932. doi: 10.1038/srep37932 [published Online First: 2016/12/03]
13. Ball G, Counsell SJ, Anjari M, et al. An optimised tract-based spatial statistics protocol for neonates: applications to prematurity and chronic lung disease. *Neuroimage* 2010;53(1):94-102.
14. Jakab A, Ruegger C, Bucher HU, et al. Network based statistics reveals trophic and neuroprotective effect of early high dose erythropoetin on brain connectivity in very preterm infants. *NeuroImage Clinical* 2019;22:101806. doi: 10.1016/j.nicl.2019.101806 [published Online First: 2019/04/18]
15. Boardman JP, Counsell SJ, Rueckert D, et al. Early growth in brain volume is preserved in the majority of preterm infants. *AnnNeurol* 2007;62(2):185-92.

16. Inder TE, Warfield SK, Wang H, et al. Abnormal cerebral structure is present at term in premature infants. *Pediatrics* 2005;115(2):286-94.
17. Boardman JP, Counsell SJ. Invited Review: Factors associated with atypical brain development in preterm infants: insights from magnetic resonance imaging. *Neuropathol Appl Neurobiol* 2019 doi: 10.1111/nan.12589 [published Online First: 2019/11/21]
18. Ball G, Aljabar P, Nongena P, et al. Multimodal image analysis of clinical influences on preterm brain development. *Ann Neurol* 2017;82(2):233-46. doi: 10.1002/ana.24995 [published Online First: 2017/07/19]
19. Serag A, Wilkinson AG, Telford EJ, et al. SEGMA: An Automatic SEGMentation Approach for Human Brain MRI Using Sliding Window and Random Forests. *Front Neuroinform* 2017;11:2. doi: 10.3389/fninf.2017.00002 [published Online First: 2017/02/07]
20. Blesa M, Serag A, Wilkinson AG, et al. Parcellation of the Healthy Neonatal Brain into 107 Regions Using Atlas Propagation through Intermediate Time Points in Childhood. *Front Neurosci* 2016;10:220. doi: 10.3389/fnins.2016.00220 [published Online First: 2016/06/01]
21. Serag A, Blesa M, Moore EJ, et al. Accurate Learning with Few Atlases (ALFA): an algorithm for MRI neonatal brain extraction and comparison with 11 publicly available methods. *Sci Rep* 2016;6:23470. doi: 10.1038/srep23470 [published Online First: 2016/03/25]
22. Pecheva D, Tournier JD, Pietsch M, et al. Fixel-based analysis of the preterm brain: Disentangling bundle-specific white matter microstructural and macrostructural changes in relation to clinical risk factors. *NeuroImage Clinical* 2019;23:101820. doi: 10.1016/j.nicl.2019.101820 [published Online First: 2019/04/17]
23. Dickie DA, Shenkin SD, Anblagan D, et al. Whole Brain Magnetic Resonance Image Atlases: A Systematic Review of Existing Atlases and Caveats for Use in Population Imaging. *Frontiers in neuroinformatics* 2017;11:1. doi: 10.3389/fninf.2017.00001 [published Online First: 2017/02/06]
24. Job DE, Dickie DA, Rodriguez D, et al. A brain imaging repository of normal structural MRI across the life course: Brain Images of Normal Subjects (BRAINS). *Neuroimage* 2016 doi: 10.1016/j.neuroimage.2016.01.027 [published Online First: 2016/01/23]
25. Shenkin SD, Pernet C, Nichols TE, et al. Improving data availability for brain image biobanking in healthy subjects: Practice-based suggestions from an international multidisciplinary working group. *NeuroImage* 2017;153:399-409. doi: 10.1016/j.neuroimage.2017.02.030 [published Online First: 2017/02/25]
26. Redline RW, Faye-Petersen O, Heller D, et al. Amniotic infection syndrome: nosology and reproducibility of placental reaction patterns. *Pediatr Dev Pathol* 2003;6(5):435-48. doi: 10.1007/s10024-003-7070-y [published Online First: 2004/01/08]
27. Skogstrand K, Thorsen P, Norgaard-Pedersen B, et al. Simultaneous measurement of 25 inflammatory markers and neurotrophins in neonatal dried blood spots by immunoassay with xMAP technology. *Clin Chem* 2005;51(10):1854-66. doi: 10.1373/clinchem.2005.052241 [published Online First: 2005/08/06]
28. Caruyer E, Lenglet C, Sapiro G, et al. Design of multishell sampling schemes with uniform coverage in diffusion MRI. *Magn Reson Med* 2013;69(6):1534-40. doi: 10.1002/mrm.24736 [published Online First: 2013/04/30]
29. Woodward LJ, Anderson PJ, Austin NC, et al. Neonatal MRI to predict neurodevelopmental outcomes in preterm infants. *NEnglJ Med* 2006;355(7):685-94.

30. Leuchter RH, Gui L, Poncet A, et al. Association between early administration of high-dose erythropoietin in preterm infants and brain MRI abnormality at term-equivalent age. *Jama* 2014;312(8):817-24. doi: 10.1001/jama.2014.9645 [published Online First: 2014/08/27]
31. Anblagan D, Bastin ME, Sparrow S, et al. Tract shape modeling detects changes associated with preterm birth and neuroprotective treatment effects. *NeuroImage: Clinical* 2015;8:51-58. doi: doi:10.1016/j.nicl.2015.03.021
32. Smith SM, Jenkinson M, Johansen-Berg H, et al. Tract-based spatial statistics: voxelwise analysis of multi-subject diffusion data. *Neuroimage* 2006;31(4):1487-505.
33. Makropoulos A, Robinson EC, Schuh A, et al. The developing human connectome project: A minimal processing pipeline for neonatal cortical surface reconstruction. *Neuroimage* 2018;173:88-112. doi: 10.1016/j.neuroimage.2018.01.054 [published Online First: 2018/02/08]
34. Boardman JP, Walley A, Ball G, et al. Common genetic variants and risk of brain injury after preterm birth. *Pediatrics* 2014;133(6):e1655-63. doi: 10.1542/peds.2013-3011 [published Online First: 2014/05/14]
35. Telford EJ, Fletcher-Watson S, Gillespie-Smith K, et al. Preterm birth is associated with atypical social orienting in infancy detected using eye tracking. *J Child Psychol Psychiatry* 2016;57(7):861-8. doi: 10.1111/jcpp.12546 [published Online First: 2016/03/05]
36. Sparrow S, Manning JR, Cartier J, et al. Epigenomic profiling of preterm infants reveals DNA methylation differences at sites associated with neural function. *Transl Psychiatry* 2016;6:e716. doi: 10.1038/tp.2015.210 [published Online First: 2016/01/20]
37. Krishnan ML, Van Steenwinckel J, Schang AL, et al. Integrative genomics of microglia implicates DLG4 (PSD95) in the white matter development of preterm infants. *Nat Commun* 2017;8(1):428. doi: 10.1038/s41467-017-00422-w [published Online First: 2017/09/07]
38. Telford EJ, Cox SR, Fletcher-Watson S, et al. A latent measure explains substantial variance in white matter microstructure across the newborn human brain. *Brain Struct Funct* 2017;222(9):4023-33. doi: 10.1007/s00429-017-1455-6 [published Online First: 2017/06/08]
39. Gillespie-Smith K, Boardman JP, Murray IC, et al. Multiple Measures of Fixation on Social Content in Infancy: Evidence for a Single Social Cognitive Construct? *Infancy* 2016;21(2):241-57. doi: 10.1111/infa.12103 [published Online First: 2016/03/08]
40. Thwaites RS, Jarvis HC, Singh N, et al. Absorption of Nasal and Bronchial Fluids: Precision Sampling of the Human Respiratory Mucosa and Laboratory Processing of Samples. *Journal of visualized experiments : JoVE* 2018(131) doi: 10.3791/56413 [published Online First: 2018/02/15]
41. Satzke C, Turner P, Virolainen-Julkunen A, et al. Standard method for detecting upper respiratory carriage of Streptococcus pneumoniae: updated recommendations from the World Health Organization Pneumococcal Carriage Working Group. *Vaccine* 2013;32(1):165-79. doi: 10.1016/j.vaccine.2013.08.062 [published Online First: 2013/12/18]
42. de Steenhuijsen Piters WA, Heinonen S, Hasrat R, et al. Nasopharyngeal Microbiota, Host Transcriptome, and Disease Severity in Children with Respiratory Syncytial Virus Infection. *American journal of respiratory and critical care medicine*

For peer review only

2016;194(9):1104-15. doi: 10.1164/rccm.201602-0220OC [published Online First: 2016/11/01]
43. Bosch A, de Steenhuijsen Piters WAA, van Houten MA, et al. Maturation of the Infant Respiratory Microbiota, Environmental Drivers, and Health Consequences. A Prospective Cohort Study. *American journal of respiratory and critical care medicine* 2017;196(12):1582-90. doi: 10.1164/rccm.201703-0554OC [published Online First: 2017/07/01]

For peer review only

SIEMENS MAGNETOM Prisma

Table of contents

\\Study Protocols
BRAIN
Neonates
Theirworld - E161723
localizer_neonate
t2_haste_localiser
t2_blade_v3
t2_space_sag_p4_iso_v2x
DTI_Neonate_v6b_dummy
DTI_Neonate_v6b_rev
DTI_Neonate_v6b_pt1
DTI_Neonate_v6b_pt2
MPRAGE-v4
SWI_v2
t2_blade_dark-fluid_tra_v3

For peer review only

SIEMENS MAGNETOM Prisma

\\Study Protocols\BRAIN\Neonates\Theirworld - E161723\localizer_neonate
TA: 0:12 PM: REF Voxel size: 0.5×0.5×7.0 mmPAT: Off Rel. SNR: 1.00 : fl

Properties

Prio recon	On
Load images to viewer	On
Inline movie	Off
Auto store images	On
Load images to stamp segments	On
Load images to graphic segments	On
Auto open inline display	Off
Auto close inline display	Off
Start measurement without further preparation	Off
Wait for user to start	Off
Start measurements	Single measurement

Routine

Slice group	1
Slices	1
Dist. factor	20 %
Position	L0.0 P47.8 F62.3 mm
Orientation	Sagittal
Phase enc. dir.	A >> P
Slice group	2
Slices	1
Dist. factor	20 %
Position	L0.0 P47.8 F62.3 mm
Orientation	Transversal
Phase enc. dir.	A >> P
Slice group	3
Slices	1
Dist. factor	20 %
Position	L0.0 P47.8 F62.3 mm
Orientation	Coronal
Phase enc. dir.	R >> L
AutoAlign	---
Phase oversampling	0 %
FoV read	250 mm
FoV phase	100.0 %
Slice thickness	7.0 mm
TR	7.5 ms
TE	3.69 ms
Averages	2
Concatenations	3
Filter	Prescan Normalize, Elliptical filter
Coil elements	PeH;PeN

Contrast - Common

TR	7.5 ms
TE	3.69 ms
TD	0 ms
MTC	Off
Magn. preparation	None
Flip angle	20 deg
Fat suppr.	None
Water suppr.	None
SWI	Off

Contrast - Dynamic

Averages	2
Averaging mode	Short term
Reconstruction	Magnitude
Measurements	1

Contrast - Dynamic

Multiple series	Each measurement

Resolution - Common

FoV read	250 mm
FoV phase	100.0 %
Slice thickness	7.0 mm
Base resolution	256
Phase resolution	91 %
Phase partial Fourier	Off
Interpolation	On

Resolution - iPAT

PAT mode	None

Resolution - Filter Image

Image Filter	Off
Distortion Corr.	Off
Prescan Normalize	On
Unfiltered images	Off
Normalize	Off
B1 filter	Off

Resolution - Filter Rawdata

Raw filter	Off
Elliptical filter	On

Geometry - Common

Slice group	1
Slices	1
Dist. factor	20 %
Position	L0.0 P47.8 F62.3 mm
Orientation	Sagittal
Phase enc. dir.	A >> P
Slice group	2
Slices	1
Dist. factor	20 %
Position	L0.0 P47.8 F62.3 mm
Orientation	Transversal
Phase enc. dir.	A >> P
Slice group	3
Slices	1
Dist. factor	20 %
Position	L0.0 P47.8 F62.3 mm
Orientation	Coronal
Phase enc. dir.	R >> L
FoV read	250 mm
FoV phase	100.0 %
Slice thickness	7.0 mm
TR	7.5 ms
Multi-slice mode	Sequential
Series	Interleaved
Concatenations	3

Geometry - AutoAlign

Slice group	1
Position	L0.0 P47.8 F62.3 mm
Orientation	Sagittal
Phase enc. dir.	A >> P
Slice group	2
Position	L0.0 P47.8 F62.3 mm

For peer review only

For peer review only - http://bmjopen.bmj.com/site/about/guidelines.xhtml

SIEMENS MAGNETOM Prisma

Geometry - AutoAlign

Orientation	Transversal
Phase enc. dir.	A >> P
Slice group	3
Position	L0.0 P47.8 F62.3 mm
Orientation	Coronal
Phase enc. dir.	R >> L
AutoAlign	---
Initial Position	L0.0 P47.8 F62.3
L	0.0 mm
P	47.8 mm
F	62.3 mm
Initial Rotation	0.00 deg
Initial Orientation	Sagittal

Geometry - Saturation

Saturation mode	Standard
Fat suppr.	None
Water suppr.	None
Special sat.	None

Geometry - Tim Planning Suite

Set-n-Go Protocol	Off
Table position	H
Table position	0 mm
Inline Composing	Off

System - Miscellaneous

Positioning mode	REF
Table position	H
Table position	0 mm
MSMA	S - C - T
Sagittal	R >> L
Coronal	A >> P
Transversal	F >> H
Coil Combine Mode	Adaptive Combine
Save uncombined	Off
Matrix Optimization	Off
AutoAlign	---
Coil Select Mode	Default

System - Adjustments

B0 Shim mode	Tune up
B1 Shim mode	TrueForm
Adjust with body coil	Off
Confirm freq. adjustment	Off
Assume Dominant Fat	Off
Assume Silicone	Off
Adjustment Tolerance	Auto

System - Adjust Volume

Position	Isocenter
Orientation	Transversal
Rotation	0.00 deg
A >> P	263 mm
R >> L	350 mm
F >> H	350 mm
Reset	Off

System - pTx Volumes

B1 Shim mode	TrueForm
Excitation	Slice-sel.

System - Tx/Rx

Frequency 1H	123.244318 MHz
Correction factor	1
Gain	High
Img. Scale Cor.	1.000
Reset	Off
? Ref. amplitude 1H	0.000 V

Physio - Signal1

1st Signal/Mode	None
TR	7.5 ms
Concatenations	3
Segments	1

Physio - Cardiac

Tagging	None
Magn. preparation	None
Fat suppr.	None
Dark blood	Off
FoV read	250 mm
FoV phase	100.0 %
Phase resolution	91 %

Physio - PACE

Resp. control	Off
Concatenations	3

Inline - Common

Subtract	Off
Measurements	1
StdDev	Off
Liver registration	Off
Save original images	On

Inline - MIP

MIP-Sag	Off
MIP-Cor	Off
MIP-Tra	Off
MIP-Time	Off
Save original images	On

Inline - Soft Tissue

Wash - In	Off
Wash - Out	Off
TTP	Off
PEI	Off
MIP - time	Off
Measurements	1

Inline - Composing

Inline Composing	Off
Distortion Corr.	Off

Inline - MapIt

Save original images	On
MapIt	None
Flip angle	20 deg
Measurements	1
Contrasts	1
TR	7.5 ms
TE	3.69 ms

Sequence - Part 1

Introduction	On

For peer review only - http://bmjopen.bmj.com/site/about/guidelines.xhtml

SIEMENS MAGNETOM Prisma

Sequence - Part 1

Dimension	2D
Phase stabilisation	Off
Asymmetric echo	Allowed
Contrasts	1
Flow comp.	No
Multi-slice mode	Sequential
Bandwidth	320 Hz/Px

Sequence - Part 2

Segments	1
Acoustic noise reduction	None
RF pulse type	Fast
Gradient mode	Fast
Excitation	Slice-sel.
RF spoiling	On

Sequence - Assistant

Mode	Off
Allowed delay	0 s

For peer review only

SIEMENS MAGNETOM Prisma

\\Study Protocols\BRAIN\Neonates\Theirworld - E161723\t2_haste_localiser
TA: 6.0 s PM: REF Voxel size: 0.7×0.7×4.0 mmPAT: 2 Rel. SNR: 1.00 : h

Properties

Prio recon	Off
Load images to viewer	On
Inline movie	Off
Auto store images	On
Load images to stamp segments	On
Load images to graphic segments	On
Auto open inline display	Off
Auto close inline display	Off
Start measurement without further preparation	Off
Wait for user to start	Off
Start measurements	Single measurement

Routine

Slice group	1
Slices	1
Dist. factor	30 %
Position	Isocenter
Orientation	Sagittal
Phase enc. dir.	A >> P
Slice group	2
Slices	1
Dist. factor	30 %
Position	L0.0 P0.0 H5.2 mm
Orientation	Transversal
Phase enc. dir.	R >> L
Slice group	3
Slices	1
Dist. factor	30 %
Position	L0.0 P0.0 H10.4 mm
Orientation	Coronal
Phase enc. dir.	R >> L
AutoAlign	---
Phase oversampling	0 %
FoV read	220 mm
FoV phase	100.0 %
Slice thickness	4.0 mm
TR	1500.0 ms
TE	94 ms
Averages	1
Concatenations	1
Filter	Prescan Normalize, Elliptical filter
Coil elements	HE1-4

Contrast - Common

TR	1500.0 ms
TE	94 ms
MTC	Off
Magn. preparation	None
Flip angle	150 deg
Fat suppr.	None
Water suppr.	None
Restore magn.	Off

Contrast - Dynamic

Averages	1
Averaging mode	Long term
Reconstruction	Magnitude
Measurements	1
Multiple series	Each measurement

Resolution - Common

FoV read	220 mm
FoV phase	100.0 %
Slice thickness	4.0 mm
Base resolution	320
Phase resolution	80 %
Phase partial Fourier	4/8
Interpolation	Off

Resolution - iPAT

PAT mode	GRAPPA
Accel. factor PE	2
Ref. lines PE	24
Reference scan mode	Integrated

Resolution - Filter Image

Image Filter	Off
Distortion Corr.	Off
Prescan Normalize	On
Unfiltered images	Off
Normalize	Off
B1 filter	Off

Resolution - Filter Rawdata

Raw filter	Off
Elliptical filter	On

Geometry - Common

Slice group	1
Slices	1
Dist. factor	30 %
Position	Isocenter
Orientation	Sagittal
Phase enc. dir.	A >> P
Slice group	2
Slices	1
Dist. factor	30 %
Position	L0.0 P0.0 H5.2 mm
Orientation	Transversal
Phase enc. dir.	R >> L
Slice group	3
Slices	1
Dist. factor	30 %
Position	L0.0 P0.0 H10.4 mm
Orientation	Coronal
Phase enc. dir.	R >> L
FoV read	220 mm
FoV phase	100.0 %
Slice thickness	4.0 mm
TR	1500.0 ms
Multi-slice mode	Single shot
Series	Interleaved
Concatenations	1

Geometry - AutoAlign

Slice group	1
Position	Isocenter
Orientation	Sagittal
Phase enc. dir.	A >> P
Slice group	2
Position	L0.0 P0.0 H5.2 mm

For peer review only

SIEMENS MAGNETOM Prisma

Geometry - AutoAlign

Orientation	Transversal
Phase enc. dir.	R >> L
Slice group	3
Position	L0.0 P0.0 H10.4 mm
Orientation	Coronal
Phase enc. dir.	R >> L
AutoAlign	---
Initial Position	Isocenter
L	0.0 mm
P	0.0 mm
H	0.0 mm
Initial Rotation	0.00 deg
Initial Orientation	Sagittal

Geometry - Saturation

Fat suppr.	None
Water suppr.	None
Restore magn.	Off
Special sat.	None

Geometry - Navigator

Geometry - Tim Planning Suite

Set-n-Go Protocol	Off
Table position	H
Table position	0 mm
Inline Composing	Off

System - Miscellaneous

Positioning mode	REF
Table position	H
Table position	0 mm
MSMA	S - C - T
Sagittal	R >> L
Coronal	A >> P
Transversal	F >> H
Coil Combine Mode	Adaptive Combine
Save uncombined	Off
Matrix Optimization	Off
AutoAlign	---
Coil Select Mode	On - AutoCoilSelect

System - Adjustments

B0 Shim mode	Tune up
B1 Shim mode	TrueForm
Adjust with body coil	Off
Confirm freq. adjustment	Off
Assume Dominant Fat	Off
Assume Silicone	Off
Adjustment Tolerance	Auto

System - Adjust Volume

Position	Isocenter
Orientation	Transversal
Rotation	0.00 deg
A >> P	263 mm
R >> L	350 mm
F >> H	350 mm
Reset	Off

System - pTx Volumes

B1 Shim mode	TrueForm

System - Tx/Rx

Frequency 1H	123.244318 MHz
Correction factor	1
Gain	High
Img. Scale Cor.	1.000
Reset	Off
? Ref. amplitude 1H	0.000 V

Physio - Signal1

1st Signal/Mode	None
TR	1500.0 ms
Concatenations	1

Physio - Cardiac

Magn. preparation	None
Fat suppr.	None
Dark blood	Off
FoV read	220 mm
FoV phase	100.0 %
Phase resolution	80 %

Physio - PACE

Resp. control	Off
Concatenations	1

Inline - Common

Subtract	Off
Measurements	1
StdDev	Off
Save original images	On

Inline - MIP

MIP-Sag	Off
MIP-Cor	Off
MIP-Tra	Off
MIP-Time	Off
Save original images	On

Inline - Composing

Inline Composing	Off
Distortion Corr.	Off

Sequence - Part 1

Introduction	On
Dimension	2D
Contrasts	1
Flow comp.	No
Multi-slice mode	Single shot
Echo spacing	7.22 ms
Bandwidth	601 Hz/Px

Sequence - Part 2

RF pulse type	Normal
Gradient mode	Whisper
Hyperecho	Off
Turbo factor	256

Sequence - Assistant

Mode	Min flip angle
Min flip angle	130 deg
Allowed delay	60 s

For peer review only

SIEMENS MAGNETOM Prisma

\\Study Protocols\BRAIN\Neonates\Theirworld - E161723\t2_blade_v3
TA: 2:29 PM: REF Voxel size: 0.7×0.7×3.0 mmPAT: 2 Rel. SNR: 1.00 : qtseBR_rr

Properties

Prio recon	Off
Load images to viewer	On
Inline movie	Off
Auto store images	On
Load images to stamp segments	On
Load images to graphic segments	Off
Auto open inline display	Off
Auto close inline display	Off
Start measurement without further preparation	Off
Wait for user to start	Off
Start measurements	Single measurement

Routine

Slice group	1
Slices	40
Dist. factor	0 %
Position	R1.2 P40.0 H50.2 mm
Orientation	Transversal
Phase enc. dir.	A >> P
AutoAlign	---
Phase oversampling	0.0 %
FoV read	220 mm
FoV phase	100.0 %
Slice thickness	3.0 mm
TR	4100.0 ms
TE	207 ms
Averages	1
Concatenations	4
Filter	Prescan Normalize
Coil elements	PeH;PeN

Contrast - Common

TR	4100.0 ms
TE	207 ms
TD	0.0 ms
MTC	Off
Magn. preparation	None
Flip angle	90 deg
Fat suppr.	None
Water suppr.	None
Restore magn.	On

Contrast - Dynamic

Averages	1
Averaging mode	Short term
Reconstruction	Magnitude
Measurements	1
Multiple series	Each measurement

Resolution - Common

FoV read	220 mm
FoV phase	100.0 %
Slice thickness	3.0 mm
Base resolution	320
BLADE coverage	100.0 %
Trajectory	BLADE
Interpolation	Off

Resolution - iPAT

PAT mode	GRAPPA
Accel. factor PE	2
Ref. lines PE	8
Reference scan mode	Integrated

Resolution - Filter Image

Image Filter	Off
Distortion Corr.	Off
Prescan Normalize	On
Unfiltered images	Off
Normalize	Off
B1 filter	Off

Resolution - Filter Rawdata

Raw filter	Off
Elliptical filter	Off

Geometry - Common

Slice group	1
Slices	40
Dist. factor	0 %
Position	R1.2 P40.0 H50.2 mm
Orientation	Transversal
Phase enc. dir.	A >> P
FoV read	220 mm
FoV phase	100.0 %
Slice thickness	3.0 mm
TR	4100.0 ms
Multi-slice mode	Interleaved
Series	Interleaved
Concatenations	4

Geometry - AutoAlign

Slice group	1
Position	R1.2 P40.0 H50.2 mm
Orientation	Transversal
Phase enc. dir.	A >> P
AutoAlign	---
Initial Position	R1.2 P40.0 H50.2
R	1.2 mm
P	40.0 mm
H	50.2 mm
Initial Rotation	0.00 deg
Initial Orientation	Transversal

Geometry - Saturation

Fat suppr.	None
Water suppr.	None
Restore magn.	On
Special sat.	None

Geometry - Navigator

Geometry - Tim Planning Suite

Set-n-Go Protocol	Off
Table position	H
Table position	0 mm
Inline Composing	Off

For peer review only

SIEMENS MAGNETOM Prisma

System - Miscellaneous

Positioning mode	REF
Table position	H
Table position	0 mm
MSMA	S - C - T
Sagittal	R >> L
Coronal	A >> P
Transversal	F >> H
Coil Combine Mode	Adaptive Combine
Save uncombined	Off
Matrix Optimization	Off
AutoAlign	---
Coil Select Mode	On - AutoCoilSelect

System - Adjustments

B0 Shim mode	Tune up
B1 Shim mode	TrueForm
Adjust with body coil	Off
Confirm freq. adjustment	Off
Assume Dominant Fat	Off
Assume Silicone	Off
Adjustment Tolerance	Auto

System - Adjust Volume

Position	Isocenter
Orientation	Transversal
Rotation	0.00 deg
A >> P	263 mm
R >> L	350 mm
F >> H	350 mm
Reset	Off

System - pTx Volumes

B1 Shim mode	TrueForm

System - Tx/Rx

Frequency 1H	123.244318 MHz
Correction factor	1
Gain	High
Img. Scale Cor.	1.000
Reset	Off
? Ref. amplitude 1H	0.000 V

Physio - Signal1

1st Signal/Mode	None
TR	4100.0 ms
Concatenations	4

Physio - Cardiac

Magn. preparation	None
Fat suppr.	None
Dark blood	Off
FoV read	220 mm
FoV phase	100.0 %
BLADE coverage	100.0 %
Trajectory	BLADE

Physio - PACE

Resp. control	Off
Concatenations	4

Inline - Common

Subtract	Off
Measurements	1
StdDev	Off
Save original images	On

Inline - MIP

MIP-Sag	Off
MIP-Cor	Off
MIP-Tra	Off
MIP-Time	Off
Save original images	On

Inline - Composing

Inline Composing	Off
Distortion Corr.	Off

Sequence - Part 1

Introduction	On
Dimension	2D
Compensate T2 decay	Off
Contrasts	1
Flow comp.	Read
Multi-slice mode	Interleaved
Free echo spacing	Off
Echo spacing	10.9 ms
Bandwidth	363 Hz/Px

Sequence - Part 2

Define	Turbo factor
Echo trains per slice	8
Phase correction	Automatic
Acoustic noise reduction	Active
RF pulse type	Low SAR
Gradient mode	Fast
Hyperecho	On
WARP	Off
Motion correction	On
Red. EC sensitivity	Off
Turbo factor	36

Sequence - Assistant

Mode	Off
Allowed delay	30 s

For peer review only

SIEMENS MAGNETOM Prisma

\\Study Protocols\BRAIN\Neonates\Theirworld - E161723\t2_space_sag_p4_iso_v2x

TA: 2:13 PM: REF Voxel size: 1.0×1.0×1.0 mmPAT: 4 Rel. SNR: 1.00 : spcR

Properties

Prio recon	Off
Load images to viewer	On
Inline movie	Off
Auto store images	On
Load images to stamp segments	Off
Load images to graphic segments	Off
Auto open inline display	Off
Auto close inline display	Off
Start measurement without further preparation	Off
Wait for user to start	Off
Start measurements	Single measurement

Routine

Slab group	1
Slabs	1
Position	R1.2 P36.9 H0.0 mm
Orientation	Sagittal
Phase enc. dir.	A >> P
AutoAlign	---
Phase oversampling	0 %
Slice oversampling	0.0 %
Slices per slab	160
FoV read	128 mm
FoV phase	150.0 %
Slice thickness	1.00 mm
TR	3200 ms
TE	409 ms
Averages	1.4
Concatenations	1
Filter	Raw filter, Prescan Normalize
Coil elements	PeH;PeN

Contrast - Common

TR	3200 ms
TE	409 ms
MTC	Off
Magn. preparation	None
Fat suppr.	Fat sat.
Fat sat. mode	Strong
Blood suppr.	Off
Restore magn.	On

Contrast - Dynamic

Averages	1.4
Reconstruction	Magnitude
Measurements	1
Multiple series	Each measurement

Resolution - Common

FoV read	128 mm
FoV phase	150.0 %
Slice thickness	1.00 mm
Base resolution	128
Phase resolution	100 %
Slice resolution	100 %
Phase partial Fourier	Allowed
Slice partial Fourier	Off
Interpolation	Off

Resolution - iPAT

PAT mode	GRAPPA
Accel. factor PE	2
Ref. lines PE	24
Accel. factor 3D	2
Ref. lines 3D	24
Reference scan mode	Integrated

Resolution - Filter Image

Image Filter	Off
Distortion Corr.	Off
Prescan Normalize	On
Unfiltered images	Off
Normalize	Off
B1 filter	Off

Resolution - Filter Rawdata

Raw filter	On
Elliptical filter	Off

Geometry - Common

Slab group	1
Slabs	1
Position	R1.2 P36.9 H0.0 mm
Orientation	Sagittal
Phase enc. dir.	A >> P
Slice oversampling	0.0 %
Slices per slab	160
FoV read	128 mm
FoV phase	150.0 %
Slice thickness	1.00 mm
TR	3200 ms
Series	Interleaved
Concatenations	1

Geometry - AutoAlign

Slab group	1
Position	R1.2 P36.9 H0.0 mm
Orientation	Sagittal
Phase enc. dir.	A >> P
AutoAlign	---
Initial Position	R1.2 P36.9 H0.0
R	1.2 mm
P	36.9 mm
H	0.0 mm
Initial Rotation	0.00 deg
Initial Orientation	Sagittal

Geometry - Saturation

Fat suppr.	Fat sat.
Fat sat. mode	Strong
Restore magn.	On
Special sat.	None

Geometry - Navigator

Geometry - Tim Planning Suite

Set-n-Go Protocol	Off
Table position	H
Table position	0 mm
Inline Composing	Off

For peer review only - http://bmjopen.bmj.com/site/about/guidelines.xhtml

SIEMENS MAGNETOM Prisma

System - Miscellaneous

Positioning mode	REF
Table position	H
Table position	0 mm
MSMA	S - C - T
Sagittal	R >> L
Coronal	A >> P
Transversal	F >> H
Coil Combine Mode	Adaptive Combine
Save uncombined	Off
Matrix Optimization	Off
AutoAlign	---
Coil Select Mode	On - AutoCoilSelect

System - Adjustments

B0 Shim mode	Standard
B1 Shim mode	TrueForm
Adjust with body coil	Off
Confirm freq. adjustment	Off
Assume Dominant Fat	Off
Assume Silicone	Off
Adjustment Tolerance	Auto

System - Adjust Volume

Position	R1.2 P36.9 H0.0 mm
Orientation	Sagittal
Rotation	90.00 deg
F >> H	128 mm
A >> P	192 mm
R >> L	160 mm
Reset	Off

System - pTx Volumes

B1 Shim mode	TrueForm
Excitation	Non-sel.

System - Tx/Rx

Frequency 1H	123.244318 MHz
Correction factor	1
Gain	High
Img. Scale Cor.	3.000
Reset	Off
? Ref. amplitude 1H	0.000 V

Physio - Signal1

1st Signal/Mode	None
Trigger delay	0 ms
TR	3200 ms
Concatenations	1

Physio - Cardiac

Magn. preparation	None
Fat suppr.	Fat sat.
Dark blood	Off
FoV read	128 mm
FoV phase	150.0 %
Phase resolution	100 %

Physio - PACE

Resp. control	Off
Concatenations	1

Inline - Common

Subtract	Off

Inline - Common

Measurements	1
StdDev	Off
Save original images	On

Inline - MIP

MIP-Sag	Off
MIP-Cor	Off
MIP-Tra	Off
MIP-Time	Off
Save original images	On

Inline - Composing

Inline Composing	Off
Distortion Corr.	Off

Sequence - Part 1

Introduction	On
Dimension	3D
Elliptical scanning	Off
Reordering	Linear
Flow comp.	No
Echo spacing	4.4 ms
Adiabatic-mode	Off
Bandwidth	592 Hz/Px

Sequence - Part 2

Echo train duration	1034 ms
RF pulse type	Low SAR
Gradient mode	Whisper
Excitation	Non-sel.
Flip angle mode	T2 var
Turbo factor	282

Sequence - Assistant

Allowed delay	30 s

For peer review only

SIEMENS MAGNETOM Prisma

\\Study Protocols\BRAIN\Neonates\Theirworld - E161723\DTI_Neonate_v6b_dummy

TA: 0:28 PM: FIX Voxel size: 2.0×2.0×2.0 mmPAT: 4 Rel. SNR: 1.00 : epse

Properties

Prio recon	Off
Load images to viewer	On
Inline movie	Off
Auto store images	On
Load images to stamp segments	Off
Load images to graphic segments	Off
Auto open inline display	Off
Auto close inline display	Off
Start measurement without further preparation	Off
Wait for user to start	Off
Start measurements	Single measurement

Routine

Slice group	1
Slices	58
Dist. factor	0 %
Position	R1.2 P39.7 H47.8 mm
Orientation	Transversal
Phase enc. dir.	R >> L
AutoAlign	---
Phase oversampling	0 %
FoV read	256 mm
FoV phase	100.0 %
Slice thickness	2.0 mm
TR	3500 ms
TE	78.0 ms
Concatenations	1
Filter	Raw filter, Prescan Normalize
Coil elements	PeH;PeN

Contrast - Common

TR	3500 ms
TE	78.0 ms
MTC	Off
Magn. preparation	None
Fat suppr.	Fat sat.
Fat sat. mode	Strong

Contrast - Dynamic

Averaging mode	Long term
Reconstruction	Magnitude
Measurements	1
Delay in TR	0 ms
Multiple series	Off

Resolution - Common

FoV read	256 mm
FoV phase	100.0 %
Slice thickness	2.0 mm
Base resolution	128
Phase resolution	100 %
Phase partial Fourier	7/8
Interpolation	Off

Resolution - iPAT

Accel. mode	Slice accel.
Accel. factor PE	2
Ref. lines PE	40

Resolution - iPAT

Accel. factor slice	2
Reference scan mode	EPI/separate

Resolution - Filter Image

Distortion Corr.	Off
Prescan Normalize	On
Dynamic Field Corr.	Off

Resolution - Filter Rawdata

Raw filter	On
Elliptical filter	Off

Geometry - Common

Slice group	1
Slices	58
Dist. factor	0 %
Position	R1.2 P39.7 H47.8 mm
Orientation	Transversal
Phase enc. dir.	R >> L
FoV read	256 mm
FoV phase	100.0 %
Slice thickness	2.0 mm
TR	3500 ms
Multi-slice mode	Interleaved
Series	Interleaved
Concatenations	1

Geometry - AutoAlign

Slice group	1
Position	R1.2 P39.7 H47.8 mm
Orientation	Transversal
Phase enc. dir.	R >> L
AutoAlign	---
Initial Position	R1.2 P39.7 H47.8
R	1.2 mm
P	39.7 mm
H	47.8 mm
Initial Rotation	90.00 deg
Initial Orientation	Transversal

Geometry - Saturation

Fat suppr.	Fat sat.
Fat sat. mode	Strong
Special sat.	None

Geometry - Navigator

Geometry - Tim Planning Suite

Set-n-Go Protocol	Off
Table position	H
Table position	0 mm
Inline Composing	Off

System - Miscellaneous

Positioning mode	FIX
Table position	H
Table position	0 mm
MSMA	S - C - T
Sagittal	R >> L

For peer review only

For peer review only - http://bmjopen.bmj.com/site/about/guidelines.xhtml

SIEMENS MAGNETOM Prisma

System - Miscellaneous

Coronal	A >> P
Transversal	F >> H
Coil Combine Mode	Adaptive Combine
Matrix Optimization	Performance
AutoAlign	---
Coil Select Mode	On - AutoCoilSelect

System - Adjustments

B0 Shim mode	Standard
B1 Shim mode	TrueForm
Adjust with body coil	Off
Confirm freq. adjustment	Off
Assume Dominant Fat	Off
Assume Silicone	Off
Adjustment Tolerance	Auto

System - Adjust Volume

Position	R1.2 P39.7 H47.8 mm
Orientation	Transversal
Rotation	90.00 deg
R >> L	256 mm
A >> P	256 mm
F >> H	116 mm
Reset	Off

System - pTx Volumes

B1 Shim mode	TrueForm
Excitation	Standard

System - Tx/Rx

Frequency 1H	123.244318 MHz
Correction factor	1
Gain	High
Img. Scale Cor.	1.000
Reset	Off
? Ref. amplitude 1H	0.000 V

Physio - Signal1

1st Signal/Mode	None
TR	3500 ms
Concatenations	1

Physio - PACE

Resp. control	Off
Concatenations	1

Diff - Neuro

Diffusion mode	Free
Diff. directions	71
Diffusion Scheme	Monopolar
Diff. weightings	1
b-value	0 s/mm²
b-value	3
Diff. weighted images	On
Trace weighted images	Off
ADC maps	Off
FA maps	Off
Mosaic	Off
Tensor	Off
Noise level	40

Diff - Body

Diffusion mode	Free
Diff. directions	71
Diffusion Scheme	Monopolar
Diff. weightings	1
b-value	0 s/mm²
b-value	3
Diff. weighted images	On
Trace weighted images	Off
ADC maps	Off
Exponential ADC Maps	Off
FA maps	Off
Invert Gray Scale	Off
Calculated Image	Off
b-Value >=	0 s/mm²
Noise level	40

Diff - Composing

Inline Composing	Off
Distortion Corr.	Off

Sequence - Part 1

Introduction	Off
Optimization	None
Multi-slice mode	Interleaved
Free echo spacing	Off
Echo spacing	0.78 ms
Bandwidth	1446 Hz/Px

Sequence - Part 2

EPI factor	128
RF pulse type	Low SAR
Gradient mode	Normal
Excitation	Standard

Sequence - pTX Pulses

For peer review only

For peer review only - http://bmjopen.bmj.com/site/about/guidelines.xhtml

SIEMENS MAGNETOM Prisma

\\Study Protocols\BRAIN\Neonates\Theirworld - E161723\DTI_Neonate_v6b_rev

TA: 0:28 PM: FIX Voxel size: 2.0×2.0×2.0 mmPAT: 4 Rel. SNR: 1.00 : epse

Properties

Prio recon	Off
Load images to viewer	On
Inline movie	Off
Auto store images	On
Load images to stamp segments	Off
Load images to graphic segments	Off
Auto open inline display	Off
Auto close inline display	Off
Start measurement without further preparation	Off
Wait for user to start	Off
Start measurements	Single measurement

Routine

Slice group	1
Slices	58
Dist. factor	0 %
Position	R1.2 P39.7 H47.8 mm
Orientation	Transversal
Phase enc. dir.	R >> L
AutoAlign	---
Phase oversampling	0 %
FoV read	256 mm
FoV phase	100.0 %
Slice thickness	2.0 mm
TR	3500 ms
TE	78.0 ms
Concatenations	1
Filter	Raw filter, Prescan Normalize
Coil elements	PeH;PeN

Contrast - Common

TR	3500 ms
TE	78.0 ms
MTC	Off
Magn. preparation	None
Fat suppr.	Fat sat.
Fat sat. mode	Strong

Contrast - Dynamic

Averaging mode	Long term
Reconstruction	Magnitude
Measurements	1
Delay in TR	0 ms
Multiple series	Off

Resolution - Common

FoV read	256 mm
FoV phase	100.0 %
Slice thickness	2.0 mm
Base resolution	128
Phase resolution	100 %
Phase partial Fourier	7/8
Interpolation	Off

Resolution - iPAT

Accel. mode	Slice accel.
Accel. factor PE	2
Ref. lines PE	40

Resolution - iPAT

Accel. factor slice	2
Reference scan mode	EPI/separate

Resolution - Filter Image

Distortion Corr.	Off
Prescan Normalize	On
Dynamic Field Corr.	Off

Resolution - Filter Rawdata

Raw filter	On
Elliptical filter	Off

Geometry - Common

Slice group	1
Slices	58
Dist. factor	0 %
Position	R1.2 P39.7 H47.8 mm
Orientation	Transversal
Phase enc. dir.	R >> L
FoV read	256 mm
FoV phase	100.0 %
Slice thickness	2.0 mm
TR	3500 ms
Multi-slice mode	Interleaved
Series	Interleaved
Concatenations	1

Geometry - AutoAlign

Slice group	1
Position	R1.2 P39.7 H47.8 mm
Orientation	Transversal
Phase enc. dir.	R >> L
AutoAlign	---
Initial Position	R1.2 P39.7 H47.8
R	1.2 mm
P	39.7 mm
H	47.8 mm
Initial Rotation	90.00 deg
Initial Orientation	Transversal

Geometry - Saturation

Fat suppr.	Fat sat.
Fat sat. mode	Strong
Special sat.	None

Geometry - Navigator

Geometry - Tim Planning Suite

Set-n-Go Protocol	Off
Table position	H
Table position	0 mm
Inline Composing	Off

System - Miscellaneous

Positioning mode	FIX
Table position	H
Table position	0 mm
MSMA	S - C - T
Sagittal	R >> L

For peer review only

For peer review only - http://bmjopen.bmj.com/site/about/guidelines.xhtml

SIEMENS MAGNETOM Prisma

System - Miscellaneous

Coronal	A >> P
Transversal	F >> H
Coil Combine Mode	Adaptive Combine
Matrix Optimization	Performance
AutoAlign	---
Coil Select Mode	On - AutoCoilSelect

System - Adjustments

B0 Shim mode	Standard
B1 Shim mode	TrueForm
Adjust with body coil	Off
Confirm freq. adjustment	Off
Assume Dominant Fat	Off
Assume Silicone	Off
Adjustment Tolerance	Auto

System - Adjust Volume

Position	R1.2 P39.7 H47.8 mm
Orientation	Transversal
Rotation	90.00 deg
R >> L	256 mm
A >> P	256 mm
F >> H	116 mm
Reset	Off

System - pTx Volumes

B1 Shim mode	TrueForm
Excitation	Standard

System - Tx/Rx

Frequency 1H	123.244318 MHz
Correction factor	1
Gain	High
Img. Scale Cor.	1.000
Reset	Off
? Ref. amplitude 1H	0.000 V

Physio - Signal1

1st Signal/Mode	None
TR	3500 ms
Concatenations	1

Physio - PACE

Resp. control	Off
Concatenations	1

Diff - Neuro

Diffusion mode	MDDW
Diff. directions	6
Diffusion Scheme	Monopolar
Diff. weightings	1
b-value	0 s/mm²
b-value	3
Diff. weighted images	On
Trace weighted images	Off
ADC maps	Off
FA maps	Off
Mosaic	Off
Tensor	Off
Noise level	40

Diff - Body

Diffusion mode	MDDW
Diff. directions	6
Diffusion Scheme	Monopolar
Diff. weightings	1
b-value	0 s/mm²
b-value	3
Diff. weighted images	On
Trace weighted images	Off
ADC maps	Off
Exponential ADC Maps	Off
FA maps	Off
Invert Gray Scale	Off
Calculated Image	Off
b-Value >=	0 s/mm²
Noise level	40

Diff - Composing

Inline Composing	Off
Distortion Corr.	Off

Sequence - Part 1

Introduction	Off
Optimization	None
Multi-slice mode	Interleaved
Free echo spacing	Off
Echo spacing	0.78 ms
Bandwidth	1446 Hz/Px

Sequence - Part 2

EPI factor	128
RF pulse type	Low SAR
Gradient mode	Normal
Excitation	Standard

Sequence - pTX Pulses

For peer review only

For peer review only - http://bmjopen.bmj.com/site/about/guidelines.xhtml

SIEMENS MAGNETOM Prisma

\\Study Protocols\BRAIN\Neonates\Theirworld - E161723\DTI_Neonate_v6b_pt1

TA: 4:29 PM: FIX Voxel size: 2.0×2.0×2.0 mmPAT: 4 Rel. SNR: 1.00 : epse

Properties

Prio recon	Off
Load images to viewer	On
Inline movie	Off
Auto store images	On
Load images to stamp segments	Off
Load images to graphic segments	Off
Auto open inline display	Off
Auto close inline display	Off
Start measurement without further preparation	Off
Wait for user to start	Off
Start measurements	Single measurement

Routine

Slice group	1
Slices	58
Dist. factor	0 %
Position	R1.2 P39.7 H47.8 mm
Orientation	Transversal
Phase enc. dir.	L >> R
AutoAlign	---
Phase oversampling	0 %
FoV read	256 mm
FoV phase	100.0 %
Slice thickness	2.0 mm
TR	3500 ms
TE	78.0 ms
Averages	1
Concatenations	1
Filter	Raw filter, Prescan Normalize
Coil elements	PeH;PeN

Contrast - Common

TR	3500 ms
TE	78.0 ms
MTC	Off
Magn. preparation	None
Fat suppr.	Fat sat.
Fat sat. mode	Strong

Contrast - Dynamic

Averages	1
Averaging mode	Long term
Reconstruction	Magnitude
Measurements	1
Delay in TR	0 ms
Multiple series	Off

Resolution - Common

FoV read	256 mm
FoV phase	100.0 %
Slice thickness	2.0 mm
Base resolution	128
Phase resolution	100 %
Phase partial Fourier	7/8
Interpolation	Off

Resolution - iPAT

Accel. mode	Slice accel.

Resolution - iPAT

Accel. factor PE	2
Ref. lines PE	40
Accel. factor slice	2
Reference scan mode	EPI/separate

Resolution - Filter Image

Distortion Corr.	Off
Prescan Normalize	On
Dynamic Field Corr.	Off

Resolution - Filter Rawdata

Raw filter	On
Elliptical filter	Off

Geometry - Common

Slice group	1
Slices	58
Dist. factor	0 %
Position	R1.2 P39.7 H47.8 mm
Orientation	Transversal
Phase enc. dir.	L >> R
FoV read	256 mm
FoV phase	100.0 %
Slice thickness	2.0 mm
TR	3500 ms
Multi-slice mode	Interleaved
Series	Interleaved
Concatenations	1

Geometry - AutoAlign

Slice group	1
Position	R1.2 P39.7 H47.8 mm
Orientation	Transversal
Phase enc. dir.	L >> R
AutoAlign	---
Initial Position	R1.2 P39.7 H47.8
R	1.2 mm
P	39.7 mm
H	47.8 mm
Initial Rotation	-90.00 deg
Initial Orientation	Transversal

Geometry - Saturation

Fat suppr.	Fat sat.
Fat sat. mode	Strong
Special sat.	None

Geometry - Navigator

Geometry - Tim Planning Suite

Set-n-Go Protocol	Off
Table position	H
Table position	0 mm
Inline Composing	Off

System - Miscellaneous

Positioning mode	FIX
Table position	H
Table position	0 mm

For peer review only

SIEMENS MAGNETOM Prisma

System - Miscellaneous

MSMA	S - C - T
Sagittal	R >> L
Coronal	A >> P
Transversal	F >> H
Coil Combine Mode	Adaptive Combine
Matrix Optimization	Performance
AutoAlign	---
Coil Select Mode	On - AutoCoilSelect

System - Adjustments

B0 Shim mode	Standard
B1 Shim mode	TrueForm
Adjust with body coil	Off
Confirm freq. adjustment	Off
Assume Dominant Fat	Off
Assume Silicone	Off
Adjustment Tolerance	Auto

System - Adjust Volume

Position	R1.2 P39.7 H47.8 mm
Orientation	Transversal
Rotation	-90.00 deg
R >> L	256 mm
A >> P	256 mm
F >> H	116 mm
Reset	Off

System - pTx Volumes

B1 Shim mode	TrueForm
Excitation	Standard

System - Tx/Rx

Frequency 1H	123.244318 MHz
Correction factor	1
Gain	High
Img. Scale Cor.	1.000
Reset	Off
? Ref. amplitude 1H	0.000 V

Physio - Signal1

1st Signal/Mode	None
TR	3500 ms
Concatenations	1

Physio - PACE

Resp. control	Off
Concatenations	1

Diff - Neuro

Diffusion mode	Free
Diff. directions	71
Diffusion Scheme	Monopolar
Diff. weightings	2
b-value 1	0 s/mm²
b-value 2	750 s/mm²
b-value 1	1
b-value 2	1
Diff. weighted images	On
Trace weighted images	Off
ADC maps	Off
FA maps	Off
Mosaic	On
Tensor	Off

Diff - Neuro

Noise level	40

Diff - Body

Diffusion mode	Free
Diff. directions	71
Diffusion Scheme	Monopolar
Diff. weightings	2
b-value 1	0 s/mm²
b-value 2	750 s/mm²
b-value 1	1
b-value 2	1
Diff. weighted images	On
Trace weighted images	Off
ADC maps	Off
Exponential ADC Maps	Off
FA maps	Off
Invert Gray Scale	Off
Calculated Image	Off
b-Value >=	0 s/mm²
Noise level	40

Diff - Composing

Inline Composing	Off
Distortion Corr.	Off

Sequence - Part 1

Introduction	Off
Optimization	None
Multi-slice mode	Interleaved
Free echo spacing	Off
Echo spacing	0.78 ms
Bandwidth	1446 Hz/Px

Sequence - Part 2

EPI factor	128
RF pulse type	Low SAR
Gradient mode	Normal
Excitation	Standard

Sequence - pTX Pulses

For peer review only

SIEMENS MAGNETOM Prisma

\\Study Protocols\BRAIN\Neonates\Theirworld - E161723\DTI_Neonate_v6b_pt2
TA: 5:01 PM: FIX Voxel size: 2.0×2.0×2.0 mmPAT: 4 Rel. SNR: 1.00 : epse

Properties

Prio recon	Off
Load images to viewer	On
Inline movie	Off
Auto store images	On
Load images to stamp segments	Off
Load images to graphic segments	Off
Auto open inline display	Off
Auto close inline display	Off
Start measurement without further preparation	Off
Wait for user to start	Off
Start measurements	Single measurement

Routine

Slice group	1
Slices	58
Dist. factor	0 %
Position	R1.2 P39.7 H47.8 mm
Orientation	Transversal
Phase enc. dir.	L >> R
AutoAlign	---
Phase oversampling	0 %
FoV read	256 mm
FoV phase	100.0 %
Slice thickness	2.0 mm
TR	3500 ms
TE	78.0 ms
Averages	1
Concatenations	1
Filter	Raw filter, Prescan Normalize
Coil elements	PeH;PeN

Contrast - Common

TR	3500 ms
TE	78.0 ms
MTC	Off
Magn. preparation	None
Fat suppr.	Fat sat.
Fat sat. mode	Strong

Contrast - Dynamic

Averages	1
Averaging mode	Long term
Reconstruction	Magnitude
Measurements	1
Delay in TR	0 ms
Multiple series	Off

Resolution - Common

FoV read	256 mm
FoV phase	100.0 %
Slice thickness	2.0 mm
Base resolution	128
Phase resolution	100 %
Phase partial Fourier	7/8
Interpolation	Off

Resolution - iPAT

Accel. mode	Slice accel.

Resolution - iPAT

Accel. factor PE	2
Ref. lines PE	40
Accel. factor slice	2
Reference scan mode	EPI/separate

Resolution - Filter Image

Distortion Corr.	Off
Prescan Normalize	On
Dynamic Field Corr.	Off

Resolution - Filter Rawdata

Raw filter	On
Elliptical filter	Off

Geometry - Common

Slice group	1
Slices	58
Dist. factor	0 %
Position	R1.2 P39.7 H47.8 mm
Orientation	Transversal
Phase enc. dir.	L >> R
FoV read	256 mm
FoV phase	100.0 %
Slice thickness	2.0 mm
TR	3500 ms
Multi-slice mode	Interleaved
Series	Interleaved
Concatenations	1

Geometry - AutoAlign

Slice group	1
Position	R1.2 P39.7 H47.8 mm
Orientation	Transversal
Phase enc. dir.	L >> R
AutoAlign	---
Initial Position	R1.2 P39.7 H47.8
R	1.2 mm
P	39.7 mm
H	47.8 mm
Initial Rotation	-90.00 deg
Initial Orientation	Transversal

Geometry - Saturation

Fat suppr.	Fat sat.
Fat sat. mode	Strong
Special sat.	None

Geometry - Navigator

Geometry - Tim Planning Suite

Set-n-Go Protocol	Off
Table position	H
Table position	0 mm
Inline Composing	Off

System - Miscellaneous

Positioning mode	FIX
Table position	H
Table position	0 mm

For peer review only

SIEMENS MAGNETOM Prisma

System - Miscellaneous

MSMA	S - C - T
Sagittal	R >> L
Coronal	A >> P
Transversal	F >> H
Coil Combine Mode	Adaptive Combine
Matrix Optimization	Performance
AutoAlign	---
Coil Select Mode	On - AutoCoilSelect

System - Adjustments

B0 Shim mode	Standard
B1 Shim mode	TrueForm
Adjust with body coil	Off
Confirm freq. adjustment	Off
Assume Dominant Fat	Off
Assume Silicone	Off
Adjustment Tolerance	Auto

System - Adjust Volume

Position	R1.2 P39.7 H47.8 mm
Orientation	Transversal
Rotation	-90.00 deg
R >> L	256 mm
A >> P	256 mm
F >> H	116 mm
Reset	Off

System - pTx Volumes

B1 Shim mode	TrueForm
Excitation	Standard

System - Tx/Rx

Frequency 1H	123.244318 MHz
Correction factor	1
Gain	High
Img. Scale Cor.	1.000
Reset	Off
? Ref. amplitude 1H	0.000 V

Physio - Signal1

1st Signal/Mode	None
TR	3500 ms
Concatenations	1

Physio - PACE

Resp. control	Off
Concatenations	1

Diff - Neuro

Diffusion mode	Free
Diff. directions	80
Diffusion Scheme	Monopolar
Diff. weightings	2
b-value 1	0 s/mm²
b-value 2	2500 s/mm²
b-value 1	1
b-value 2	1
Diff. weighted images	On
Trace weighted images	Off
ADC maps	Off
FA maps	Off
Mosaic	On
Tensor	Off

Diff - Neuro

Noise level	40

Diff - Body

Diffusion mode	Free
Diff. directions	80
Diffusion Scheme	Monopolar
Diff. weightings	2
b-value 1	0 s/mm²
b-value 2	2500 s/mm²
b-value 1	1
b-value 2	1
Diff. weighted images	On
Trace weighted images	Off
ADC maps	Off
Exponential ADC Maps	Off
FA maps	Off
Invert Gray Scale	Off
Calculated Image	Off
b-Value >=	0 s/mm²
Noise level	40

Diff - Composing

Inline Composing	Off
Distortion Corr.	Off

Sequence - Part 1

Introduction	Off
Optimization	None
Multi-slice mode	Interleaved
Free echo spacing	Off
Echo spacing	0.78 ms
Bandwidth	1446 Hz/Px

Sequence - Part 2

EPI factor	128
RF pulse type	Low SAR
Gradient mode	Normal
Excitation	Standard

Sequence - pTX Pulses

For peer review only

For peer review only - http://bmjopen.bmj.com/site/about/guidelines.xhtml

SIEMENS MAGNETOM Prisma

\\Study Protocols\BRAIN\Neonates\Theirworld - E161723\MPRAGE-v4

TA: 3:09 PM: FIX Voxel size: 1.0×1.0×1.0 mmPAT: 2 Rel. SNR: 1.00 : tfl

Properties

Prio recon	Off
Load images to viewer	On
Inline movie	Off
Auto store images	On
Load images to stamp segments	On
Load images to graphic segments	Off
Auto open inline display	Off
Auto close inline display	Off
Start measurement without further preparation	Off
Wait for user to start	Off
Start measurements	Single measurement

Routine

Slab group	1
Slabs	1
Dist. factor	50 %
Position	R1.1 P38.9 F20.7 mm
Orientation	Sagittal
Phase enc. dir.	A >> P
AutoAlign	---
Phase oversampling	20 %
Slice oversampling	0.0 %
Slices per slab	160
FoV read	160 mm
FoV phase	100.0 %
Slice thickness	1.00 mm
TR	1970.0 ms
TE	4.69 ms
Averages	1
Concatenations	1
Filter	Prescan Normalize
Coil elements	PeH;PeN;SP1

Contrast - Common

TR	1970.0 ms
TE	4.69 ms
Magn. preparation	Non-sel. IR
TI	1100 ms
Flip angle	9 deg
Fat suppr.	None
Water suppr.	None

Contrast - Dynamic

Averages	1
Averaging mode	Long term
Reconstruction	Magnitude
Measurements	1
Multiple series	Each measurement

Resolution - Common

FoV read	160 mm
FoV phase	100.0 %
Slice thickness	1.00 mm
Base resolution	160
Phase resolution	100 %
Slice resolution	100 %
Phase partial Fourier	7/8
Slice partial Fourier	Off
Interpolation	Off

Resolution - iPAT

PAT mode	GRAPPA
Accel. factor PE	2
Ref. lines PE	24
Accel. factor 3D	1
Reference scan mode	Integrated

Resolution - Filter Image

Image Filter	Off
Distortion Corr.	Off
Prescan Normalize	On
Unfiltered images	Off
Normalize	Off
B1 filter	Off

Resolution - Filter Rawdata

Raw filter	Off
Elliptical filter	Off

Geometry - Common

Slab group	1
Slabs	1
Dist. factor	50 %
Position	R1.1 P38.9 F20.7 mm
Orientation	Sagittal
Phase enc. dir.	A >> P
Slice oversampling	0.0 %
Slices per slab	160
FoV read	160 mm
FoV phase	100.0 %
Slice thickness	1.00 mm
TR	1970.0 ms
Multi-slice mode	Single shot
Series	Interleaved
Concatenations	1

Geometry - AutoAlign

Slab group	1
Position	R1.1 P38.9 F20.7 mm
Orientation	Sagittal
Phase enc. dir.	A >> P
AutoAlign	---
Initial Position	R1.1 P38.9 F20.7
R	1.1 mm
P	38.9 mm
F	20.7 mm
Initial Rotation	0.00 deg
Initial Orientation	Sagittal

Geometry - Navigator

Geometry - Tim Planning Suite

Set-n-Go Protocol	Off
Table position	H
Table position	0 mm
Inline Composing	Off

System - Miscellaneous

Positioning mode	FIX
Table position	H
Table position	0 mm

For peer review only

SIEMENS MAGNETOM Prisma

System - Miscellaneous

MSMA	S - C - T
Sagittal	R >> L
Coronal	A >> P
Transversal	F >> H
Coil Combine Mode	Adaptive Combine
Save uncombined	Off
Matrix Optimization	Off
Coil Focus	Flat
AutoAlign	---
Coil Select Mode	On - AutoCoilSelect

System - Adjustments

B0 Shim mode	Standard
B1 Shim mode	TrueForm
Adjust with body coil	Off
Confirm freq. adjustment	Off
Assume Dominant Fat	Off
Assume Silicone	Off
Adjustment Tolerance	Auto

System - Adjust Volume

Position	R1.1 P38.9 F20.7 mm
Orientation	Sagittal
Rotation	0.00 deg
A >> P	160 mm
F >> H	160 mm
R >> L	160 mm
Reset	Off

System - pTx Volumes

B1 Shim mode	TrueForm
Excitation	Non-sel.

System - Tx/Rx

Frequency 1H	123.244318 MHz
Correction factor	1
Gain	Low
Img. Scale Cor.	4.000
Reset	Off
? Ref. amplitude 1H	0.000 V

Physio - Signal1

1st Signal/Mode	None
TR	1970.0 ms
Concatenations	1

Physio - Cardiac

Magn. preparation	Non-sel. IR
TI	1100 ms
Fat suppr.	None
Dark blood	Off
FoV read	160 mm
FoV phase	100.0 %
Phase resolution	100 %

Physio - PACE

Resp. control	Off
Concatenations	1

Inline - Common

Subtract	Off
Measurements	1
StdDev	Off

Inline - Common

Save original images	On

Inline - MIP

MIP-Sag	Off
MIP-Cor	Off
MIP-Tra	Off
MIP-Time	Off
Save original images	On

Inline - Composing

Inline Composing	Off
Distortion Corr.	Off

Inline - MapIt

Save original images	On
MapIt	None
Flip angle	9 deg
Measurements	1
TR	1970.0 ms
TE	4.69 ms

Sequence - Part 1

Introduction	On
Dimension	3D
Elliptical scanning	Off
Reordering	Linear
Asymmetric echo	Off
Flow comp.	No
Multi-slice mode	Single shot
Echo spacing	10.8 ms
Bandwidth	140 Hz/Px

Sequence - Part 2

RF pulse type	Normal
Gradient mode	Whisper
Excitation	Non-sel.
RF spoiling	On
Incr. Gradient spoiling	Off
Turbo factor	160

Sequence - Assistant

Mode	Off

For peer review only

For peer review only - http://bmjopen.bmj.com/site/about/guidelines.xhtml

SIEMENS MAGNETOM Prisma

\\Study Protocols\BRAIN\Neonates\Theirworld - E161723\SWI_v2

TA: 2:23 PM: FIX Voxel size: 0.8×0.8×3.0 mmPAT: 3 Rel. SNR: 1.00 : qswi_r

Properties

Prio recon	Off
Load images to viewer	On
Inline movie	Off
Auto store images	On
Load images to stamp segments	Off
Load images to graphic segments	Off
Auto open inline display	Off
Auto close inline display	Off
Start measurement without further preparation	Off
Wait for user to start	Off
Start measurements	Single measurement

Routine

Slab group	1
Slabs	1
Dist. factor	20 %
Position	L0.0 A2.3 H2.2 mm
Orientation	Transversal
Phase enc. dir.	R >> L
AutoAlign	---
Phase oversampling	0 %
Slice oversampling	20.0 %
Slices per slab	40
FoV read	240 mm
FoV phase	84.4 %
Slice thickness	3.00 mm
TR	28.0 ms
TE	20.00 ms
Averages	1
Concatenations	1
Filter	Prescan Normalize
Coil elements	HEA;HEP

Contrast - Common

TR	28.0 ms
TE	20.00 ms
MTC	Off
Magn. preparation	None
Flip angle	9 deg
Fat suppr.	None
Water suppr.	None
SWI	On

Contrast - Dynamic

Averages	1
Averaging mode	Short term
Reconstruction	Magn./Phase
Measurements	1
Multiple series	Each measurement

Resolution - Common

FoV read	240 mm
FoV phase	84.4 %
Slice thickness	3.00 mm
Base resolution	320
Phase resolution	100 %
Slice resolution	100 %
Phase partial Fourier	Off
Slice partial Fourier	Off

Resolution - Common

Interpolation	Off

Resolution - iPAT

PAT mode	GRAPPA
Accel. factor PE	3
Ref. lines PE	24
Accel. factor 3D	1
Reference scan mode	Integrated

Resolution - Filter Image

Image Filter	Off
Distortion Corr.	Off
Prescan Normalize	On
Unfiltered images	Off
Normalize	Off
B1 filter	Off

Resolution - Filter Rawdata

Raw filter	Off
Elliptical filter	Off

Geometry - Common

Slab group	1
Slabs	1
Dist. factor	20 %
Position	L0.0 A2.3 H2.2 mm
Orientation	Transversal
Phase enc. dir.	R >> L
Slice oversampling	20.0 %
Slices per slab	40
FoV read	240 mm
FoV phase	84.4 %
Slice thickness	3.00 mm
TR	28.0 ms
Multi-slice mode	Interleaved
Series	Interleaved
Concatenations	1

Geometry - AutoAlign

Slab group	1
Position	L0.0 A2.3 H2.2 mm
Orientation	Transversal
Phase enc. dir.	R >> L
AutoAlign	---
Initial Position	L0.0 A2.3 H2.2
L	0.0 mm
A	2.3 mm
H	2.2 mm
Initial Rotation	89.61 deg
Initial Orientation	Transversal

Geometry - Saturation

Saturation mode	Standard
Fat suppr.	None
Water suppr.	None
Special sat.	None

Geometry - Tim Planning Suite

Set-n-Go Protocol	Off
Table position	H

For peer review only - http://bmjopen.bmj.com/site/about/guidelines.xhtml

SIEMENS MAGNETOM Prisma

Geometry - Tim Planning Suite

Table position	0 mm
Inline Composing	Off

System - Miscellaneous

Positioning mode	FIX
Table position	H
Table position	0 mm
MSMA	S - C - T
Sagittal	R >> L
Coronal	A >> P
Transversal	F >> H
Coil Combine Mode	Adaptive Combine
Save uncombined	Off
Matrix Optimization	Off
AutoAlign	---
Coil Select Mode	On - AutoCoilSelect

System - Adjustments

B0 Shim mode	Standard
B1 Shim mode	TrueForm
Adjust with body coil	Off
Confirm freq. adjustment	Off
Assume Dominant Fat	Off
Assume Silicone	Off
Adjustment Tolerance	Auto

System - Adjust Volume

Position	L0.0 A2.3 H2.2 mm
Orientation	Transversal
Rotation	89.61 deg
R >> L	203 mm
A >> P	240 mm
F >> H	120 mm
Reset	Off

System - pTx Volumes

B1 Shim mode	TrueForm
Excitation	Slab-sel.

System - Tx/Rx

Frequency 1H	123.244318 MHz
Correction factor	1
Gain	Low
Img. Scale Cor.	1.000
Reset	Off
? Ref. amplitude 1H	0.000 V

Physio - Signal1

1st Signal/Mode	None
TR	28.0 ms
Concatenations	1
Segments	1

Physio - Cardiac

Tagging	None
Magn. preparation	None
Fat suppr.	None
Dark blood	Off
FoV read	240 mm
FoV phase	84.4 %
Phase resolution	100 %

Physio - PACE

Resp. control	Off
Concatenations	1

Inline - Common

Subtract	Off
Measurements	1
StdDev	Off
Liver registration	Off
Save original images	On

Inline - MIP

MIP-Sag	Off
MIP-Cor	Off
MIP-Tra	Off
MIP-Time	Off
Save original images	On

Inline - Soft Tissue

Wash - In	Off
Wash - Out	Off
TTP	Off
PEI	Off
MIP - time	Off
Measurements	1

Inline - Composing

Inline Composing	Off
Distortion Corr.	Off

Inline - MapIt

Save original images	On
MapIt	None
Flip angle	9 deg
Measurements	1
Contrasts	1
TR	28.0 ms
TE	20.00 ms

Sequence - Part 1

Introduction	On
Dimension	3D
Elliptical scanning	Off
Phase stabilisation	Off
Asymmetric echo	Off
Contrasts	1
Flow comp.	Yes
Multi-slice mode	Interleaved
Bandwidth	120 Hz/Px

Sequence - Part 2

Segments	1
Acoustic noise reduction	Active
RF pulse type	Fast
Gradient mode	Whisper
Excitation	Slab-sel.
RF spoiling	On

Sequence - Assistant

Mode	Off
Allowed delay	30 s

For peer review only

For peer review only - http://bmjopen.bmj.com/site/about/guidelines.xhtml

SIEMENS MAGNETOM Prisma

\\Study Protocols\BRAIN\Neonates\Theirworld - E161723\t2_blade_dark-fluid_tra_v3

TA: 3:22 PM: REF Voxel size: 0.9×0.9×3.0 mmPAT: 2 Rel. SNR: 1.00 : qtirB_rr

Properties

Prio recon	Off
Load images to viewer	On
Inline movie	Off
Auto store images	On
Load images to stamp segments	On
Load images to graphic segments	Off
Auto open inline display	Off
Auto close inline display	Off
Start measurement without further preparation	Off
Wait for user to start	Off
Start measurements	Single measurement

Routine

Slice group	1
Slices	40
Dist. factor	0 %
Position	Isocenter
Orientation	Transversal
Phase enc. dir.	R >> L
AutoAlign	---
Phase oversampling	0.0 %
FoV read	240 mm
FoV phase	100.0 %
Slice thickness	3.0 mm
TR	10000.0 ms
TE	130 ms
Averages	1
Concatenations	2
Filter	Prescan Normalize
Coil elements	HEA;HEP

Contrast - Common

TR	10000.0 ms
TE	130 ms
TD	0.0 ms
MTC	Off
Magn. preparation	Slice-sel. IR
TI	2606 ms
Flip angle	130 deg
Fat suppr.	Fat sat.
Fat sat. mode	Strong
Water suppr.	None
Restore magn.	Off
Freeze suppressed tissue	On

Contrast - Dynamic

Averages	1
Averaging mode	Short term
Reconstruction	Magnitude
Measurements	1
Multiple series	Each measurement

Resolution - Common

FoV read	240 mm
FoV phase	100.0 %
Slice thickness	3.0 mm
Base resolution	256
BLADE coverage	100.0 %
Trajectory	BLADE

Resolution - Common

Interpolation	Off

Resolution - iPAT

PAT mode	GRAPPA
Accel. factor PE	2
Ref. lines PE	8
Reference scan mode	Integrated

Resolution - Filter Image

Image Filter	Off
Distortion Corr.	Off
Prescan Normalize	On
Unfiltered images	Off
Normalize	Off
B1 filter	Off

Resolution - Filter Rawdata

Raw filter	Off
Elliptical filter	Off

Geometry - Common

Slice group	1
Slices	40
Dist. factor	0 %
Position	Isocenter
Orientation	Transversal
Phase enc. dir.	R >> L
FoV read	240 mm
FoV phase	100.0 %
Slice thickness	3.0 mm
TR	10000.0 ms
Multi-slice mode	Interleaved
Series	Interleaved
Concatenations	2

Geometry - AutoAlign

Slice group	1
Position	Isocenter
Orientation	Transversal
Phase enc. dir.	R >> L
AutoAlign	---
Initial Position	Isocenter
L	0.0 mm
P	0.0 mm
H	0.0 mm
Initial Rotation	90.00 deg
Initial Orientation	Transversal

Geometry - Saturation

Fat suppr.	Fat sat.
Fat sat. mode	Strong
Water suppr.	None
Restore magn.	Off
Special sat.	Parallel F
Gap	10 mm
Thickness	70 mm

Geometry - Navigator

For peer review only - http://bmjopen.bmj.com/site/about/guidelines.xhtml

SIEMENS MAGNETOM Prisma

Geometry - Tim Planning Suite

Set-n-Go Protocol	Off
Table position	H
Table position	0 mm
Inline Composing	Off

System - Miscellaneous

Positioning mode	REF
Table position	H
Table position	0 mm
MSMA	S - C - T
Sagittal	R >> L
Coronal	A >> P
Transversal	F >> H
Coil Combine Mode	Adaptive Combine
Save uncombined	Off
Matrix Optimization	Off
AutoAlign	---
Coil Select Mode	On - AutoCoilSelect

System - Adjustments

B0 Shim mode	Standard
B1 Shim mode	TrueForm
Adjust with body coil	Off
Confirm freq. adjustment	Off
Assume Dominant Fat	Off
Assume Silicone	Off
Adjustment Tolerance	Auto

System - Adjust Volume

Position	Isocenter
Orientation	Transversal
Rotation	90.00 deg
R >> L	240 mm
A >> P	240 mm
F >> H	120 mm
Reset	Off

System - pTx Volumes

B1 Shim mode	TrueForm

System - Tx/Rx

Frequency 1H	123.244318 MHz
Correction factor	1
Gain	High
Img. Scale Cor.	1.000
Reset	Off
? Ref. amplitude 1H	0.000 V

Physio - Signal1

1st Signal/Mode	None
TR	10000.0 ms
Concatenations	2

Physio - Cardiac

Magn. preparation	Slice-sel. IR
TI	2606 ms
Fat suppr.	Fat sat.
Dark blood	Off
FoV read	240 mm
FoV phase	100.0 %
BLADE coverage	100.0 %
Trajectory	BLADE

Physio - PACE

Resp. control	Off
Concatenations	2

Inline - Common

Subtract	Off
Measurements	1
StdDev	Off
Save original images	On

Inline - MIP

MIP-Sag	Off
MIP-Cor	Off
MIP-Tra	Off
MIP-Time	Off
Save original images	On

Inline - Composing

Inline Composing	Off
Distortion Corr.	Off

Sequence - Part 1

Introduction	On
Dimension	2D
Compensate T2 decay	Off
Contrasts	1
Flow comp.	Read
Multi-slice mode	Interleaved
Free echo spacing	Off
Echo spacing	8.64 ms
Bandwidth	362 Hz/Px

Sequence - Part 2

Define	Turbo factor
Echo trains per slice	9
Phase correction	Automatic
Acoustic noise reduction	Active
RF pulse type	Low SAR
Gradient mode	Normal
Hyperecho	Off
WARP	Off
Motion correction	On
Red. EC sensitivity	Off
Turbo factor	28

Sequence - Assistant

Mode	Min flip angle
Min flip angle	130 deg
Allowed delay	30 s

For peer review only

For peer review only - http://bmjopen.bmj.com/site/about/guidelines.xhtml

```
# Author: qspace2siemens.m (Michael Thrippleton), manually edited
into 2 parts
# Source file: ./vector_tables/neonate/04-shells-3-6-64-64.txt
# b-value at UI: 750
# non-zero b-values: 750
# number of non-zero shells: 1
# number of directions per non-zero shell: 64
# number of b=0 volumes: 7
# total number of directions including b0: 71
[directions=71]
normalization = none
coordinatesystem = xyz
comment=bUI: 750, b: 750, Nb0: 7
vector[0] = ( 0.000000, 0.000000, 0.000000 )
vector[1] = ( -0.538981, 0.033731, -0.091439 )
vector[2] = ( -0.000440, 0.429608, 0.339760 )
vector[3] = ( -0.147395, -0.494556, -0.183546 )
vector[4] = ( 0.239035, -0.347062, 0.349872 )
vector[5] = ( -0.016278, -0.195328, 0.511451 )
vector[6] = ( -0.061295, -0.451376, 0.304143 )
vector[7] = ( 0.025626, -0.008709, -0.547053 )
vector[8] = ( -0.231133, -0.471788, 0.154896 )
vector[9] = ( -0.397538, -0.105537, -0.361699 )
vector[10] = ( 0.447399, -0.280126, -0.146162 )
vector[11] = ( 0.000000, 0.000000, 0.000000 )
vector[12] = ( -0.347344, -0.305418, 0.293379 )
vector[13] = ( 0.195148, -0.224679, 0.459823 )
vector[14] = ( 0.219722, 0.401006, -0.301523 )
vector[15] = ( 0.496386, 0.051099, 0.225809 )
vector[16] = ( -0.490022, 0.181524, -0.164098 )
vector[17] = ( 0.415886, 0.250359, 0.253691 )
vector[18] = ( 0.293795, 0.319409, 0.334159 )
vector[19] = ( 0.446457, -0.091032, 0.303955 )
vector[20] = ( 0.218923, -0.268898, -0.423989 )
vector[21] = ( -0.245685, -0.236576, 0.428568 )
vector[22] = ( 0.000000, 0.000000, 0.000000 )
vector[23] = ( 0.023434, -0.514342, -0.186823 )
vector[24] = ( 0.210090, -0.495890, -0.099773 )
vector[25] = ( 0.127918, 0.282591, 0.451419 )
vector[26] = ( -0.497742, -0.190842, -0.125826 )
vector[27] = ( -0.352216, -0.116300, 0.403012 )
vector[28] = ( -0.439047, 0.004691, 0.327438 )
vector[29] = ( 0.143700, -0.138995, -0.509932 )
vector[30] = ( -0.483604, 0.256940, -0.010438 )
vector[31] = ( 0.536886, 0.108072, -0.008594 )
vector[32] = ( -0.113008, -0.337640, 0.416207 )
vector[33] = ( 0.000000, 0.000000, 0.000000 )
vector[34] = ( 0.346021, -0.402459, -0.135263 )
vector[35] = ( -0.172278, 0.446108, 0.267035 )
vector[36] = ( -0.309270, 0.076830, -0.445476 )
vector[37] = ( 0.274066, -0.423055, 0.214272 )
vector[38] = ( 0.052227, -0.321802, 0.440132 )
vector[39] = ( 0.075465, 0.519169, -0.157382 )
vector[40] = ( 0.152874, 0.405328, 0.335170 )
```

For peer review only

```
vector[41] = ( 0.109576, 0.536320, 0.018825 )
vector[42] = ( -0.045652, 0.300780, 0.455464 )
vector[43] = ( 0.000000, 0.000000, 0.000000 )
vector[44] = ( -0.533887, 0.114345, 0.043471 )
vector[45] = ( -0.097529, 0.434255, -0.319235 )
vector[46] = ( 0.391774, -0.236122, -0.301263 )
vector[47] = ( 0.399513, -0.317429, 0.199068 )
vector[48] = ( 0.200167, 0.067226, 0.505385 )
vector[49] = ( 0.385668, -0.387145, 0.037137 )
vector[50] = ( 0.059543, 0.145424, 0.524697 )
vector[51] = ( -0.445546, -0.189946, 0.255752 )
vector[52] = ( 0.263180, -0.007998, -0.480284 )
vector[53] = ( -0.375132, -0.375662, 0.134735 )
vector[54] = ( 0.000000, 0.000000, 0.000000 )
vector[55] = ( -0.100958, 0.513042, -0.163080 )
vector[56] = ( 0.266095, 0.478340, 0.019604 )
vector[57] = ( 0.480516, -0.133538, -0.226434 )
vector[58] = ( 0.253431, -0.482875, 0.051025 )
vector[59] = ( 0.361384, -0.227994, 0.342667 )
vector[60] = ( -0.479164, -0.248769, 0.092279 )
vector[61] = ( -0.422438, -0.343026, -0.062282 )
vector[62] = ( 0.525823, 0.037772, -0.148605 )
vector[63] = ( 0.112166, -0.092301, 0.528109 )
vector[64] = ( 0.050487, -0.545354, 0.006363 )
vector[65] = ( 0.000000, 0.000000, 0.000000 )
vector[66] = ( -0.290577, 0.355116, 0.299095 )
vector[67] = ( -0.303506, -0.415037, -0.188755 )
vector[68] = ( -0.340501, 0.129187, 0.409109 )
vector[69] = ( -0.275521, -0.188617, -0.434179 )
vector[70] = ( 0.148849, 0.097956, -0.517928 )
```

For peer review only

```
# Author: qspace2siemens.m (Michael Thrippleton), manually edited
into 2 parts
# Source file: ./vector_tables/neonate/04-shells-3-6-64-64.txt
# b-value at UI: 2500
# non-zero b-values: 200   500  2500
# number of non-zero shells: 2
# number of directions per non-zero shell: 3   6  64
# number of b=0 volumes: 7
# total number of directions including b0: 151
[directions=80]
normalization = none
coordinatesystem = xyz
comment=bUI: 2500, b: 200   500   2500, Nb0: 7
vector[0] = ( 0.000000, 0.000000, 0.000000 )
vector[1] = ( 0.252007, 0.053675, -0.116668 )
vector[2] = ( 0.118341, -0.013011, 0.256566 )
vector[3] = ( 0.047528, -0.276133, -0.038625 )
vector[4] = ( -0.303298, -0.002700, -0.328638 )
vector[5] = ( -0.128927, -0.159163, 0.397549 )
vector[6] = ( 0.288240, 0.341931, 0.000938 )
vector[7] = ( -0.166829, 0.397185, -0.120052 )
vector[8] = ( -0.069301, 0.303423, 0.321142 )
vector[9] = ( 0.425645, -0.074339, -0.115324 )
vector[10] = ( 0.391424, -0.221918, 0.893051 )
vector[11] = ( 0.458593, -0.241695, -0.855147 )
vector[12] = ( 0.354539, 0.919288, 0.170913 )
vector[13] = ( 0.495263, -0.780339, -0.381819 )
vector[14] = ( -0.574230, 0.458191, 0.678470 )
vector[15] = ( 0.000000, 0.000000, 0.000000 )
vector[16] = ( -0.188453, -0.033220, -0.981520 )
vector[17] = ( 0.594951, -0.772279, 0.222754 )
vector[18] = ( 0.076963, -0.202692, -0.976213 )
vector[19] = ( -0.354234, 0.663631, 0.658872 )
vector[20] = ( -0.245839, 0.923577, 0.294225 )
vector[21] = ( -0.646526, -0.378550, -0.662347 )
vector[22] = ( 0.782685, 0.616196, -0.087788 )
vector[23] = ( -0.102171, -0.675368, -0.730369 )
vector[24] = ( -0.593833, 0.627627, -0.503435 )
vector[25] = ( -0.289839, 0.954652, -0.068065 )
vector[26] = ( 0.000000, 0.000000, 0.000000 )
vector[27] = ( 0.932852, 0.268018, -0.240735 )
vector[28] = ( -0.292661, 0.011816, 0.956143 )
vector[29] = ( -0.125932, -0.877649, -0.462465 )
vector[30] = ( 0.287138, 0.947828, -0.138468 )
vector[31] = ( -0.400507, -0.785392, -0.471967 )
vector[32] = ( 0.046561, 0.178494, -0.982839 )
vector[33] = ( 0.774106, -0.243372, -0.584405 )
vector[34] = ( -0.709331, 0.570685, 0.413724 )
vector[35] = ( 0.258673, -0.649858, 0.714684 )
vector[36] = ( 0.000000, 0.000000, 0.000000 )
vector[37] = ( 0.812504, 0.520520, 0.262482 )
vector[38] = ( -0.551995, -0.116325, -0.825694 )
vector[39] = ( -0.680119, 0.223136, -0.698319 )
vector[40] = ( -0.848362, -0.280672, -0.448893 )
```

For peer review only

```
vector[41] = ( -0.460227, -0.230447, 0.857371 )
vector[42] = ( 0.639224, 0.615748, 0.460703 )
vector[43] = ( 0.953358, -0.285443, 0.098132 )
vector[44] = ( -0.501430, 0.459528, -0.733077 )
vector[45] = ( 0.922461, 0.385130, 0.027209 )
vector[46] = ( -0.815410, 0.546002, -0.192323 )
vector[47] = ( 0.000000, 0.000000, 0.000000 )
vector[48] = ( -0.924442, 0.129694, -0.358591 )
vector[49] = ( 0.549990, 0.820347, -0.156657 )
vector[50] = ( 0.774802, 0.509647, -0.374089 )
vector[51] = ( 0.907672, -0.355700, -0.222731 )
vector[52] = ( 0.051712, 0.985317, 0.162714 )
vector[53] = ( -0.970546, -0.135098, -0.199471 )
vector[54] = ( -0.621107, -0.417526, 0.663249 )
vector[55] = ( -0.776136, 0.621968, 0.103774 )
vector[56] = ( 0.551897, -0.830144, -0.079188 )
vector[57] = ( 0.555009, 0.711394, -0.431142 )
vector[58] = ( 0.000000, 0.000000, 0.000000 )
vector[59] = ( -0.239295, 0.451777, 0.859439 )
vector[60] = ( -0.325801, -0.314211, -0.891698 )
vector[61] = ( 0.649939, -0.012663, -0.759881 )
vector[62] = ( -0.042327, 0.894181, -0.445699 )
vector[63] = ( -0.159022, 0.408833, -0.898648 )
vector[64] = ( 0.388219, 0.606776, -0.693620 )
vector[65] = ( -0.329997, 0.825600, -0.457697 )
vector[66] = ( 0.060764, 0.443276, 0.894323 )
vector[67] = ( -0.794452, 0.390958, -0.464756 )
vector[68] = ( -0.392295, -0.567128, -0.724204 )
vector[69] = ( 0.000000, 0.000000, 0.000000 )
vector[70] = ( 0.272234, 0.851327, -0.448477 )
vector[71] = ( 0.785891, 0.193927, -0.587169 )
vector[72] = ( -0.145787, 0.828569, 0.540573 )
vector[73] = ( 0.616784, 0.765973, 0.181281 )
vector[74] = ( -0.808755, -0.029868, -0.587387 )
vector[75] = ( 0.997247, -0.010658, -0.073384 )
vector[76] = ( -0.152743, -0.477444, 0.865284 )
vector[77] = ( -0.040188, -0.715882, 0.697064 )
vector[78] = ( -0.907740, 0.040990, 0.417525 )
vector[79] = ( 0.008357, -0.985450, 0.169758 )
```

For peer review only

SIEMENS MAGNETOM Prisma

\\Study Protocols\BRAIN\Neonates\Theirworld - E161723 - MT_test\MTSatOn_neonate_v2
TA: 2:58 PM: REF Voxel size: 2.0×2.0×2.0 mmPAT: 3 Rel. SNR: 1.00 : qfl

Properties

Prio recon	Off
Load images to viewer	On
Inline movie	Off
Auto store images	On
Load images to stamp segments	Off
Load images to graphic segments	Off
Auto open inline display	Off
Auto close inline display	Off
Start measurement without further preparation	Off
Wait for user to start	Off
Start measurements	Single measurement

Routine

Slab group	1
Slabs	1
Dist. factor	20 %
Position	R6.7 P19.4 H34.5 mm
Orientation	Sagittal
Phase enc. dir.	A >> P
AutoAlign	---
Phase oversampling	0 %
Slice oversampling	0.0 %
Slices per slab	72
FoV read	128 mm
FoV phase	121.9 %
Slice thickness	2.00 mm
TR	75.0 ms
TE 1	1.54 ms
TE 2	4.55 ms
TE 3	8.56 ms
Averages	1
Concatenations	1
Filter	Prescan Normalize
Coil elements	PeH;PeN

Contrast - Common

TR	75.0 ms
TE 1	1.54 ms
TE 2	4.55 ms
TE 3	8.56 ms
MTC	On
Magn. preparation	None
Flip angle	5 deg
Fat suppr.	None
Water suppr.	None
SWI	Off

Contrast - Dynamic

Averages	1
Averaging mode	Short term
Reconstruction	Magnitude
Measurements	1
Multiple series	Each measurement

Resolution - Common

FoV read	128 mm
FoV phase	121.9 %
Slice thickness	2.00 mm
Base resolution	64

Resolution - Common

Phase resolution	100 %
Slice resolution	100 %
Phase partial Fourier	6/8
Slice partial Fourier	Off
Interpolation	Off

Resolution - iPAT

PAT mode	GRAPPA
Accel. factor PE	3
Ref. lines PE	24
Accel. factor 3D	1
Reference scan mode	Integrated

Resolution - Filter Image

Image Filter	Off
Distortion Corr.	Off
Prescan Normalize	On
Unfiltered images	Off
Normalize	Off
B1 filter	Off

Resolution - Filter Rawdata

Raw filter	Off
Elliptical filter	Off

Geometry - Common

Slab group	1
Slabs	1
Dist. factor	20 %
Position	R6.7 P19.4 H34.5 mm
Orientation	Sagittal
Phase enc. dir.	A >> P
Slice oversampling	0.0 %
Slices per slab	72
FoV read	128 mm
FoV phase	121.9 %
Slice thickness	2.00 mm
TR	75.0 ms
Multi-slice mode	Interleaved
Series	Interleaved
Concatenations	1

Geometry - AutoAlign

Slab group	1
Position	R6.7 P19.4 H34.5 mm
Orientation	Sagittal
Phase enc. dir.	A >> P
AutoAlign	---
Initial Position	R6.7 P19.4 H34.5
R	6.7 mm
P	19.4 mm
H	34.5 mm
Initial Rotation	0.00 deg
Initial Orientation	Sagittal

Geometry - Saturation

Saturation mode	Standard
Fat suppr.	None
Water suppr.	None
Special sat.	None

For peer review only

SIEMENS MAGNETOM Prisma

Geometry - Tim Planning Suite

Set-n-Go Protocol	Off
Table position	H
Table position	0 mm
Inline Composing	Off

System - Miscellaneous

Positioning mode	REF
Table position	H
Table position	0 mm
MSMA	S - C - T
Sagittal	R >> L
Coronal	A >> P
Transversal	F >> H
Coil Combine Mode	Sum of Squares
Save uncombined	Off
Matrix Optimization	Off
AutoAlign	---
Coil Select Mode	Off - AutoCoilSelect

System - Adjustments

B0 Shim mode	Tune up
B1 Shim mode	TrueForm
Adjust with body coil	Off
Confirm freq. adjustment	Off
Assume Dominant Fat	Off
Assume Silicone	Off
Adjustment Tolerance	Auto

System - Adjust Volume

Position	Isocenter
Orientation	Transversal
Rotation	0.00 deg
A >> P	263 mm
R >> L	350 mm
F >> H	350 mm
Reset	Off

System - pTx Volumes

B1 Shim mode	TrueForm
Excitation	Non-sel.

System - Tx/Rx

Frequency 1H	123.244480 MHz
Correction factor	1
Gain	Low
Img. Scale Cor.	3.000
Reset	Off
? Ref. amplitude 1H	0.000 V

Physio - Signal1

1st Signal/Mode	None
TR	75.0 ms
Concatenations	1
Segments	1

Physio - Cardiac

Tagging	None
Magn. preparation	None
Fat suppr.	None
Dark blood	Off
FoV read	128 mm
FoV phase	121.9 %
Phase resolution	100 %

Physio - PACE

Resp. control	Off
Concatenations	1

Inline - Common

Subtract	Off
Measurements	1
StdDev	Off
Liver registration	Off
Save original images	On

Inline - MIP

MIP-Sag	Off
MIP-Cor	Off
MIP-Tra	Off
MIP-Time	Off
Save original images	On

Inline - Soft Tissue

Wash - In	Off
Wash - Out	Off
TTP	Off
PEI	Off
MIP - time	Off
Measurements	1

Inline - Composing

Inline Composing	Off
Distortion Corr.	Off

Inline - MapIt

Save original images	On
MapIt	None
Flip angle	5 deg
Measurements	1
Contrasts	3
TR	75.0 ms
TE 1	1.54 ms
TE 2	4.55 ms
TE 3	8.56 ms

Sequence - Part 1

Introduction	Off
Dimension	3D
Elliptical scanning	On
Phase stabilisation	Off
Asymmetric echo	Off
Contrasts	3
Flow comp. 1	No
Readout mode	Bipolar
Multi-slice mode	Interleaved
Bandwidth 1	580 Hz/Px
Bandwidth 2	580 Hz/Px
Bandwidth 3	580 Hz/Px

Sequence - Part 2

Segments	1
Acoustic noise reduction	Active
RF pulse type	Low SAR
Gradient mode	Normal
Excitation	Non-sel.
RF spoiling	On

For peer review only

SIEMENS MAGNETOM Prisma

Sequence - Assistant

Mode	Off
Allowed delay	30 s

For peer review only

For peer review only - http://bmjopen.bmj.com/site/about/guidelines.xhtml

SIEMENS MAGNETOM Prisma

\\Study Protocols\BRAIN\Neonates\Theirworld - E161723 - MT_test\MTSatOff_neonate_v2

TA: 2:58 PM: FIX Voxel size: 2.0×2.0×2.0 mmPAT: 3 Rel. SNR: 1.00 : qfl

Properties

Prio recon	Off
Load images to viewer	On
Inline movie	Off
Auto store images	On
Load images to stamp segments	Off
Load images to graphic segments	Off
Auto open inline display	Off
Auto close inline display	Off
Start measurement without further preparation	On
Wait for user to start	Off
Start measurements	Single measurement

Routine

Slab group	1
Slabs	1
Dist. factor	20 %
Position	R6.7 P19.4 H34.5 mm
Orientation	Sagittal
Phase enc. dir.	A >> P
AutoAlign	---
Phase oversampling	0 %
Slice oversampling	0.0 %
Slices per slab	72
FoV read	128 mm
FoV phase	121.9 %
Slice thickness	2.00 mm
TR	75.0 ms
TE 1	1.54 ms
TE 2	4.55 ms
TE 3	8.56 ms
Averages	1
Concatenations	1
Filter	Prescan Normalize
Coil elements	PeH;PeN

Contrast - Common

TR	75.0 ms
TE 1	1.54 ms
TE 2	4.55 ms
TE 3	8.56 ms
MTC	Off
Magn. preparation	None
Flip angle	5 deg
Fat suppr.	None
Water suppr.	None
SWI	Off

Contrast - Dynamic

Averages	1
Averaging mode	Short term
Reconstruction	Magnitude
Measurements	1
Multiple series	Each measurement

Resolution - Common

FoV read	128 mm
FoV phase	121.9 %
Slice thickness	2.00 mm
Base resolution	64

Resolution - Common

Phase resolution	100 %
Slice resolution	100 %
Phase partial Fourier	6/8
Slice partial Fourier	Off
Interpolation	Off

Resolution - iPAT

PAT mode	GRAPPA
Accel. factor PE	3
Ref. lines PE	24
Accel. factor 3D	1
Reference scan mode	Integrated

Resolution - Filter Image

Image Filter	Off
Distortion Corr.	Off
Prescan Normalize	On
Unfiltered images	Off
Normalize	Off
B1 filter	Off

Resolution - Filter Rawdata

Raw filter	Off
Elliptical filter	Off

Geometry - Common

Slab group	1
Slabs	1
Dist. factor	20 %
Position	R6.7 P19.4 H34.5 mm
Orientation	Sagittal
Phase enc. dir.	A >> P
Slice oversampling	0.0 %
Slices per slab	72
FoV read	128 mm
FoV phase	121.9 %
Slice thickness	2.00 mm
TR	75.0 ms
Multi-slice mode	Interleaved
Series	Interleaved
Concatenations	1

Geometry - AutoAlign

Slab group	1
Position	R6.7 P19.4 H34.5 mm
Orientation	Sagittal
Phase enc. dir.	A >> P
AutoAlign	---
Initial Position	R6.7 P19.4 H34.5
R	6.7 mm
P	19.4 mm
H	34.5 mm
Initial Rotation	0.00 deg
Initial Orientation	Sagittal

Geometry - Saturation

Saturation mode	Standard
Fat suppr.	None
Water suppr.	None
Special sat.	None

For peer review only

SIEMENS MAGNETOM Prisma

Geometry - Tim Planning Suite

Set-n-Go Protocol	Off
Table position	H
Table position	0 mm
Inline Composing	Off

System - Miscellaneous

Positioning mode	FIX
Table position	H
Table position	0 mm
MSMA	S - C - T
Sagittal	R >> L
Coronal	A >> P
Transversal	F >> H
Coil Combine Mode	Sum of Squares
Save uncombined	Off
Matrix Optimization	Off
AutoAlign	---
Coil Select Mode	Off - AutoCoilSelect

System - Adjustments

B0 Shim mode	Tune up
B1 Shim mode	TrueForm
Adjust with body coil	Off
Confirm freq. adjustment	Off
Assume Dominant Fat	Off
Assume Silicone	Off
Adjustment Tolerance	Auto

System - Adjust Volume

Position	Isocenter
Orientation	Transversal
Rotation	0.00 deg
A >> P	263 mm
R >> L	350 mm
F >> H	350 mm
Reset	Off

System - pTx Volumes

B1 Shim mode	TrueForm
Excitation	Non-sel.

System - Tx/Rx

Frequency 1H	123.244480 MHz
Correction factor	1
Gain	Low
Img. Scale Cor.	3.000
Reset	Off
? Ref. amplitude 1H	0.000 V

Physio - Signal1

1st Signal/Mode	None
TR	75.0 ms
Concatenations	1
Segments	1

Physio - Cardiac

Tagging	None
Magn. preparation	None
Fat suppr.	None
Dark blood	Off
FoV read	128 mm
FoV phase	121.9 %
Phase resolution	100 %

Physio - PACE

Resp. control	Off
Concatenations	1

Inline - Common

Subtract	Off
Measurements	1
StdDev	Off
Liver registration	Off
Save original images	On

Inline - MIP

MIP-Sag	Off
MIP-Cor	Off
MIP-Tra	Off
MIP-Time	Off
Save original images	On

Inline - Soft Tissue

Wash - In	Off
Wash - Out	Off
TTP	Off
PEI	Off
MIP - time	Off
Measurements	1

Inline - Composing

Inline Composing	Off
Distortion Corr.	Off

Inline - MapIt

Save original images	On
MapIt	None
Flip angle	5 deg
Measurements	1
Contrasts	3
TR	75.0 ms
TE 1	1.54 ms
TE 2	4.55 ms
TE 3	8.56 ms

Sequence - Part 1

Introduction	Off
Dimension	3D
Elliptical scanning	On
Phase stabilisation	Off
Asymmetric echo	Off
Contrasts	3
Flow comp. 1	No
Readout mode	Bipolar
Multi-slice mode	Interleaved
Bandwidth 1	580 Hz/Px
Bandwidth 2	580 Hz/Px
Bandwidth 3	580 Hz/Px

Sequence - Part 2

Segments	1
Acoustic noise reduction	Active
RF pulse type	Low SAR
Gradient mode	Normal
Excitation	Non-sel.
RF spoiling	On

For peer review only

SIEMENS MAGNETOM Prisma

Sequence - Assistant

Mode	Off
Allowed delay	30 s

For peer review only

For peer review only - http://bmjopen.bmj.com/site/about/guidelines.xhtml

SIEMENS MAGNETOM Prisma

\\Study Protocols\BRAIN\Neonates\Theirworld - E161723 - MT_test\MTSatT1_neonate_v2

TA: 0:36 PM: FIX Voxel size: 2.0×2.0×2.0 mmPAT: 3 Rel. SNR: 1.00 : qfl

Properties

Prio recon	Off
Load images to viewer	On
Inline movie	Off
Auto store images	On
Load images to stamp segments	Off
Load images to graphic segments	Off
Auto open inline display	Off
Auto close inline display	Off
Start measurement without further preparation	On
Wait for user to start	Off
Start measurements	Single measurement

Routine

Slab group	1
Slabs	1
Dist. factor	20 %
Position	R6.7 P19.4 H34.5 mm
Orientation	Sagittal
Phase enc. dir.	A >> P
AutoAlign	---
Phase oversampling	0 %
Slice oversampling	0.0 %
Slices per slab	72
FoV read	128 mm
FoV phase	121.9 %
Slice thickness	2.00 mm
TR	15.0 ms
TE 1	1.54 ms
TE 2	4.55 ms
TE 3	8.56 ms
Averages	1
Concatenations	1
Filter	Prescan Normalize
Coil elements	PeH;PeN

Contrast - Common

TR	15.0 ms
TE 1	1.54 ms
TE 2	4.55 ms
TE 3	8.56 ms
MTC	Off
Magn. preparation	None
Flip angle	14 deg
Fat suppr.	None
Water suppr.	None
SWI	Off

Contrast - Dynamic

Averages	1
Averaging mode	Short term
Reconstruction	Magnitude
Measurements	1
Multiple series	Each measurement

Resolution - Common

FoV read	128 mm
FoV phase	121.9 %
Slice thickness	2.00 mm
Base resolution	64

Resolution - Common

Phase resolution	100 %
Slice resolution	100 %
Phase partial Fourier	6/8
Slice partial Fourier	Off
Interpolation	Off

Resolution - iPAT

PAT mode	GRAPPA
Accel. factor PE	3
Ref. lines PE	24
Accel. factor 3D	1
Reference scan mode	Integrated

Resolution - Filter Image

Image Filter	Off
Distortion Corr.	Off
Prescan Normalize	On
Unfiltered images	Off
Normalize	Off
B1 filter	Off

Resolution - Filter Rawdata

Raw filter	Off
Elliptical filter	Off

Geometry - Common

Slab group	1
Slabs	1
Dist. factor	20 %
Position	R6.7 P19.4 H34.5 mm
Orientation	Sagittal
Phase enc. dir.	A >> P
Slice oversampling	0.0 %
Slices per slab	72
FoV read	128 mm
FoV phase	121.9 %
Slice thickness	2.00 mm
TR	15.0 ms
Multi-slice mode	Interleaved
Series	Interleaved
Concatenations	1

Geometry - AutoAlign

Slab group	1
Position	R6.7 P19.4 H34.5 mm
Orientation	Sagittal
Phase enc. dir.	A >> P
AutoAlign	---
Initial Position	R6.7 P19.4 H34.5
R	6.7 mm
P	19.4 mm
H	34.5 mm
Initial Rotation	0.00 deg
Initial Orientation	Sagittal

Geometry - Saturation

Saturation mode	Standard
Fat suppr.	None
Water suppr.	None
Special sat.	None

For peer review only

SIEMENS MAGNETOM Prisma

Geometry - Tim Planning Suite

Set-n-Go Protocol	Off
Table position	H
Table position	0 mm
Inline Composing	Off

System - Miscellaneous

Positioning mode	FIX
Table position	H
Table position	0 mm
MSMA	S - C - T
Sagittal	R >> L
Coronal	A >> P
Transversal	F >> H
Coil Combine Mode	Sum of Squares
Save uncombined	Off
Matrix Optimization	Off
AutoAlign	---
Coil Select Mode	Off - AutoCoilSelect

System - Adjustments

B0 Shim mode	Tune up
B1 Shim mode	TrueForm
Adjust with body coil	Off
Confirm freq. adjustment	Off
Assume Dominant Fat	Off
Assume Silicone	Off
Adjustment Tolerance	Auto

System - Adjust Volume

Position	Isocenter
Orientation	Transversal
Rotation	0.00 deg
A >> P	263 mm
R >> L	350 mm
F >> H	350 mm
Reset	Off

System - pTx Volumes

B1 Shim mode	TrueForm
Excitation	Non-sel.

System - Tx/Rx

Frequency 1H	123.244480 MHz
Correction factor	1
Gain	Low
Img. Scale Cor.	3.000
Reset	Off
? Ref. amplitude 1H	0.000 V

Physio - Signal1

1st Signal/Mode	None
TR	15.0 ms
Concatenations	1
Segments	1

Physio - Cardiac

Tagging	None
Magn. preparation	None
Fat suppr.	None
Dark blood	Off
FoV read	128 mm
FoV phase	121.9 %
Phase resolution	100 %

Physio - PACE

Resp. control	Off
Concatenations	1

Inline - Common

Subtract	Off
Measurements	1
StdDev	Off
Liver registration	Off
Save original images	On

Inline - MIP

MIP-Sag	Off
MIP-Cor	Off
MIP-Tra	Off
MIP-Time	Off
Save original images	On

Inline - Soft Tissue

Wash - In	Off
Wash - Out	Off
TTP	Off
PEI	Off
MIP - time	Off
Measurements	1

Inline - Composing

Inline Composing	Off
Distortion Corr.	Off

Inline - MapIt

Save original images	On
MapIt	None
Flip angle	14 deg
Measurements	1
Contrasts	3
TR	15.0 ms
TE 1	1.54 ms
TE 2	4.55 ms
TE 3	8.56 ms

Sequence - Part 1

Introduction	Off
Dimension	3D
Elliptical scanning	On
Phase stabilisation	Off
Asymmetric echo	Off
Contrasts	3
Flow comp. 1	No
Readout mode	Bipolar
Multi-slice mode	Interleaved
Bandwidth 1	580 Hz/Px
Bandwidth 2	580 Hz/Px
Bandwidth 3	580 Hz/Px

Sequence - Part 2

Segments	1
Acoustic noise reduction	Active
RF pulse type	Low SAR
Gradient mode	Normal
Excitation	Non-sel.
RF spoiling	On

For peer review only

SIEMENS MAGNETOM Prisma

Sequence - Assistant

Mode	Off
Allowed delay	30 s

For peer review only

For peer review only - http://bmjopen.bmj.com/site/about/guidelines.xhtml
